# A theoretical single-parameter model for urbanisation to study infectious disease spread and interventions

**DOI:** 10.1371/journal.pcbi.1006879

**Published:** 2019-03-07

**Authors:** Solveig Engebretsen, Kenth Engø-Monsen, Arnoldo Frigessi, Birgitte Freiesleben de Blasio

**Affiliations:** 1 Oslo Centre for Biostatistics and Epidemiology, Department of Biostatistics, Institute of Basic Medical Sciences, University of Oslo, Oslo, Norway; 2 Department of Infectious Disease Epidemiology and Modelling, Division for Infection Control and Environmental Health, Norwegian Institute of Public Health, Oslo, Norway; 3 Telenor Research, Telenor Group, Fornebu, Norway; 4 Oslo Centre for Biostatistics and Epidemiology, Oslo University Hospital, Oslo, Norway; London School of Hygiene & Tropical Medicine, UNITED KINGDOM

## Abstract

The world is continuously urbanising, resulting in clusters of densely populated urban areas and more sparsely populated rural areas. We propose a method for generating spatial fields with controllable levels of clustering of the population. We build a synthetic country, and use this method to generate versions of the country with different clustering levels. Combined with a metapopulation model for infectious disease spread, this allows us to in silico explore how urbanisation affects infectious disease spread. In a baseline scenario with no interventions, the underlying population clustering seems to have little effect on the final size and timing of the epidemic. Under within-country restrictions on non-commuting travel, the final size decreases with increased population clustering. The effect of travel restrictions on reducing the final size is larger with higher clustering. The reduction is larger in the more rural areas. Within-country travel restrictions delay the epidemic, and the delay is largest for lower clustering levels. We implemented three different vaccination strategies—uniform vaccination (in space), preferentially vaccinating urban locations and preferentially vaccinating rural locations. The urban and uniform vaccination strategies were most effective in reducing the final size, while the rural vaccination strategy was clearly inferior. Visual inspection of some European countries shows that many countries already have high population clustering. In the future, they will likely become even more clustered. Hence, according to our model, within-country travel restrictions are likely to be less and less effective in delaying epidemics, while they will be more effective in decreasing final sizes. In addition, to minimise final sizes, it is important not to neglect urban locations when distributing vaccines. To our knowledge, this is the first study to systematically investigate the effect of urbanisation on infectious disease spread and in particular, to examine effectiveness of prevention measures as a function of urbanisation.

## Author summary

We study the interplay between urbanisation and infectious disease spread. As part of the worldwide urbanisation process, people are continuously moving to urban areas, and the cities are growing in size. This causes clusters of areas with high population density and clusters of areas with low population density, which is what we call population clustering. By simulating infectious disease spread in a synthetic country where we vary this population clustering, we explore the consequences of urbanisation on infectious disease spread. Our qualitative results have direct implications for infectious disease control guidelines and policies. We find that implementing internal travel restrictions have greater impact on the final number ill in the most urbanised countries than in the less urbanised countries. The effect is largest in the more rural parts of the country. According to our model, travel restrictions are more effective in delaying the epidemic in the less urbanised countries than in the more urbanised countries. We investigate vaccination strategies, where locations are targeted depending on how urban or rural they are. We find that it is important to vaccinate the urban locations—if the most urban locations are not covered by the vaccine, the final number ill will be a lot larger.

## Introduction

We are living in a world which is continuously urbanising. From a United Nations report [1], we know that in 2014, 54% of the population was living in urban areas. By 2050, they estimate that 66% of the population will be living in urban areas. The number of “megacities” is also increasing [1]. Urbanisation involves clustering of people within a geographical area [2]. Migration from rural to urban areas is one of the key drivers of urbanisation, and results in spatial expansion of urban centers [3]. Though there are also other characteristics of urbanisation, like urban sprawl [2, 3] and population growth, we here focus on this population clustering. By population clustering, we mean that large cities are often surrounded by other cities or suburbs with large population sizes. Rural areas also tend to appear in clusters (i.e. positioned closely together). This has for instance been found to be the case for the Turku region in Finland, where regions of both high and low population densities are clustered [4]. Another example is Australia, where the majority of the population is clustered around the coastal belt [5]. From here on, the term urbanisation will be used to refer to the population clustering.

We aim at studying the urbanisation phenomenon and its effect on infectious disease spread in a general setting. Our purpose is not to describe single outbreaks in specific populations, or finding the “best model” or strategy for a specific country, but rather study the phenomenon from a more generic and principled point of view.

In this paper, we explore the effect of internal travel restrictions and vaccination on infectious disease spread, when the clustering of population is continuously varied. By internal travel restrictions, we will refer to restrictions on non-commuting travel within the country. If the disease dynamics is different for different levels of population clustering, this can have important implications for the effectiveness and design of interventions. In order to study this, we need a continuous series of countries where everything is fixed, apart from the urbanisation, which changes between the countries in a controllable and continuous manner. This is difficult in practice, and we therefore construct a fictional country for this purpose, however trying to maintain some realism. Our aim is not to develop a precise model for urbanisation in a country. We aim for a simple model, where urbanisation is controlled by one single tuning parameter, which captures key features of urbanisation. We use this model to generate a fictional country with a plausible population distributed in urban and rural areas. More specifically, we use a plausible population size distribution, plausible commuting patterns modelled by a gravity law and a plausible infectious disease model. We use a metapopulation infectious disease model, where an SEIR (susceptible, exposed, infectious, recovered) process [6] governs the disease dynamics in each location, and the different locations are coupled through individuals travelling between them. This framework allows us to investigate in silico how urbanisation affects various aspects of infectious disease spread. As a motivating example, we will study an influenza-like illness spreading in a single country, where we assume that the pathogen has already been imported to the country. We investigate how the infectious disease dynamics depends on the underlying population clustering in the country and focus on the effect of internal travel restrictions and three different vaccination strategies.

The plausibility of the synthetic country is obtained by conserving the population size distribution of Norway and a gravity law fitted to data on commuting between Norwegian municipalities. We use Norwegian data to ensure reasonable population sizes, and plausible commuting for those population sizes, and because we have commuting data available on a relatively fine spatial scale. We are studying the urbanisation phenomenon generically, and we do not claim, nor is it our aim, that these results are directly applicable to Norway. This framework is a simulator, and it is not our purpose to model and provide results for a specific country. Our results allow a theoretical description of how urbanisation affects interventions to control epidemics.

To our knowledge, this is the first modelling and simulation study which systematically investigates the effect of population clustering on the spread of infectious diseases. In particular, it is the first study to consider the effect of internal travel restrictions and vaccination in relation to urbanisation. This is a study of the urbanisation phenomenon represented in a very simplified and theoretical way, yet with important elements of realism. We focus on these two control strategies, because they are clearly affected by urbanisation. We do not consider international air travel restrictions, school closure or other sanitary measures, which were used for instance during the 2009 pandemic [7, 8]. Travel restrictions have a long history, and date back at least to the 14th century, where people were prevented from leaving or entering specific communities during the plague epidemics [9]. Travel bans were also used in many cities and countries during the 1918-1919 influenza epidemic [9]. More recently, internal travel restrictions were used as a mitigation measure against influenza virus transmission during the 2009 pandemic in Mongolia, through interruption of provincial rail and road travel [10], and during the recent Ebola outbreaks [11]. There has been some work on infectious disease spread and urbanisation focussing on the improved health conditions in urban areas compared to rural areas. For the developed countries, health has overall improved with increased urbanisation [12]. In low-income settings, health conditions are on average better in urban areas than in rural areas, but there are also significant challenges relating to inequities and heterogeneity in health among the urban population (favelas, slums, etc.). High population density increases exposure to infectious diseases [12]. In Africa, the urban population has better nutritional status, fewer morbid events, increased vaccine coverage and better access to healthcare services compared to the rural population, and have reduced levels of malaria transmission and other severe diseases [13]. There is also one study, [14], which develops a model for the effect of urbanisation on the transmission of infectious diseases, focussing on population growth and land use development. However, the infectious disease spread model is very simple. In addition, they do not consider the effect of interventions.

The effectiveness of both vaccination and internal travel restrictions on mitigating an infectious disease have been studied in various settings, e.g. [9, 10, 15–20]. Germann et al. [15] study the spread of a hypothetical pandemic influenza, with a basic reproductive number in the range 1.6-2.4, in the United States, and find that (domestic) travel restrictions do not have an effect on the final size of the epidemic, but might be able to slightly delay the time course. They also find that vaccination drastically reduces the final number of cases and delays the spread. Ferguson et al. [16] find that reducing long distance travel within the United States (domestic air travel) only slightly delays the influenza epidemic, for a hypothetical pandemic influenza strain with varying transmissibility. They consider vaccination in both the United Kingdom and the United States, and find that vaccination significantly reduces the final size of the epidemic. In accordance with these studies, the US Department of Health and Human Services also claims that vaccination is the most effective way of preventing the public health impact of (pandemic) influenza [21]. In a review study, Mateus et al. [17] find that domestic travel restrictions can delay the influenza epidemics by one week, and that extensive travel restrictions can reduce the final size of the epidemic. Both seasonal and pandemic influenza strains are considered. They also find that travel restrictions have minimal impact in urban centers with dense population and high mobility. Brownstein et al. [18] consider the influenza epidemic following the travel ban after 9/11. They claim that the decrease in air traffic in the United States caused a delayed and prolonged influenza season (however, note also the rebuttal in [22]). For a hypothetical influenza strain in Korea, it was found that 50% internal travel restrictions delayed the peak timing and had a slight reduction effect on the peak [19]. Some work has also been done on mathematical explanations and expressions for delay in epidemic spreading due to travel restrictions, for border control on international travel [23], and for more general mobility networks [7, 24].

During the 2009 influenza pandemic, uniform vaccination guidelines were given (i.e. pro rata), for instance in Norway [25], Ireland [26] and the United States [27, 28], and the default vaccination strategies are usually uniform [29]. However, the effectiveness of the different vaccination strategies and which strategy is best in terms of minimising attack rate, possibly depend on the population clustering of the underlying country. We thus simulate the epidemic with three different vaccination strategies—uniform vaccination, prioritising urban locations and prioritising rural locations. This allows us to compare the three vaccination strategies as a function of population clustering and provides us with a better understanding and possibilities for refined vaccination strategies. There are numerous examples of spatially targeted vaccination and antiviral strategies in the literature [29–39]. Some of these are based on dynamic optimisation strategies [29, 31, 33, 39], while others are based on prioritising locations with high prevalence (i.e. geographically targeting hotspots) [30, 34]. In [38], pro rata vaccination strategies are compared to vaccination strategies prioritising locations sequentially by population size (among other vaccination strategies). This is similar to the vaccination strategy we investigate, targeting urban and/or rural locations.

We investigate how domestic travel restrictions and vaccination affects the epidemic timing, spread and final size for the various levels of population clustering. For policy planners, the timing of peak dates and initial dates are important because they indicate how much time there is to implement interventions and preventive measures. If peak dates for spatially proximate regions are close in time, the efficiency of the health care organisation is challenged. This is extra problematic if there is in addition clustering of high peak incidences, since that would imply that spatially proximate locations have high disease activity at the same time. We further disentangle the effect of travel restrictions on final size of the epidemic by examining how the effect depends on how urban or rural the location is.

We first describe the simulation set-up with details on the properties of the fictional country, the clustering algorithm, the disease dynamics model, the travel restrictions and vaccination strategies. We then use this tool to simulate the infectious disease spread for the various levels of clustering, investigate the effect of various amounts of internal travel restrictions, simulate and investigate the effect of the three vaccination strategies and finally a combination of travel restrictions and vaccination. We end with discussion and concluding remarks.

## Models

In order to investigate how population clustering affects infectious disease dynamics, we build a series of countries with varying population clustering, where everything else is fixed. Imagine something like taking one country and reshuffling its population, so to increase monotonically its level of urbanisation. We construct a fictional country, where the spatial clustering of population sizes is controlled by a design parameter. However, we try to conserve some realism in the different ingredients of the framework. The realism is obtained by using a population size distribution and commuting law fitted to data from Norwegian municipalities.

In this section, the different parts of the simulation framework will be described. We first describe how to generate the geographical areas, with a plausible population distribution. We then introduce the clustering algorithm that is used to generate different versions of the geographical area, with different levels of clustering. Then we describe the models for the two (coupled) dynamical processes for the geographical areas—the infectious disease process and the mobility process. Finally, we describe the interventions—the internal travel restrictions and the vaccination strategies.

### Generating the geographical areas

We construct a country, where the spatial clustering of population sizes is controlled by a design parameter. The country is a square, consisting of many small block units.

The construction process consists of two steps. First, we generate the population sizes in the block units of the country by drawing a random sample from a reasonable population distribution. In the second step, we apply a clustering algorithm, generating different versions of the country with various levels of clustering. The clustering is done by rearranging the block units according to a mapping rule.

#### Population sizes

The block units are assigned populations which are drawn from a gamma distribution. We use 6561 block units with unit area 81 km^2^, corresponding to a total area of approximately 530 000 km^2^. We rescale our resulting population sizes to sum up to 6 000 000 (slightly larger than Norway). The data are from Statistics Norway for 2016, and we use the 428 municipalities, excluding Svalbard. We use the municipality scale, because this is the finest spatial scale for which we have both population size data and commuting data. The block units can be interpreted as a discretisation of administrative units, so that multiple block units can make up one administrative unit. We do not use the population sizes in the administrative units directly, because a finer scale is necessary for the clustering algorithm to generate smooth distributions. The number of block units is chosen as a trade off between a high resolution, and computational feasibility.

We fit a gamma distribution to the logarithm of the population sizes, since the distribution is clearly very skewed. We draw random realisations from this distribution, providing reasonable population sizes in the block units. The histogram of the population size for the municipalities of Norway and the fitted distribution are given in S1 Fig.

**Fig 1:**
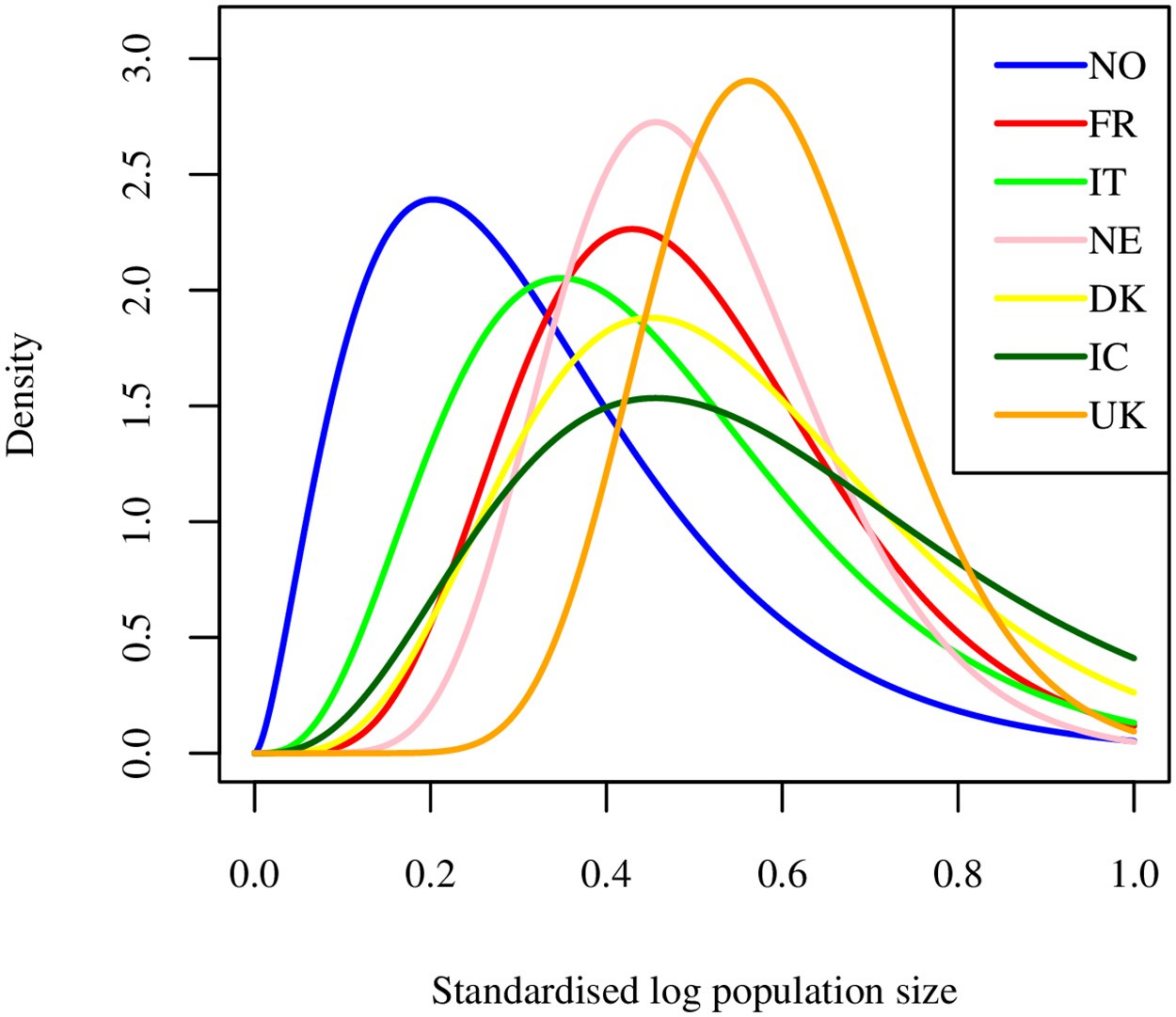
Population sizes. Population size distribution for Norway, France, Italy, Netherlands, Denmark, Iceland and United Kingdom, in different administrative units: 428 municipalities for Norway, 334 arrondissements for France, 107 provinces for Italy, 388 municipalities for the Netherlands, 98 municipalities for Denmark, 129 post codes for Iceland and 424 local authorities for the UK.

We fit distributions to normalised population data from various other countries, to assess similarity across countries. The fitted population distributions are given in Fig 1. For France, we use the 334 arrondissements [40], for Italy the 107 provinces [41], for Netherlands the 388 municipalities [42], for Denmark the 98 municipalities [43], for Iceland the 129 post codes (data for 2017 from Statistics Iceland) and for UK the population sizes in the 424 local authorities (Office for National Statistics GB, estimates for 2016). The population sizes in the Norwegian municipalities seem to be more heterogeneous than for the other countries, with a higher occurrence of smaller population sizes. The UK distribution seems most homogeneous. Noticeably, all the population distributions are skewed. Note that these administrative levels are not standardised across the different countries. Therefore comparison between countries must be treated with caution.

In some of the results, the locations are grouped according to population size. We let Q1 denote areas which have population size smaller than the 25% quantile, Q2 denote areas with population size between the 25% quantile and the median, Q3 denote areas with population size between the median and 75% quantile, and Q4 denote areas with population size larger than the 75% quantile. So Q1 are the most rural areas, while Q4 are the most urban areas in our simulations.

#### Clustering algorithm

The population clustering is done by rearranging the populated locations in space. The population sizes are fixed for all the levels of clustering.

**Fig 2:**
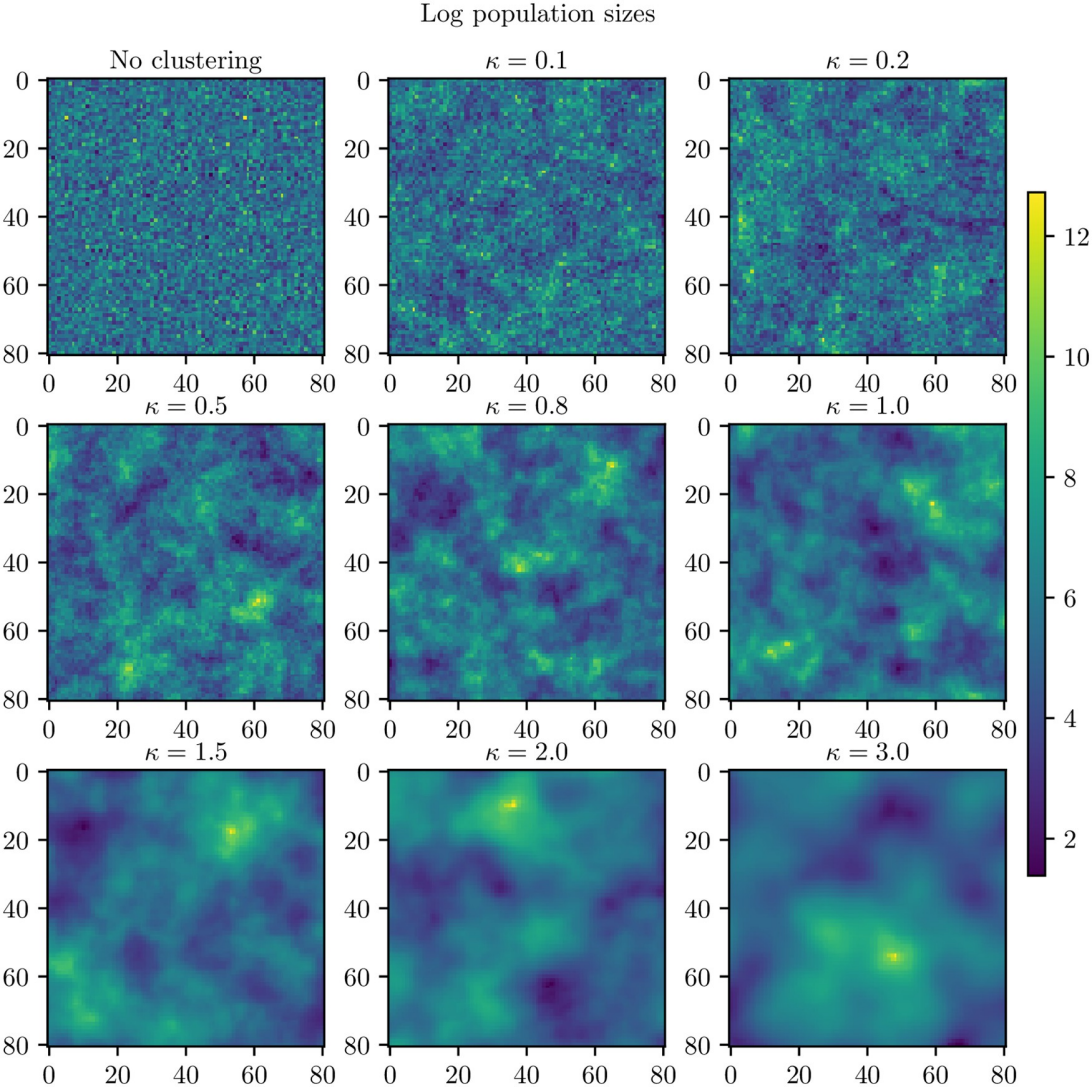
Clustering levels. Generated versions of the country for various *κ*. Upper left: No clustering. Upper center: *κ* = 0.1. Upper right: *κ* = 0.2. Middle left: *κ* = 0.5. Middle center: *κ* = 0.8. Middle right: *κ* = 1.0. Bottom left: *κ* = 1.5. Bottom center: *κ* = 2.0. Bottom right: *κ* = 3.0.

In order to induce positive correlations between the neighbouring locations, we use a geostatistical model to generate a random spatial field where we control the correlations. We use a model with a Matérn covariance function with range parameter 5.0 and process variance 0.1, so that the covariance, *C*, between population sizes in two locations with distance *d* apart is [44, p. 126, section 4.1]

$$\begin{array}{c} C\left(d,\kappa \right)=0.1{\left(2^{\kappa -1}\Gamma \left(\kappa \right)\right)}^{-1}{\left(d/5.0\right)}^{\kappa }K_{\kappa }\left(d/5.0\right), \end{array}$$

where *K*_*κ*_ is a modified Bessel function of the second kind of order *κ* and the parameter *κ* is varied to control the smoothing of population sizes. We use the euclidean distance in block units. The larger *κ*, the stronger the tendency for block units of similar population size to cluster together. This generates a smoother population size distribution in space across the country. We map the sampled population sizes to the random spatial field by respecting the corresponding order of the locations, so that the largest population size is mapped to the location of the largest number in the random spatial field, the second largest population size is mapped to the location of the second largest number in the random spatial field and so on. The resulting versions of the country for various values of *κ* are given in Fig 2. The version in the upper left of Fig 2 is the country without any clustering. We clearly see that population clustering increases with *κ*.

In order to assess what a reasonable range for *κ* is, we perform a visual comparison to some real countries (S2 Fig, S1 Text). Iceland seems to have the highest population clustering level (similar to *κ* = 3.0). Norway and France also seem to have a high population clustering, similar to *κ* = 1.5 or *κ* = 2.0. The United Kingdom seems to be somewhere between *κ* = 1.5 and *κ* = 1.0. Germany seems to be somewhere between *κ* = 0.5 and *κ* = 0.8, while the Netherlands seem similar to something between *κ* = 0.8 and *κ* = 1.0. Similarities are found by visual inspection. A more formal estimation or assessment of *κ* is beyond the scope of this paper. The transformation that we use from the random spatial field to the resulting versions of the countries with different clustering levels is not linear, and it is thus not possible to formally estimate *κ*. However, we are not aiming at providing quantitative results for specific countries, nor is our framework the best choice for that purpose. We are interested in general trends when *κ* is varied, both qualitative and quantitative, in order to be able to make comparisons between different cases. However, the absolute measures (the peak dates, final sizes etc.) are not translatable to specific real settings. When a quantity like the non-infected area during an epidemic changes monotonically when the (synthetic) level of urbanisation increases, then we will hypothesise that urbanisation affects the quantity. When we do not discover a monotone effect, we will conclude that a dependency is less likely.

### The disease dynamics model

The disease dynamics model is a metapopulation model which can be described as a network where every node represents a location, and every edge between locations represents people who travel between the locations and thus can spread the disease further. In every location there is a separate set of stochastic difference equations governing the local disease dynamics, but the processes are coupled through travellers. Similar disease dynamics models are used for instance in [45] and [46] for modelling the global spread of influenza-like illnesses.

#### Local infection dynamics

In every location unit, a stochastic SEIR model [6] is used to describe the infection dynamics. Let *S*^*i*^(*t*), *E*^*i*^(*t*), *I*^*i*^(*t*) and $I_{a}^{i}(t)$ denote the number of susceptible individuals, exposed individuals, symptomatic infectious individuals and asymptomatic infectious individuals at time *t* in location *i*, respectively. As in [45], we let the probability of being asymptomatic (given infectious) be 0.33 and the transmission probability of the asymptomatic infectious be reduced by 50%. The stochastic SEIR equations are given by:

$$\begin{array}{c} S^{i}\left(t+\Delta t\right)=S^{i}\left(t\right)-\text{Binom}(S^{i}\left(t\right),\beta \Delta tI^{i}\left(t\right)/N_{i}+0.5\beta \Delta tI_{a}^{i}\left(t\right)/N_{i}), \end{array}$$

$$\begin{array}{c} E^{i}\left(t+\Delta t\right)=E^{i}\left(t\right)+\text{Binom}(S^{i}\left(t\right),\beta \Delta tI^{i}\left(t\right)/N_{i}+0.5\beta \Delta tI_{a}^{i}\left(t\right)/N_{i})\\ -\text{Multinom}(E^{i}\left(t\right),0.33\lambda \Delta t,0.67\lambda \Delta t), \end{array}$$

$$\begin{array}{c} I^{i}\left(t+\Delta t\right)=I^{i}\left(t\right)+\text{Binom}(E^{i}\left(t\right),0.67\lambda \Delta t)-\text{Binom}(I^{i}\left(t\right),\gamma \Delta t), \end{array}$$

$$\begin{array}{c} I_{a}^{i}\left(t+\Delta t\right)=I_{a}^{i}\left(t\right)+\text{Binom}(E^{i}\left(t\right),0.33\lambda \Delta t)-\text{Binom}(I_{a}^{i}\left(t\right),\gamma \Delta t), \end{array}$$

where *β* is the transmission probability per unit time, *N*_*i*_ is the population in location *i*, 1/λ is the average latent period, 1/*γ* is the average infectious period, Binom(*n*, *p*) is the binomial distribution with *n* trials and success probability *p* and Multinom(*n*, *p*_1_, *p*_2_) is the multinomial distribution with *n* trials and success probabilities *p*_1_ and *p*_2_. The equation for *R*^*i*^(*t*) (the number of recovered/removed individuals in location *i* at time *t*) is redundant, since we assume that the total population size remains constant during the epidemic. Using this model, the estimated basic reproductive number (the average number of new cases caused by an infectious individual in a fully susceptible population) is given by $R_{0}=\frac{\beta }{\gamma }(0.5\cdot 0.33+0.67)$ [46]. We choose a time step Δ*t* of 12 hours, in order to distinguish day time from night time.

#### Mobility and global infection dynamics

In the model, the disease dynamics in the block units are coupled through travelling individuals. Every individual has a defined home and work location. During day time, the individuals mix at their work location, while at night, they mix at their home location. We stress the importance of keeping track of the commuting individuals, that is, making sure the same individuals are commuting every day. Keeling et al. [47] find that by keeping track of the individuals who regularly commute, the spread rate of the epidemics is substantially reduced.

Commuting is implemented by a gravity law [48]. The parameters of the gravity law are fitted to data from Statistics Norway [49] on commuting between Norwegian municipalities.

The fitted gravity model is

$$\begin{array}{c} w_{ij}\propto \frac{N_{i}^{0.73}N_{j}^{0.51}}{d_{ij}^{1.22}}, \end{array}$$

where *w*_*ij*_ is the number of commuters from location *i* to location *j*, *N*_*i*_ is the population size in location *i*, *N*_*j*_ is the population size in location *j* and *d*_*ij*_ is the distance (in meters) between the locations *i* and *j*. The resulting number of commuters are scaled in order to approximately match the proportion of commuters in Norway, which is 0.177. The scaling is approximate, since the number of commuters between any two pairs of locations has to be an integer number, so we round down to the nearest integer.

In addition to commuting, we consider non-commuting travel. Non-commuting travel is implemented in the model by allowing all the non-commuting individuals to travel to a random location, with some fixed probability. If the individual travels, the destination location is random, with probabilities proportional to the population size in the locations. The non-commuting individuals who travel, mix in the destination population for one day and one night (24 hours), before returning to their home location. The number of people in location *i* during day time, $N_{i}^{\text{day},t}$, and night time, $N_{i}^{\text{night},t}$, are given by

$$\begin{array}{c} N_{i}^{\text{day},t}=N_{i}^{\text{home}}+\sum_{j}w_{j,i}+\sum_{j}u_{j,i}^{t}-\sum_{j}u_{i,j}^{t}, \end{array}$$

$$\begin{array}{c} N_{i}^{\text{night},t}=N_{i}^{\text{home}}+\sum_{j}w_{i,j}+\sum_{j}u_{j,i}^{t}-\sum_{j}u_{i,j}^{t}, \end{array}$$

where $N_{i}^{\text{home}}$ is the number of people living in location *i* who do not commute, $u_{i,j}^{t}$ is the number of non-commuters travelling from location *i* to location *j* on day *t*, and the *t* indicates that the number of people varies from day to day.

The travel probability is set to control the ratio of non-commuting to commuting. We denote this ratio by *τ*. A ratio of non-commuting travel to commuting of 1/10 is likely to be the most plausible travel ratio, in the baseline scenario with no travel restrictions. Hence, *τ* = 1/10 is the baseline scenario. By using the number of yearly domestic flights in Norway as a proxy for non-commuting travel, we find a ratio of non-commuting travel to commuting of approximately 1/12. This is in agreement with [45], where they state that commuting flows are one order of magnitude larger than airline flows.

#### Simulation set-up and seeding

The process is stochastic, and we perform 100 disease simulations for each clustering level and report the average. The epidemic is dynamically seeded by placing one infectious individual in ten different locations with large population size (above the 80% quantile), and two infectious individuals in the location with largest population size, every day. This corresponds to 12 seeding events daily, which is 0.04% of the number of arriving international air travel passengers in Norway in 2016, as in [15]. The seeding locations have the same population sizes for all the different clustering levels. The disease parameters are set to mimic an influenza with high transmissibility, since implementations of interventions are more relevant for influenza strains with large transmission potential. The average latent period is set to 1.9 days, the average infectious period is set to 3.0 days (as in [45]) and the transmission parameter is set to 0.60, corresponding to a basic reproductive number of 1.50.

### Interventions

The following sections describe the two intervention measures examined in this work. They are applied both in separate simulations to isolate their effects, and in a combination strategy.

#### Travel restrictions

Travel restrictions are varied through the amount of non-commuting travel in the simulations, for the various levels of population clustering. The scenarios we consider are *τ* = 0 (only commuting), *τ* = 1/1000, *τ* = 1/100 and *τ* = 1/10 (baseline scenario).

#### Vaccination

We simulate the epidemic with three different vaccination strategies—vaccinating uniformly in space, preferentially vaccinating rural locations and preferentially vaccinating urban locations.

We assume that we have enough vaccines to vaccinate 40% of the population, similar to the vaccine coverage in Norway during the 2009 pandemic [25]. We let the individuals eligible for the vaccine be the susceptible individuals. We assume that the vaccine efficacy is 70%, so that 70% of the vaccinated individuals become immune to the virus, while the remaining 30% remain susceptible, in line with estimates of the efficacy of the 2009 pandemic vaccine [50]. Susceptible individuals who are vaccinated but still susceptible, are likely to experience fewer symptoms and be more resistant to the virus if they become infected, so we assume that they are 20% less infectious (in S1 Text, we also analyse the more optimistic setting where they are 80% less infectious). We assume that the vaccine is introduced 75 days after the first influenza case introduction, and that the vaccines are distributed uniformly (in time) each day for six weeks.

In the setting with uniform vaccination, we vaccinate (approximately) 40% of the population in each location. The approximation is due to the integer approximations, so we vaccinate to the nearest integer. Since we do not vaccinate immediately, there might be locations where the number of susceptibles is too small to vaccinate 40%. The remaining vaccination doses are then uniformly distributed in the other locations. In the urban vaccination strategy, we vaccinate preferentially in urban locations, by only vaccinating the 50% largest locations, using the same total number of vaccines as in the uniform vaccination setting. In the rural vaccination strategy, we vaccinate preferentially in rural locations, by leaving out the 2% locations with highest population size, using the same total number of vaccines as in the uniform vaccination setting.

### Model outcomes

The epidemics are compared by examination of final size, peak date, peak prevalence and the proportion of area that is not infected during the epidemic. The final size is defined as the total number who were infected during the epidemic. The peak date is the date with the highest number infected, and the peak prevalence is the proportion infected on the peak date. The area not infected is the proportion of block units where the prevalence was never larger than a threshold for seven consecutive days. The threshold is 1.0%, except from in the locations where the population size is less than 100, then the threshold is one case.

We compare quantitative properties to compare the effect sizes between different clustering levels in order to assess whether or not the clustering plays an important role. We are thus interested in whether there is a monotone relationship between such a quantity and *κ*, while the specific values are less interesting.

## Results

### Baseline scenario

**Fig 3:**
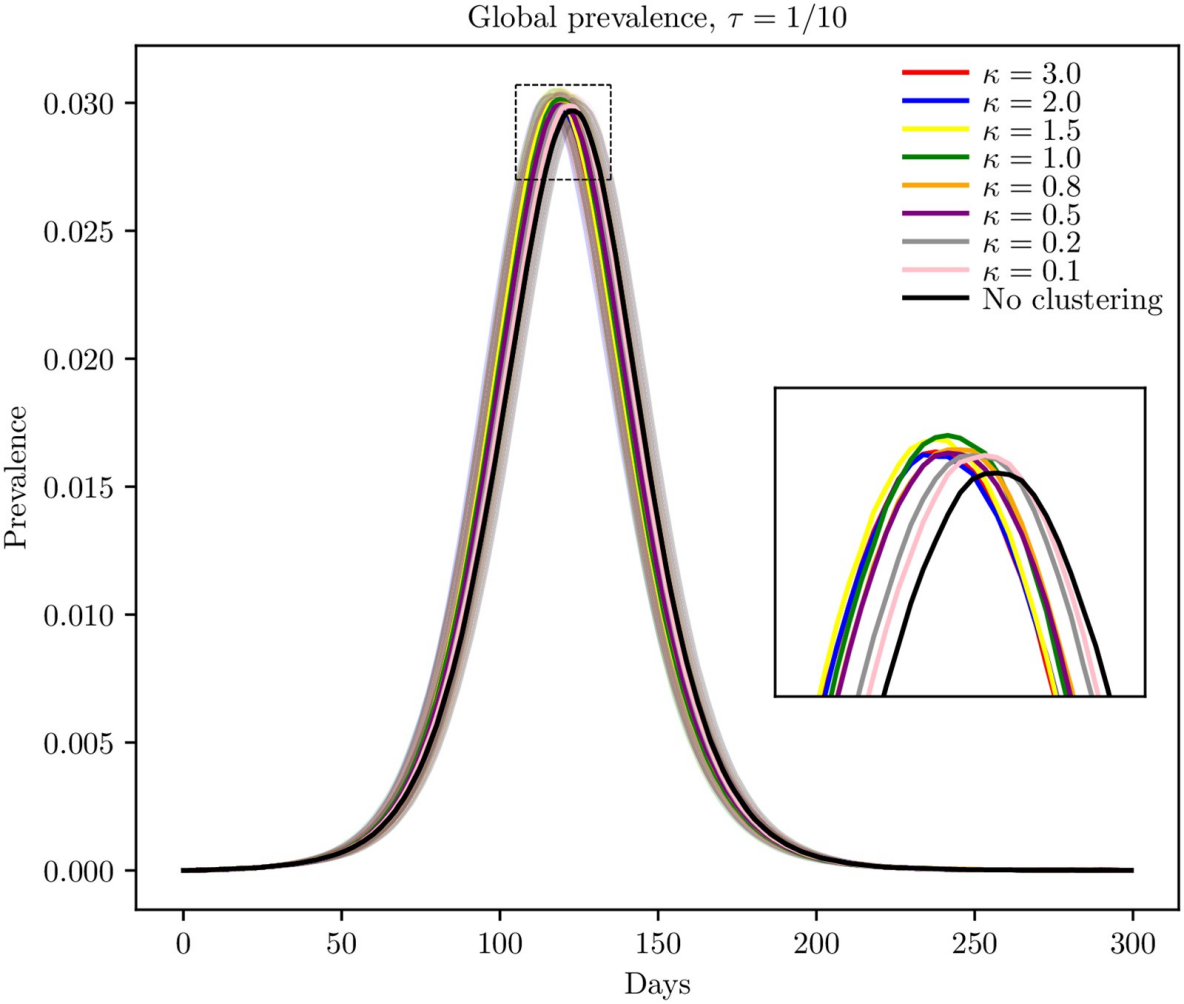
Global prevalence in baseline scenario. Estimated global prevalence for the various clustering levels in the baseline scenario with no interventions, with 95% confidence bands around the mean.

**Fig 4:**
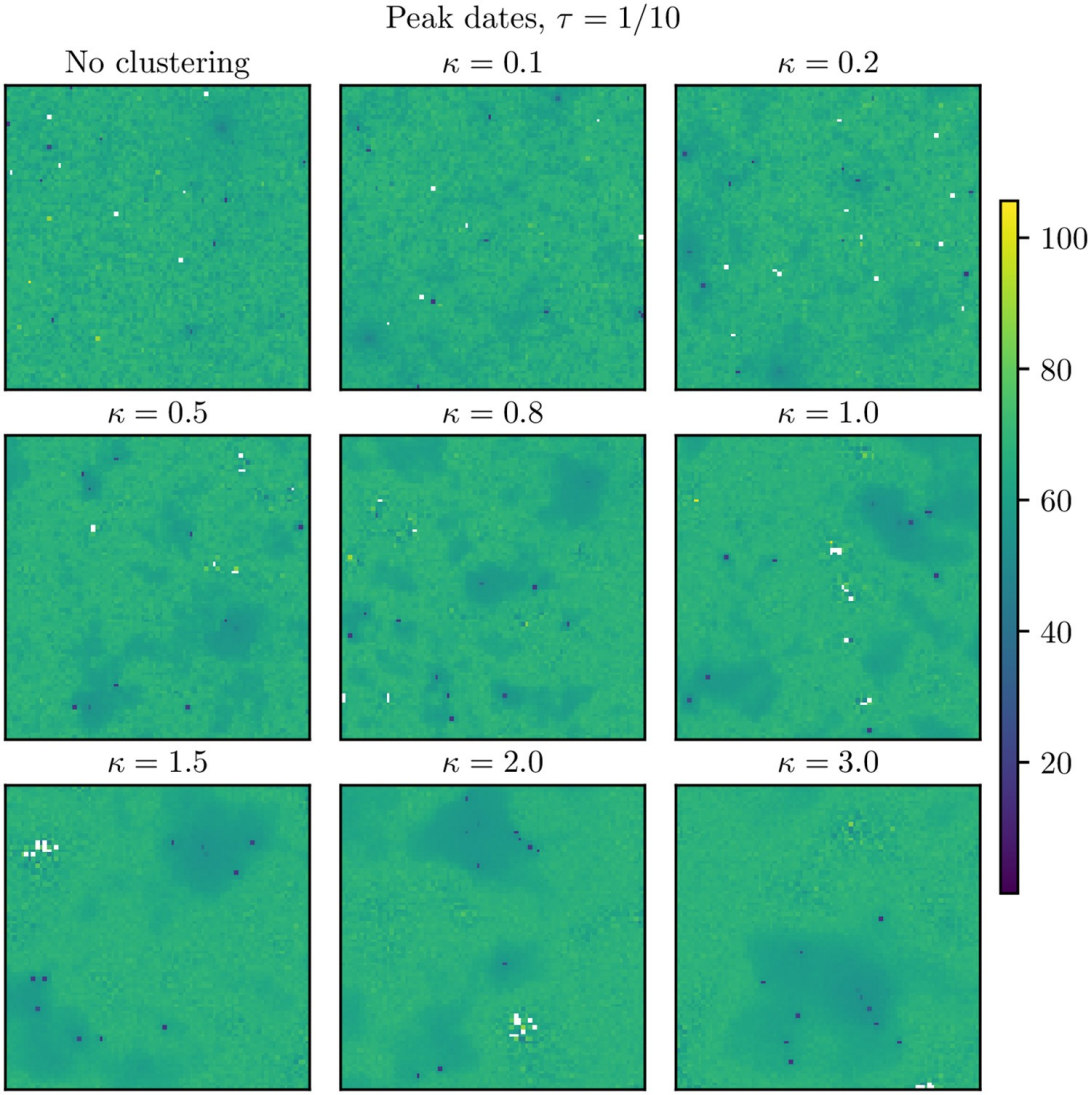
Peak dates for baseline scenario. Peak dates for the various clustering levels. These are averages over the simulations where an epidemic occurred in the respective block units. The white locations never experienced the epidemic. Upper left: No clustering. Upper center: *κ* = 0.1. Upper right: *κ* = 0.2. Middle left: *κ* = 0.5. Middle center: *κ* = 0.8. Middle right: *κ* = 1.0. Bottom left: *κ* = 1.5. Bottom center: *κ* = 2.0. Bottom right: *κ* = 3.0. The seeding locations are seen as blue dots.

In order to compare the dynamics for the different levels of population clustering, the disease spread was simulated for the various clustering levels. It is intuitive that with a large enough amount of non-commuting travel, there will be sufficient mixing between all the block units in the country for the population structure not to play a role in the disease dynamics. The mean global prevalence curves for the different clustering levels are given in Fig 3 for the baseline scenario with no vaccination and no travel restrictions. The curves were visually very similar, but the higher clustering levels tended to have a higher and earlier peak. Note that the confidence bands were overlapping for most clustering levels. This indicates that with the baseline amount of non-commuting, the mixing was so high that the underlying population clustering seemed to have little effect on the disease dynamics. Slightly less area was infected for the higher clustering levels, and the final sizes were slightly smaller the more clustering (Table 1). The peak dates (the day with the largest number of infected symptomatic individuals) in the different block units are given in Fig 4. The epidemic was able to spread throughout the whole country. We found spatial clustering in peak dates (by visual inspection), and the spatial clustering was larger the more clustered the country in terms of population size.

**Table 1:** Baseline scenario.

*κ*	Area not infected	Final size
No clustering	0.187 (0.00392)	0.575 (0.000791)
0.1	0.193 (0.00324)	0.574 (0.000837)
0.2	0.200 (0.00323)	0.574 (0.000811)
0.5	0.209 (0.00401)	0.573 (0.000895)
0.8	0.209 (0.00399)	0.573 (0.000815)
1.0	0.211 (0.00435)	0.572 (0.000880)
1.5	0.213 (0.000379)	0.572 (0.000932)
2.0	0.214 (0.00388)	0.572 (0.000903)
3.0	0.214 (0.00400)	0.572 (0.000845)

Percentage of non-infected area and final sizes, for different levels of clustering. Standard deviations are given in parenthesis.

### Travel restrictions

Three scenarios were considered: *τ* = 0 (only commuting), *τ* = 1/100 (90% travel restrictions) and *τ* = 1/1000 (99% travel restrictions).

Table 2 shows the mean percentage of area not infected and the mean final size for *τ* = 0. The final sizes of the epidemic decreased with increased clustering, and the area which did not experience the infection increased with increased clustering. The final size for the country with no clustering was 14% higher than for the most clustered (*κ* = 3.0).

**Table 2:** *τ* = 0.

*κ*	Area not infected	Final size
No clustering	0.511 (0.00418)	0.525 (0.00138)
0.1	0.542 (0.00455)	0.519 (0.00159)
0.2	0.606 (0.00428)	0.501 (0.00228)
0.5	0.694 (0.00427)	0.480 (0.00267)
0.8	0.700 (0.00473)	0.479 (0.00291)
1.0	0.696 (0.00387)	0.489 (0.00195)
1.5	0.777 (0.00361)	0.458 (0.00310)
2.0	0.802 (0.00391)	0.440 (0.00335)
3.0	0.794 (0.00304)	0.454 (0.00185)

Percentage of area not infected and final sizes. Standard deviations are given in parenthesis.

**Fig 5:**
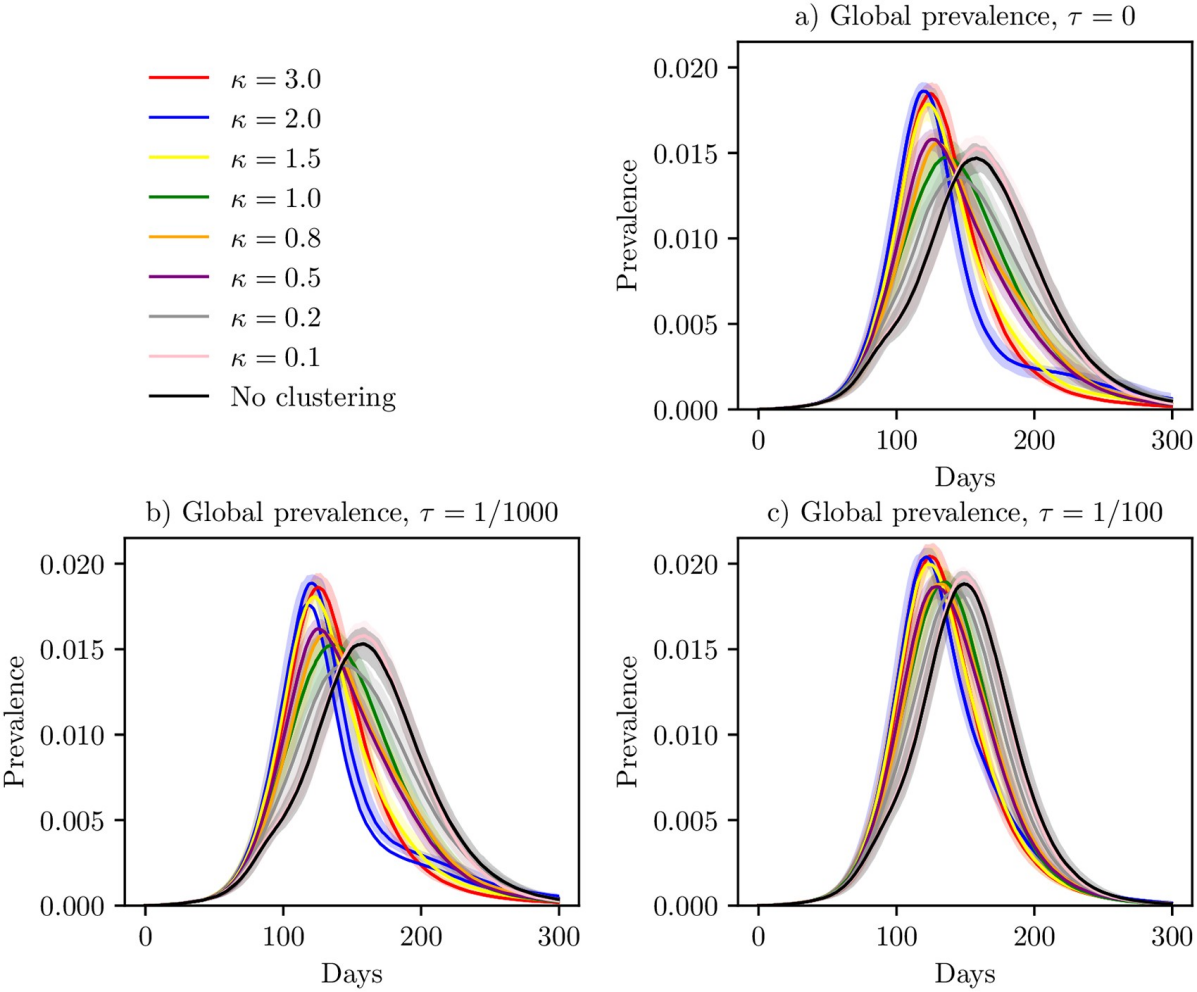
Global prevalence under travel restrictions. Global prevalence curves in the situation with travel restrictions included in the model, with *τ* = 0 (a), *τ* = 1/1000 (b), *τ* = 1/100 (c), with 95% confidence bands around the mean.

The global prevalence curves for the different clustering levels for *τ* = 0 are given in Fig 5a. The higher clustering levels experienced an earlier peak and a higher peak prevalence. Considering the confidence bands, these curves are significantly different—not between every clustering level, but the prevalence curves for the higher clustering levels are significantly different from the prevalence curves for the lower clustering levels. Interestingly, the higher peak prevalence did not imply a larger final size, as could be expected. Instead, the higher clustering levels had both higher peak prevalences and lower final sizes.

**Fig 6:**
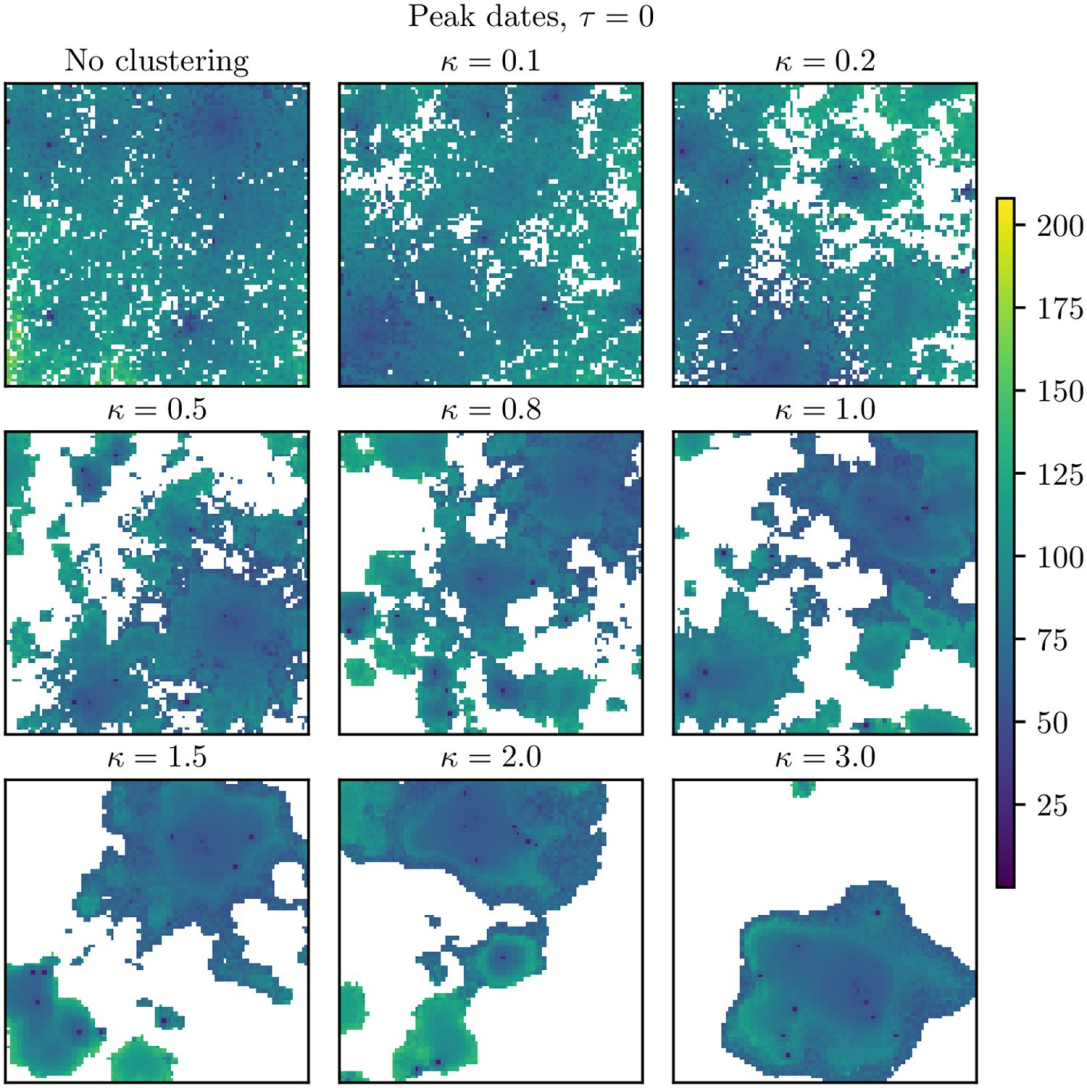
Peak dates for *τ* = 0. Peak dates for infection when *τ* = 0. These are averages over the simulations where an epidemic occurred in the respective block units. The white locations never experienced the epidemic. Upper left: No clustering. Upper center: *κ* = 0.1. Upper right: *κ* = 0.2. Middle left: *κ* = 0.5. Middle center: *κ* = 0.8. Middle right: *κ* = 1.0. Bottom left: *κ* = 1.5. Bottom center: *κ* = 2.0. Bottom right: *κ* = 3.0.

In Fig 6, we plotted the peak dates for *τ* = 0. In addition, the initial date, peak prevalence and the probability of experiencing the epidemic in each location are given in S3, S4 and S5 Figs. There was spatial clustering in both the initial dates, peak dates, peak incidences and probabilities of experiencing the epidemic, and the spatial clustering increased with increased (population) clustering levels. The more clustered the country, the fewer locations were infected on average (Table 2). In the peak dates plot in Fig 6, we see that we had some locations which were not infected in any of the 100 simulations (coloured white). For the higher *κ*, these non-infected locations were clustered, and the cluster sizes increased with *κ*. The epidemic did not spread throughout the whole country for the highest clustering levels, but seemed to be restricted to the highly populated area.

The mean global prevalence curves for the settings with *τ* = 1/1000 and *τ* = 1/100 are given in Fig 5b and 5c, respectively. In the prevalence curves for *τ* = 1/1000, we got similar results as for *τ* = 0, with an earlier and sharper peak for the higher clustering levels. The same was found for *τ* = 1/100, but the differences between the curves were smaller, as expected.

For *τ* = 1/1000, fewer locations were infected and the final size decreased with increased clustering, just as in the setting with *τ* = 0 (cf. Table 3). The same was found for *τ* = 1/100 in Table 4, but the differences were smaller. The effect of travel restrictions on reducing the final size was larger with higher clustering. The *τ* = 0, *κ* = 3.0 scenario had 21% reduced final size, while the *τ* = 0, no clustering-scenario had a 9% reduction. For the 99% travel restrictions, the reductions were 19% for *κ* = 3.0 and 8% for the scenario without clustering. For the 90% travel restrictions, the corresponding reductions were 12% and 6%.

**Table 3:** *τ* = 1/1000.

*κ*	Area not infected	Final size
No clustering	0.501 (0.00441)	0.527 (0.00130)
0.1	0.532 (0.00397)	0.522 (0.00155)
0.2	0.591 (0.00416)	0.507 (0.00182)
0.5	0.674 (0.00389)	0.489 (0.00212)
0.8	0.679 (0.00411)	0.488 (0.00213)
1.0	0.679 (0.00324)	0.494 (0.00159)
1.5	0.753 (0.00372)	0.467 (0.00193)
2.0	0.772 (0.00389)	0.455 (0.00248)
3.0	0.772 (0.00307)	0.461 (0.00173)

Percentage of non-infected area and final sizes, for different levels of clustering. Standard deviations are given in parenthesis.

**Table 4:** *τ* = 1/100.

*κ*	Area not infected	Final size
No clustering	0.430 (0.00406)	0.543 (0.00107)
0.1	0.454 (0.00425)	0.540 (0.00118)
0.2	0.497 (0.00459)	0.532 (0.00140)
0.5	0.556 (0.00451)	0.522 (0.00131)
0.8	0.559 (0.00443)	0.521 (0.00156)
1.0	0.566 (0.00486)	0.522 (0.00123)
1.5	0.610 (0.00438)	0.508 (0.00161)
2.0	0.618 (0.00468)	0.506 (0.00153)
3.0	0.625 (0.00414)	0.504 (0.00150)

Percentage of non-infected area and final sizes, for different levels of clustering. Standard deviations are given in parenthesis.

**Fig 7:**
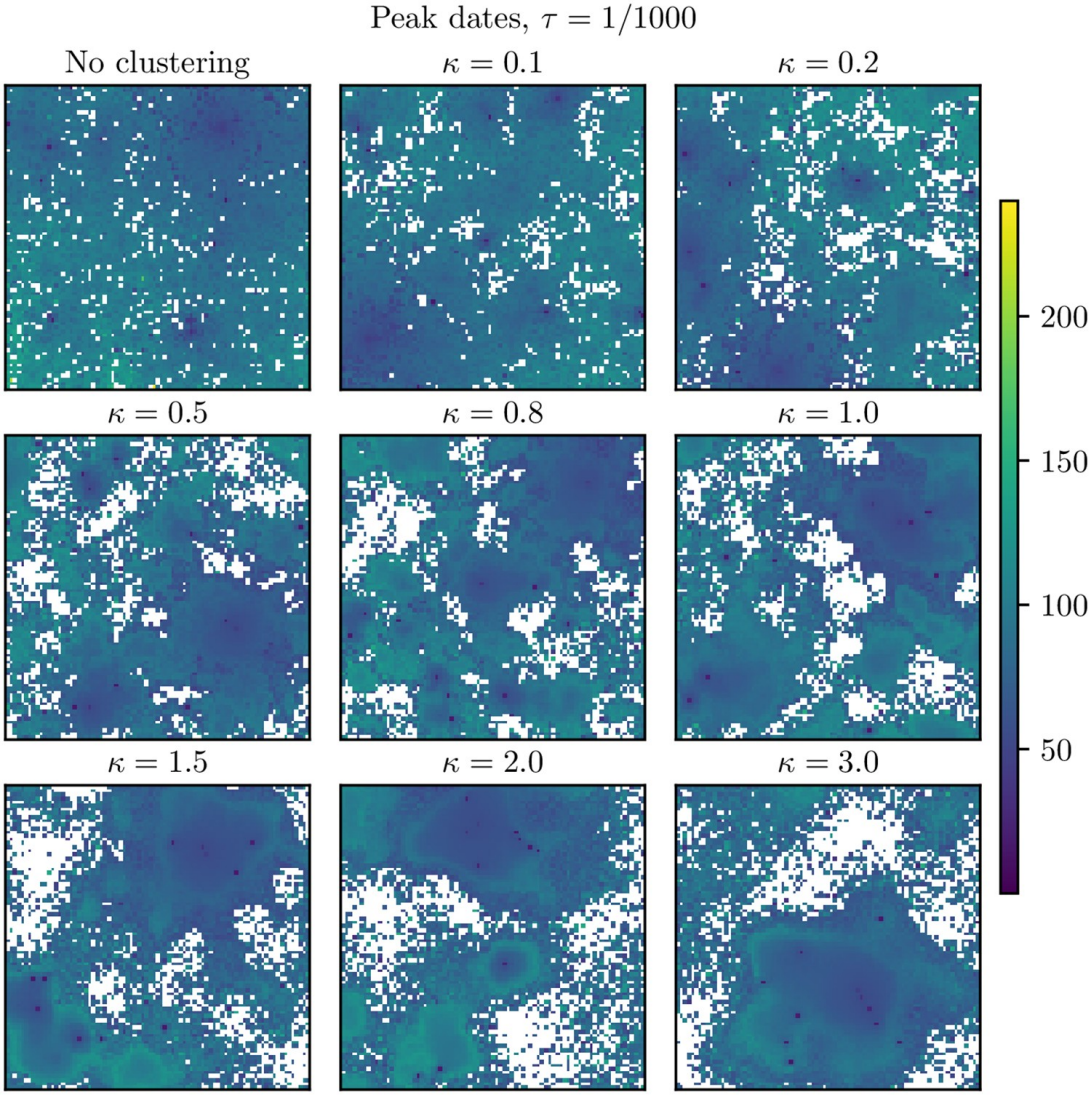
*τ* = 1/1000: Peak dates. Peak dates in the setting with *τ* = 1/1000 for the various clustering levels. These are averages over the simulations where an epidemic occurred in the respective block units. The white locations never experienced the epidemic. Upper left: No clustering. Upper center: *κ* = 0.1. Upper right: *κ* = 0.2. Middle left: *κ* = 0.5. Middle center: *κ* = 0.8. Middle right: *κ* = 1.0. Bottom left: *κ* = 1.5. Bottom center: *κ* = 2.0. Bottom right: *κ* = 3.0.

**Fig 8:**
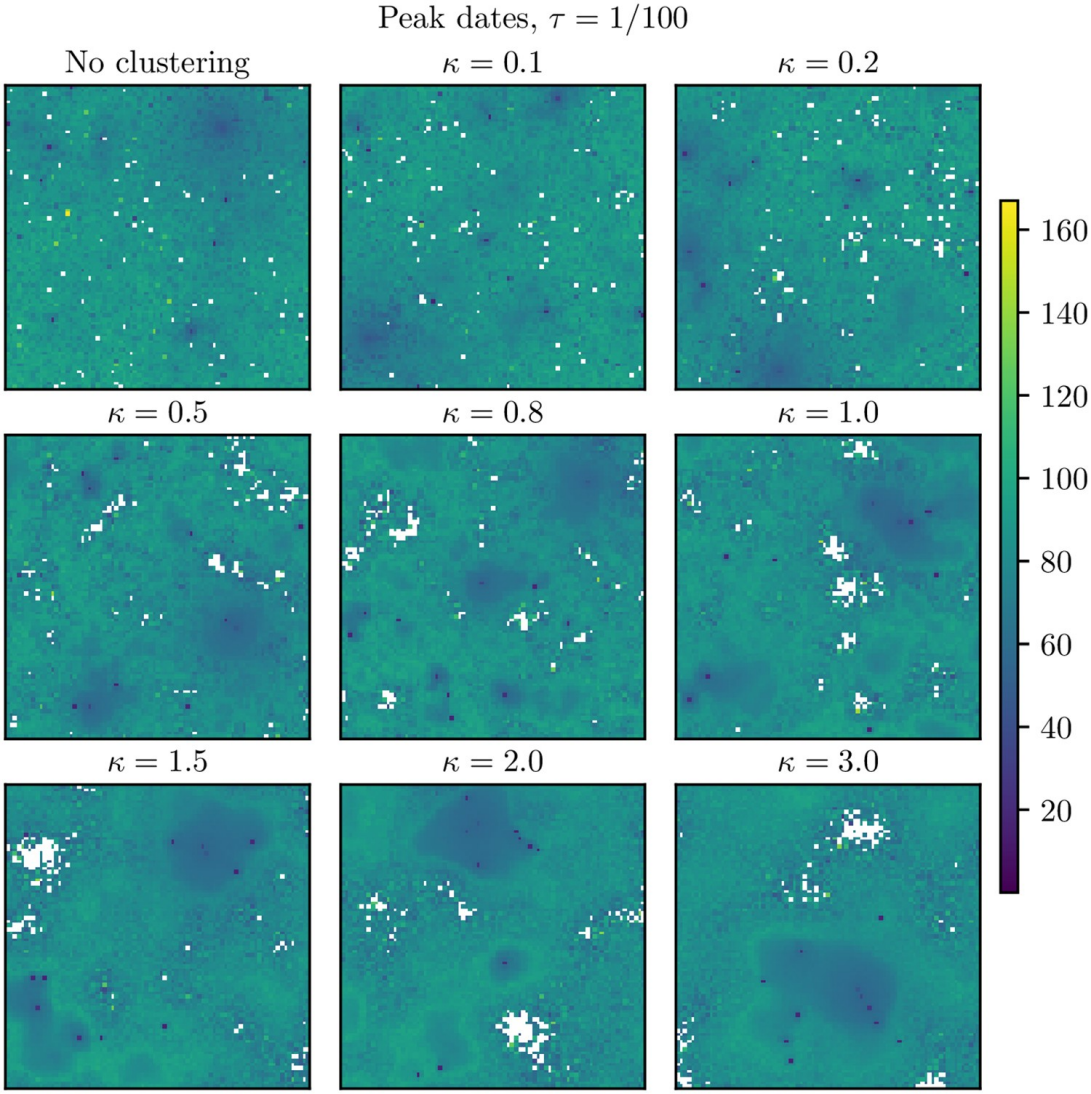
*τ* = 1/100: Peak dates. Peak dates in the setting with *τ* = 1/100 for the various clustering levels. These are averages over the simulations where an epidemic occurred in the respective block units. The locations which were never infected are coloured in white. Upper left: No clustering. Upper center: *κ* = 0.1. Upper right: *κ* = 0.2. Middle left: *κ* = 0.5. Middle center: *κ* = 0.8. Middle right: *κ* = 1.0. Bottom left: *κ* = 1.5. Bottom center: *κ* = 2.0. Bottom right: *κ* = 3.0.

The peak dates for *τ* = 1/1000 and *τ* = 1/100 are given in Figs 7 and 8, respectively. Fig 7 shows that for *τ* = 1/1000, there were some clusters of protected locations which did not experience the infection in any of the simulations. Comparing with the situation with *τ* = 0 in Fig 6, we found that when adding the non-commuting travel, the infection was no longer trapped in the larger hubs for the higher levels of clustering, but was able to infect a larger area of the country. There were also some protected clusters of locations with *τ* = 1/100 (as opposed to the baseline scenario).

**Fig 9:**
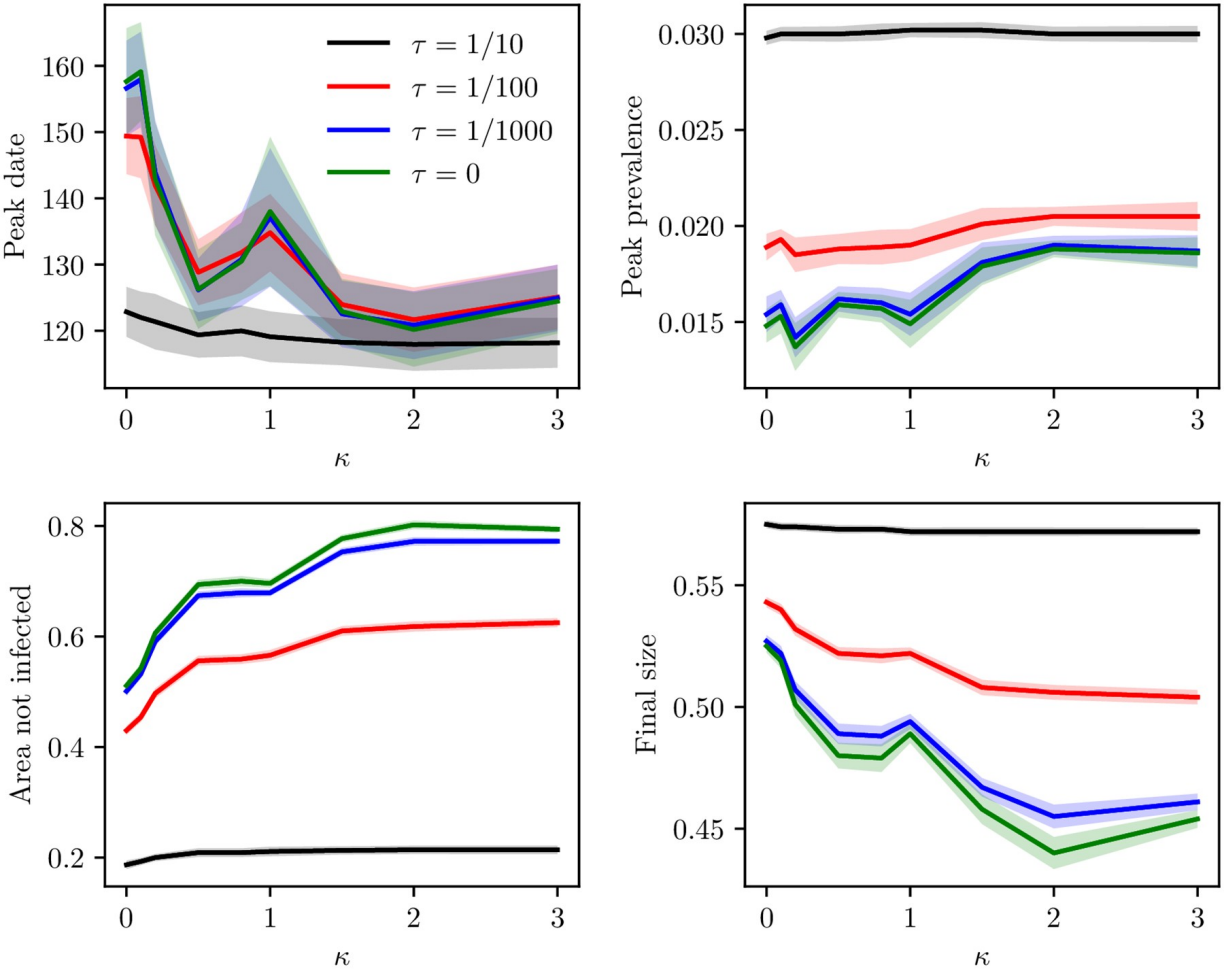
Peak date, peak prevalence, area not infected and final size. Peak dates for the global prevalence curve, peak prevalence, mean area not infected and mean final size as a function of clustering, with corresponding 95% confidence bands. The lines correspond to the baseline scenario, 90% travel restrictions, 99% travel restrictions and 100% travel restrictions. Top left: peak date. Top right: peak prevalence. Bottom left: area not infected. Bottom right: final size.

We have plotted the peak date for the mean global prevalence curve, peak prevalence, mean area not infected and mean final size for the various levels of clustering, for the baseline scenario and the travel restriction scenarios (Fig 9). The peak dates occurred earlier for increased levels of clustering, for all the travel ratios. In addition, the curves were very similar for the three travel restriction scenarios, while the peak dates occurred earlier for the baseline scenario. Hence, implementing travel restrictions delayed the epidemic peak. For the peak prevalence, we note that the higher levels of travel restrictions, the lower the peak. The decrease in peak prevalence was larger for the lower clustering levels. The decrease in peak prevalence for 99% travel restrictions compared to the baseline scenario was 38% for the highest clustering level and 48% for the lowest clustering level. There was almost no difference between the 99% travel ban scenario and the 100% travel ban scenario. The peak prevalence increased with increased clustering. The difference in peak prevalence with clustering was more prominent the more extensive the travel restrictions. The peak prevalence for *κ* = 3.0 was 20% higher than the peak prevalence for the “no clustering”-level for the complete travel ban scenario. For the mean area not infected, there was little difference between the 99% travel restrictions and the full travel ban setting. The more travel restrictions, the more area was protected. In addition, the amount of area which was protected increased with increased clustering, and the effect of clustering was stronger the more travel restrictions. For the final sizes, we found that the more travel restrictions, the lower the final size. In addition, as we have seen, for the travel restriction scenarios, the final size was lower for higher clustering levels. The more extensive the travel restrictions, the larger the difference between the various clustering levels.

#### Final size versus travel ratio

**Fig 10:**
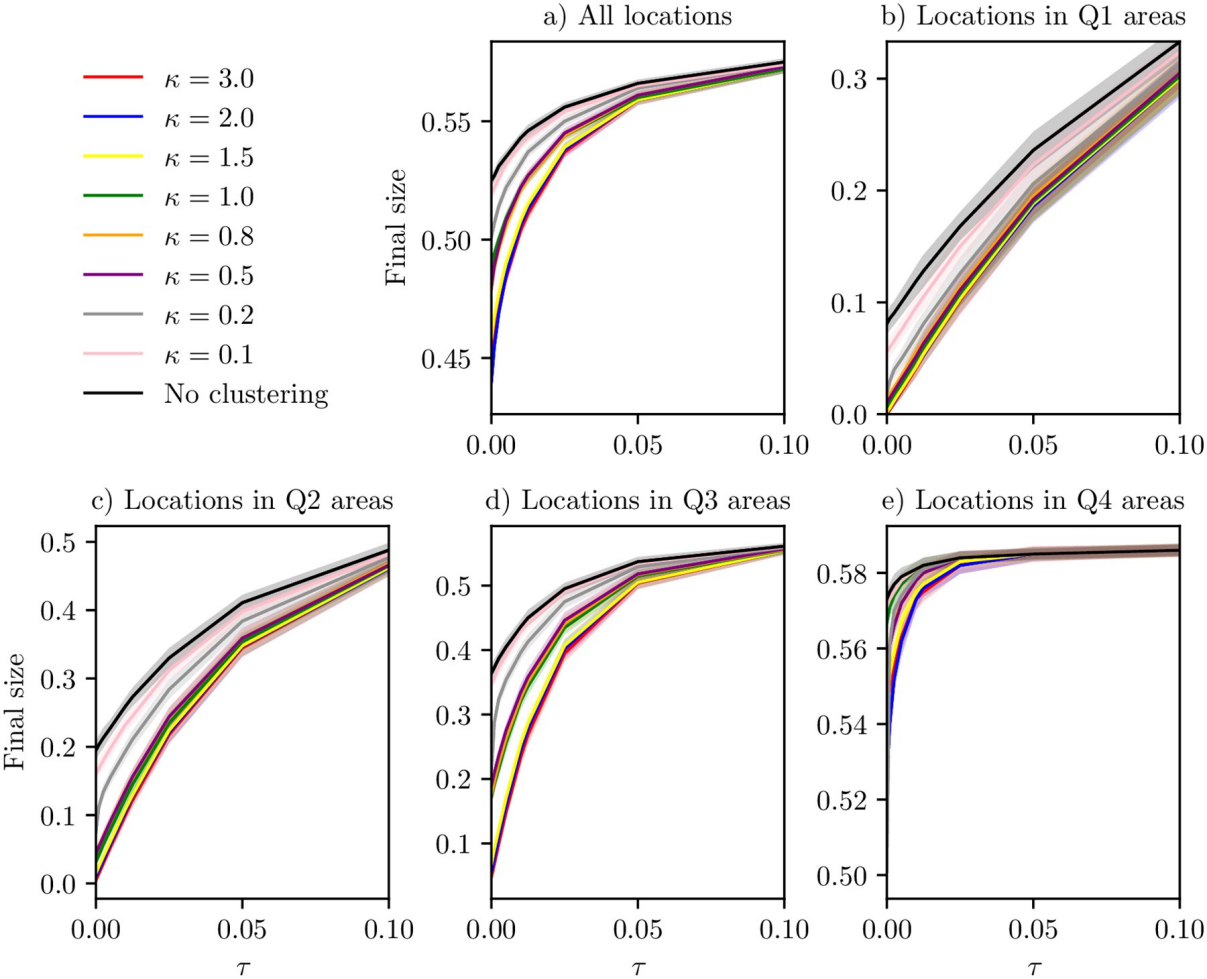
Final size versus travel ratio. Final size versus ratio of non-commuting travel to commuting for various clustering levels, *κ*, with corresponding 95% confidence bands. a) All locations. b) Locations with population size smaller than the 25% quantile. c) Locations with population size between the 25% and 50% quantile. d) Locations with population size between the 50% quantile and the 75% quantile. e) Locations with population size larger than the 75% quantile.

To further investigate the relationship between final size and travel ratio for the various clustering levels, we also considered *τ* = 1/400, *τ* = 1/200, *τ* = 1/80, *τ* = 1/40 and *τ* = 1/20, in addition to *τ* = 0 (only commuting), *τ* = 1/1000, *τ* = 1/100 and *τ* = 1/10. The final size of the epidemic versus travel ratio for the different levels of clustering are given in Fig 10a. For all the clustering levels, the final size increased with increased travel ratio, as expected, and the confidence bands are insignificant compared to the variation over the *τ* range. We found a much larger increase in final size when increasing the amount of non-commuting travel for the higher levels of clustering, than for the lower clustering levels. Hence there was a rapid increase in final size for the highest clustering levels with increased amounts of non-commuting travel. Further inspecting the final size in the various locations, we found that the decrease in final size with travel restrictions was mainly due to fewer locations being infected, but there was also some decrease in final size beyond this effect. In particular, comparing the final size in the locations which are hit in every *τ*-scenario, the difference in final size is largest for *κ* = 2.0, where the average final size decreases from 0.584 (*τ* = 1/10) to 0.575 (*τ* = 0). The smallest difference is for *κ* = 0, where the average final size is slightly larger for *τ* = 0 than for *τ* = 1/10 (0.583 versus 0.582). For other *κ* values, there was a decrease, however, the decrease was much smaller than the total decrease in final size, due to fewer locations being hit.

From the plots of the peak dates for the various travel ratios, there seemed to be some protected clusters in the settings with a low *τ*, where the epidemic seemed to be restricted to the hubs. We therefore expect the epidemic risk to be different for locations with different population sizes. We plotted the mean final size versus travel ratio, for various levels of *κ*, in areas of different population size. The final sizes for the Q1, Q2, Q3 and Q4 areas are given in Fig 10b–10e. For the Q1, Q2 and Q3 areas, we found that the higher levels of clustering were more affected by the travel restrictions. The final size in these areas increased more with increasing *τ* for large *κ*. This coincides with high population clustering having protected clusters for lower values of *τ*. For the Q4 areas, we also found a more rapid increase in final size for the higher clustering levels, but the curve quickly levels off to a point where a further increase in *τ* does not affect the final size much. In S1 Text, we fit functions for final size versus *τ* for all the clustering levels, and find a monotone and positive relationship between growth rate and *κ* for the Q1, Q2 and Q3 locations.

#### Peak date versus travel ratio

**Fig 11:**
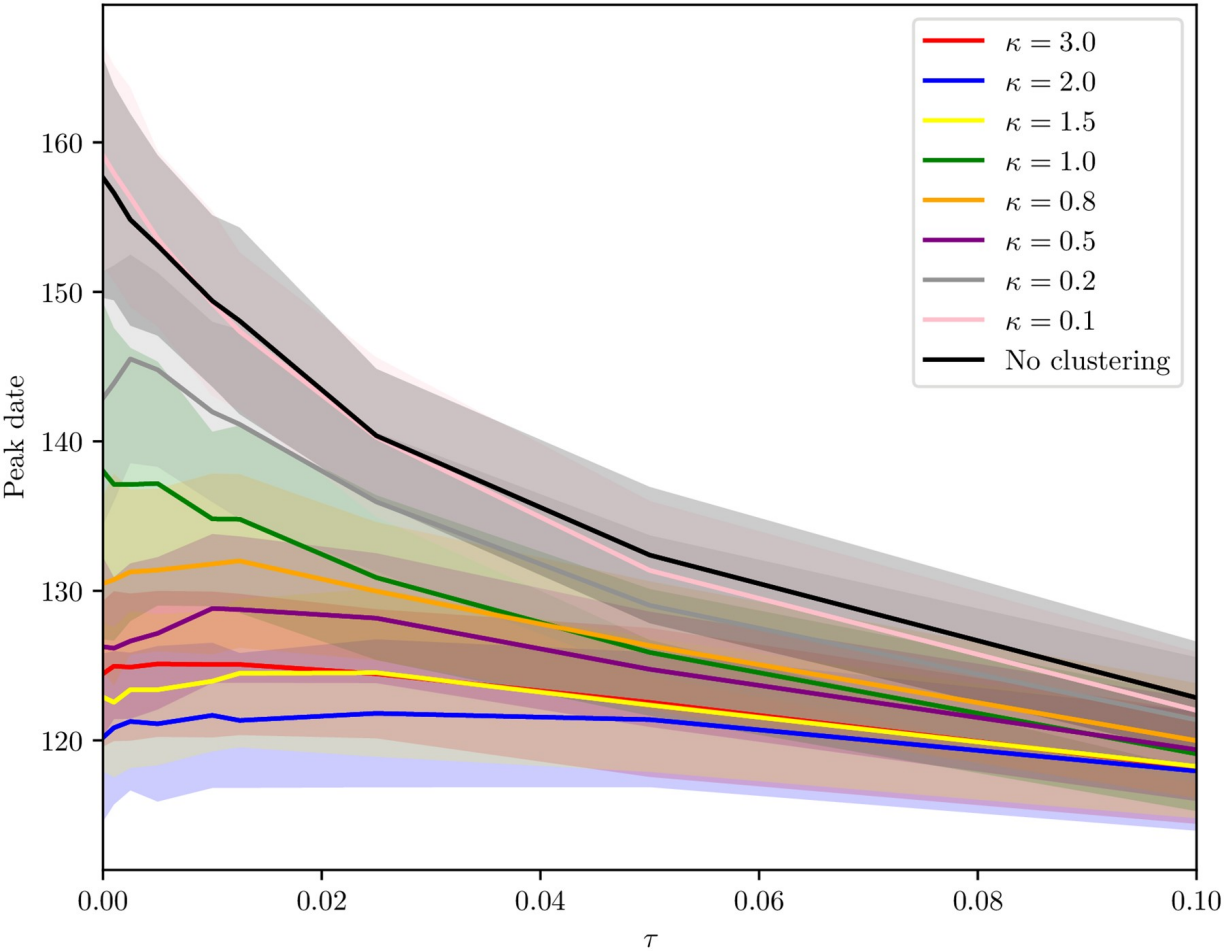
Peak date versus travel ratio. Peak date versus ratio of non-commuting travel to commuting for various clustering levels, *κ*, with 95% confidence bands.

Overall, the epidemic peaked earlier with higher levels of clustering. We plotted the peak date versus travel ratio, for the different *κ*. The plot is given in Fig 11. The difference in peak date between the clustering levels decreased with increased non-commuting travel, as expected, and there was a rapid decrease for the low clustering levels. Travel restrictions delayed the epidemic with up to one week for the highest clustering levels and up to five weeks for the lowest clustering levels.

#### Sensitivity analysis

We performed sensitivity analysis with respect to delay in implementation of travel restrictions, the length of stay for the non-commuter travellers, the disease parameters and the country the population data is based on (population size distribution and gravity law). We performed sensitivity analysis with respect to the parameters in the gravity law and the shape of the gravity law. We performed the analysis using the radiation law [51] to model commuting, instead of the gravity law, and an analysis with an increased range parameter of the Matérn covariance function. In addition, we performed simulations in the setting where only the symptomatic infectious individuals were restricted from travelling. In general, the qualitative results for the various sensitivity analyses were similar to the results in the main analysis, and the details are provided in S1 Text. The effect sizes differ between the various settings. We will also comment on the robustness of the different qualitative results to these settings in the Discussion section.

### Vaccination

In the setting with only regular commuting, there were some clusters of protected locations for the highest clustering levels, and the epidemic seemed to be restricted to the hubs. It might therefore be a more effective use of vaccines to allocate all the resources to the most urban locations, since the more rural locations are more protected from the epidemic. However, a different strategy would be to preferentially allocate resources to exactly these rural locations, since the vaccination is more likely to successfully eliminate the risk in these locations.

**Fig 12:**
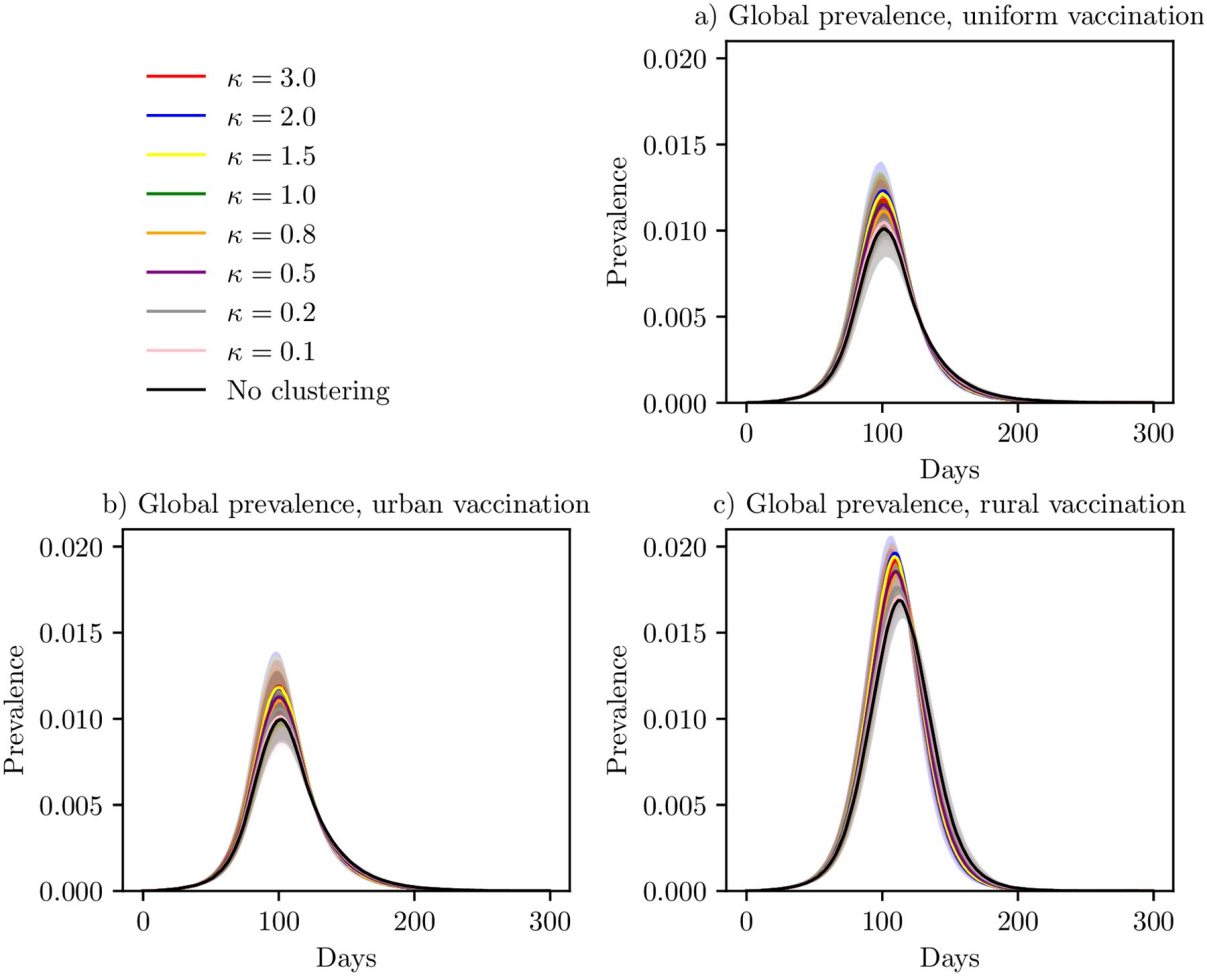
Global prevalence under vaccination. Global prevalence curves for the various clustering levels, under three different vaccination schemes, with 95% confidence bands around the mean. (a) Uniform vaccination, (b) preferential vaccination in urban locations and (c) preferential vaccination in rural locations.

The global prevalence curves in the uniform (pro rata) vaccination setting are given in Fig 12a. The peak timing of the epidemic was similar for the different clustering levels, but there was a higher peak for the higher clustering levels (however note that the confidence bands are overlapping). The mean area not infected and final sizes are given in Table 5. Uniform vaccination reduced the final size substantially for all the clustering levels, compared to the baseline scenario and the travel restriction settings (cf. Tables 1–4). The reduction was slightly larger for the lower levels of clustering (i.e. a 65-66% reduction in final size for the no clustering, *κ* = 0.1 and *κ* = 0.2 versions, compared to a 63-64% reduction for the *κ* ≥ 1 versions).

**Table 5:** Uniform vaccination.

*κ*	Area not infected	Final size
No clustering	0.762 (0.0234)	0.195 (0.0112)
0.1	0.759 (0.0208)	0.198 (0.00978)
0.2	0.764 (0.0183)	0.200 (0.00855)
0.5	0.761 (0.0222)	0.207 (0.00968)
0.8	0.767 (0.0225)	0.205 (0.0115)
1.0	0.760 (0.0253)	0.208 (0.0102)
1.5	0.764 (0.0200)	0.212 (0.00906)
2.0	0.768 (0.0236)	0.212 (0.0103)
3.0	0.777 (0.0217)	0.207 (0.00988)

Percentage of area not infected and final sizes in the situation with a uniform vaccination scheme. Standard deviations are given in parenthesis.

The global prevalence curves for the urban vaccination strategy are given in Fig 12b. The prevalence curves were very similar to the uniform vaccination setting. The mean area not infected and mean final size are given in Table 6. More area was infected in this setting compared to the uniform vaccination setting. The final sizes were slightly smaller in the urban vaccination setting compared to the uniform vaccination setting, for all clustering levels except *κ* = 3.0. The effectiveness of this vaccination strategy compared to the uniform vaccination strategy did not seem to depend on the underlying population clustering of the country. The reduction in final size for the country without clustering was 67% while the reduction under the *κ* = 3.0 scenario was 63%.

**Table 6:** Urban vaccination.

*κ*	Area not infected	Final size
No clustering	0.695 (0.0207)	0.192 (0.0105)
0.1	0.699 (0.0238)	0.194 (0.0113)
0.2	0.701 (0.0233)	0.198 (0.0114)
0.5	0.703 (0.0242)	0.204 (0.0110)
0.8	0.706 (0.0236)	0.202 (0.0109)
1.0	0.703 (0.0227)	0.204 (0.0103)
1.5	0.706 (0.0284)	0.208 (0.0139)
2.0	0.710 (0.0252)	0.208 (0.0121)
3.0	0.706 (0.0236)	0.209 (0.0107)

Percentage of area not infected and final sizes in the situation with a vaccination scheme which preferentially vaccinates urban locations. Standard deviations are given in parenthesis.

The global prevalence curves for the rural vaccination strategy are given in Fig 12c. Again, the peak timing seemed to be quite similar for all the clustering levels, with a higher peak for the higher clustering levels. The mean area not infected and mean final size are given in Table 7. With the rural vaccination strategy, less area was infected, but the final size was a lot larger than for the urban and uniform vaccination strategies, due to both a higher peak prevalence and a longer epidemic. There was only a 42% reduction in final size for the *κ* = 3.0 version, and 44% for the least clustered version. Comparing the final sizes for the rural vaccination strategy and the urban vaccination strategy yields that the difference was larger for the lowest clustering levels.

**Table 7:** Rural vaccination.

*κ*	Area not infected	Final size
No clustering	0.865 (0.0126)	0.323 (0.00399)
0.1	0.860 (0.0119)	0.325 (0.00398)
0.2	0.857 (0.0114)	0.327 (0.00380)
0.5	0.857 (0.0139)	0.328 (0.00463)
0.8	0.856 (0.0135)	0.329 (0.00489)
1.0	0.851 (0.0126)	0.330 (0.00458)
1.5	0.854 (0.0123)	0.332 (0.00450)
2.0	0.859 (0.0126)	0.331 (0.00447)
3.0	0.860 (0.0101)	0.330 (0.00367)

Percentage of area not infected and final sizes in the situation with a vaccination scheme which preferentially vaccinates rural locations. Standard deviations are given in parenthesis.

**Fig 13:**
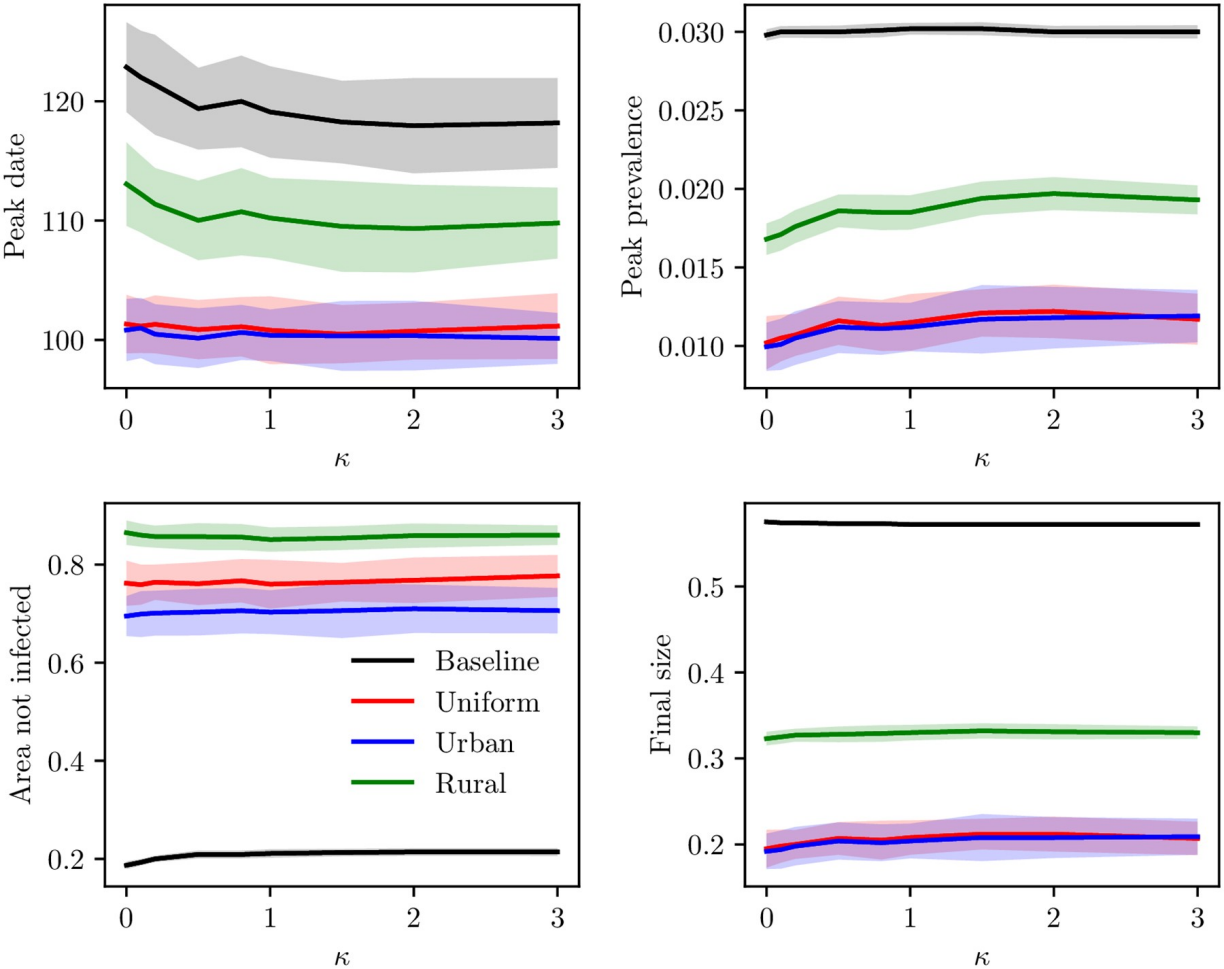
Peak date, peak prevalence, area not infected and final size under vaccination. Peak dates for the global mean prevalence curve, peak prevalence, mean area not infected and mean final size as a function of clustering, with 95% confidence bands. The lines correspond to the baseline scenario, uniform vaccination, urban vaccination and rural vaccination. Top left: peak date. Top right: peak prevalence. Bottom left: area not infected. Bottom right: final size.

We have plotted the peak date for the mean global prevalence curve, peak prevalence, mean area not infected and mean final size for the various levels of clustering, for the baseline scenario and the different vaccination strategies. The plot is given in Fig 13. The peak dates were slightly later for the lower clustering levels than for the higher clustering levels, for the baseline and the rural vaccination strategy. The peak date was similar for the urban and uniform vaccination strategy, while it occurred later for the rural vaccination strategy. All vaccination strategies reduced the peak. The reduction was larger for the uniform and urban vaccination strategies (which were very similar), than for the rural vaccination strategy. The peak prevalence increased slightly with increased clustering under all vaccination schemes. The area infected seemed robust to the population clustering. For the final sizes, we clearly see a reduction with all the vaccination strategies, and the uniform and urban vaccination strategies were the most effective (and very similar), while the rural was a lot less effective. In addition, the final size was quite robust to the underlying clustering, but there was a slightly larger final size for the higher clustering levels.

In S1 Text, we repeated the vaccination simulations, where we assumed that the vaccinated non-immune individuals were 80% less infectious (and not 20% as in the results presented here). The qualitative results were the same, and the final sizes were only slightly smaller for this more optimistic scenario. The urban vaccination strategy was the most effective in reducing final size for all clustering levels, and the uniform vaccination strategy performed similarly.

### Combination strategy

**Fig 14:**
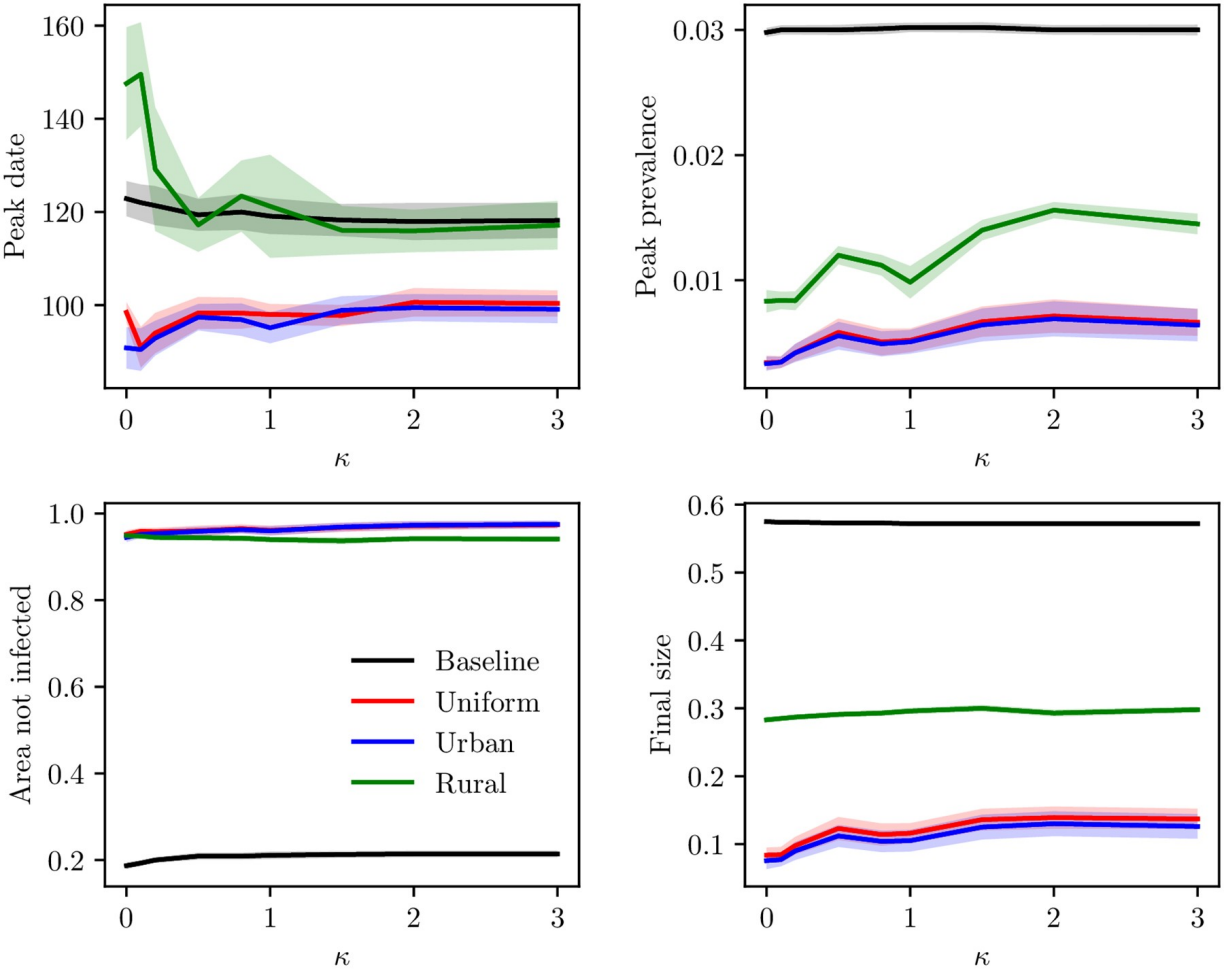
Peak date, peak prevalence, area not infected and final size under combined interventions. Peak dates for the global mean prevalence curve, peak prevalence, mean area not infected and mean final size as a function of clustering, with 95% confidence bands around the mean. The lines correspond to the baseline scenario, uniform vaccination, urban vaccination and rural vaccination. Top left: peak date. Top right: peak prevalence. Bottom left: area not infected. Bottom right: final size.

The impact of the vaccination strategies were quite robust to the underlying population clustering. Since the travel restriction intervention resulted in more protected rural area the higher the clustering, we investigated whether the performance of the vaccination strategies in combination with travel restriction differed from the performance without any travel restrictions. The peak dates, peak prevalences, areas not infected and final sizes for the different strategies and clustering levels are given in Fig 14. The urban and the uniform vaccination strategies were very similar, also when travel restrictions were included. The peak dates were slightly delayed (only with a couple of days) with increased clustering for the urban and the uniform vaccination strategies. Under the rural vaccination strategy, the peak dates occurred earlier for the higher clustering levels than for the lower clustering levels. For the area not infected, the different vaccination strategies were much more similar when travel restrictions were included, and the protected area increased compared to vaccination only or travel restrictions only. The combination strategy further reduced the final size and the peak prevalence for all vaccination strategies, but the qualitative relationship with population clustering was similar to the vaccination only setting. The urban vaccination strategy combined with travel restrictions reduced the final size with 87% for the lowest clustering level, and with 78% for the highest clustering level. For the uniform vaccination strategy, the corresponding reductions were 85% and 76%. Though they were very similar, the difference between the final size for the urban vaccination strategy and the uniform vaccination strategy was larger for the higher clustering levels. Hence for all clustering levels, there was a (small and non-significant) benefit in using the urban vaccination strategy instead of the uniform vaccination strategy, but the benefit was (slightly) larger for higher clustering levels.

## Discussion

We have proposed a method for generating spatial fields with controllable levels of clustering of the population. The clustering is controlled by a design parameter *κ*. Combined with an SEIR model for infectious diseases, we have used this tool to investigate the interplay between infectious disease spread, the effectiveness of interventions and population clustering. This framework is more general and could also easily be applied to the problem of simultaneously investigating the tendency for people to move to cities and the population growth. We have studied the urbanisation phenomenon from a theoretical viewpoint, and hope that this will inspire other, more data-driven and applied studies on urbanisation in specific settings for specific populations.

### Choice of commuting model

In our main analysis, we have chosen to model commuting with a gravity law. The gravity law is a popular choice for modelling influenza/infectious disease spread [52], and has been found to capture well spatio-temporal influenza [53] and measles [54] dynamics. An alternative to the gravity law is the radiation law [51], which is parameter free, and therefore claimed to be more universal. Some analytical inconsistencies of the gravity law are also pointed out in [51]. The radiation law does not directly depend on distance, but rather on the population density between locations. The fact that the radiation law is parameter free is attractive in this setting, since we are modelling commuting in a fictional country and thus obviously do not have commuting data available for that country. However, the gravity law has been shown to have a better fit [55], especially for finer scales, which is the case in our setting. For further information on the gravity law, the radiation law, extensions of these and other mobility models, we refer to the thorough review in [56]. We have chosen to use the gravity law in our main analysis since it had a better fit to our commuting data (*R*^2^ = 0.82 versus *R*^2^ = 0.67 for the radiation law), and because it is commonly used in spatial models of infectious disease transmission. Regarding the shape of the gravity law, we have chosen to use a power function of distance, as in for instance [57], [58], [59], [53] and [54]. Some commuting features that are not well captured by the gravity model are pointed out in [52], and they suggest some extensions to the gravity law for solving these issues. However, for reasons of simplicity, we have chosen to work with the more standard model. In S1 Text, we perform sensitivity analysis on the parameters of the gravity law, the shape of the distance function, and we redo the analysis using a radiation law. The qualitative patterns are robust to the choice of commuting model. However, the different effect sizes varied with the shape of the commuting law. Comments on discrepancies in the effect sizes under the different scenarios are provided below.

### Findings—Discussion, robustness, implications and relation to previous literature

#### Baseline scenario

In a baseline scenario (no travel restrictions, no prior immunity and no vaccination), we found that the population clustering did not play an important role in outcome measures. This finding was robust across a range of different parameter values and choices—for a different set of disease parameters, for a fictional country based on a UK gravity law and population distribution, when varying the length of stay for the long distance travellers, when varying the shape and parameters of the gravity law and when modelling the commuting with a radiation law.

#### Travel restrictions

***Final size***. In contrast with the baseline scenario, population clustering had an effect on the outcome measures under travel restrictions. Most importantly, the final size decreased with increased clustering. The effect of travel restrictions on decreasing the final size was stronger for higher clustering levels. This finding was quite robust to the various parameters and choices, but we note some discrepancies. When halving the distance parameter of the gravity law, travel restrictions effectively reduced the final size for both high and low clustering levels, and hence clustering mattered less in this setting. The decrease in final size under travel restrictions was larger in this setting than for the main analysis. When the distance parameter is decreased, the destination and origin population increase in importance relative to the distance, and hence the commuting patterns become more similar for the different clustering levels. In the limit of lim_*x* → 0_ *d*^*x*^, the commuting networks are equal, and so we expect clustering to be less important for a lower distance parameter. For the UK based country, the reduction in final size with travel restrictions was larger than for the main analysis for higher clustering levels, and smaller for the lower clustering levels. The effect of travel restrictions on reducing final size was smaller for an exponential distance function in the gravity law, and there was no effect for the lowest clustering levels. Doubling the distance parameter in the gravity law, there was also no effect of travel restrictions on reducing final size for the lowest clustering levels. In these two commuting models, the distance is punished more, hence, there is more commuting to proximate locations compared to the gravity law used in the main analysis. Therefore, the country is more well-connected through the commuting network, especially for the lower clustering levels, and travel restrictions on non-commuting travel are most likely therefore less effective.

When commuting was modelled by a radiation law, there was little effect of travel restrictions on reducing final size, and no effect for the lowest clustering levels. This is likely due to the construction of the radiation law, which does not directly depend on distance, but rather on the population density between the locations, suppressing some of the clustering effects that are found with the gravity law. Less area is protected for the radiation law, especially for the lowest clustering levels, indicating that the countries are more well-connected through the commuting network, decreasing the effect of travel restrictions.

We investigated further how the reduction of final size varied by the type of location, that is, how rural or urban the location is. For all but the most urban locations, the final size clearly increased more with increased amount of travel for the higher clustering levels. The effect of travel restrictions on epidemic final size was thus larger for the higher clustering levels, but the effect was most prominent in the less urban areas. This was also the case when varying the length of stay for the long distance travellers, for the different set of disease parameters and for the country based on UK commuting and population size distributions. This is in accordance with [17], where they find no effect of travel restrictions in urban areas. The fact that the effect of travel restrictions was most prominent in the rural areas, might make them less attractive to implement. On the other hand, health care services are more scarce in rural areas than in urban areas, so it might also be attractive that the travel restrictions are most effective in the rural areas. This should be taken into account by policy planners and decision makers.

We note that a 99% travel restriction is a quite strong restriction, and the effect size on the final size is surprisingly low. In addition, there is a problem with compliance, economic costs and how this would work in practice, and such extreme travel restrictions are not very realistic. The vaccination intervention was much more effective in reducing the final size than internal travel restrictions, in agreement with [15, 16]. In [15, 16], they found no effect of internal travel restrictions on final size. We found that internal travel restrictions had some effect on final size, as in [17].

***Peak prevalence and timing***. Internal travel restrictions delayed and reduced the peak. The reduction in peak prevalence was robust to all the settings considered. Under travel restrictions, we found that the higher the clustering, the higher the peak. This finding held for a range of parameter choices and assumptions, but the peak prevalence did not increase with clustering when commuting was modelled by a radiation law. For the exponential distance function in the gravity law, this was only the case for the full travel ban setting.

The peak occurred earlier for higher clustering levels for all the settings, except when the distance parameter was doubled. Travel restrictions were found to delay the peak for most settings. However, for the UK based country, with doubled destination population parameter and the radiation law settings, this was only the case for the lower clustering levels, while there was no delay effect for the higher clustering levels. Overall, the delay effects were smaller for the UK based country, the radiation law setting and when doubling the destination population parameter. Travel restrictions were more effective in delaying the peak for the lower clustering levels. This was true for all the settings considered, but the effect sizes depended on the various parameter choices and settings.

Our results are in agreement with [10, 15–17], where travel restrictions were found to delay the epidemic. In [10], it was found that a 50% travel restriction delayed the epidemic by 1.5 weeks, and we find a delay of 3-10 days, depending on the clustering level. With 90% travel restrictions, [15] found a delay in the epidemic by a few days and [16] found a one week delay in the spread. In our simulations, we found a delay of 4-27 days with 90% travel restrictions, depending on the clustering level. Our results are thus similar to the results in [15] and [16] for the higher clustering levels (more specifically, *κ* = 3.0, *κ* = 2.0 and *κ* = 1.5), while we find larger delay effects for the lower clustering levels.

#### Vaccination

Preferentially vaccinating urban locations was the single most effective strategy in reducing final size, though only slightly better than vaccinating uniformly. This is in accordance with the result in [38], indicating little difference in the final size with a pro rata (uniform) vaccination strategy, and a sequential vaccination by population size-strategy. Preferentially vaccinating the more rural locations was clearly the most inferior strategy. The vaccination was slightly more effective for the lower clustering levels, but the epidemic progression was robust to the population clustering under all three vaccination strategies (in line with the baseline scenario), as opposed to under the travel restriction settings. This may be due to the travel restrictions breaking down specific infection routes, as opposed to the vaccination.

***Combination strategy***. We implemented a combination strategy, combining 99% travel restrictions with the three different vaccination strategies. We found that the most effective strategy for reducing the final size of the epidemic was a combination of travel restrictions with urban vaccination, for all the clustering levels. The difference between the final size for the urban vaccination strategy and the uniform vaccination strategy was (slightly) larger for the higher clustering levels. This is likely because when extensive travel restrictions were imposed, the most rural areas were protected, as we have seen in the analyses. The combination strategy was very efficient in reducing the final size. Travel restrictions further reduced the final size with almost 20 percentage points for the lowest clustering level and 15 percentage points for the highest clustering level. For lower *κ*, the travel restrictions were much more efficient in reducing final size when combined with vaccination, than in isolation.

***Ethical issues***. There are of course ethical issues with spatially targeted vaccination strategies. The CDC (Centers for Disease Control and Prevention) ethical guidelines state that allocation of limited resources should be guided by equity [60]. There are often inconsistencies between the optimal vaccination strategy and the most equitable vaccination strategy [29, 35, 37, 61]. In [32], focus is given to the trade off between equity, simplicity and robustness for efficient spatial allocation policies. Hence, the policy makers have to balance the effectiveness of the vaccine strategy and equity. In our setting, the urban vaccination strategy performed only slightly better than the uniform (fair) vaccination strategy, and hence the uniform vaccination strategy would be recommendable, taking equity into account.

### Limitations

Our study is subject to limitations. The scaling from Norwegian municipalities to block units should be handled with care. The data that we used to fit the population size distribution and gravity law were on a different scale than the block units used in the fictional country, and we can not assess how well these models generalise to the finer block unit scale. In addition, different ways of dividing the population into administrative units could yield different population distributions, which could affect the results. We believe that it is of key importance to model the commuting and the population sizes for the same scale and population, and since commuting data are not available on a finer scale (due to for instance privacy regulations), we have chosen to use the finest scale available for the commuting data. The block units can then be interpreted as a discretisation of administrative units. Census data are often collected for administrative units, but mobile phone data could be used as an information source for commuting on a square gridded fine scale resolution, and would have been an interesting alternative. The fine scale resolution of the fictional country is necessary for the clustering algorithm to provide smooth transitions between the different clustering levels. The fact that we consider the country in isolation and ignore bordering countries can result in edge effects, since the locations on the border might in reality have a connection to the neighbouring countries.

We address some of these limitations in our sensitivity analysis in S1 Text. To handle the fact that different ways of defining administrative regions could affect the population distribution and in turn the results, we also perform the analysis on a country based on population data from the United Kingdom. The population size distribution for Norway was quite heterogeneous compared to other European countries examined, while United Kingdom had a more homogeneous population size distribution. Here, a gravity law fitted to commuting data for the United Kingdom is also used. We also perform sensitivity analysis on the parameters and shape of the gravity law. Though the qualitative patterns were not so sensitive to the choice of commuting model, the effect sizes were not robust to the shape of the commuting law.

### Practical implications

According to the general model developed here, population clustering is an important determinant for the effect of travel restrictions. For high levels of clustering, internal travel restrictions decrease the final size of the epidemic, and this is most prominent in the rural areas. There is no large effect on the peak date for the high clustering levels. For the lower clustering levels, there is less of a benefit in terms of final size reduction. However, for lower clustering levels, the peak date is delayed when implementing internal travel restrictions. That means more time to plan and implement interventions and preventive measures. Internal travel restrictions reduce the peak prevalence for all the clustering levels, reducing the stress on the health care systems. In addition, we found that the higher clustering levels have larger spatial clustering in peak dates. The more spatial clustering in peak dates, the more stress on the health care systems. Whether it is more attractive to delay an epidemic or decrease the final size, depends on the specific influenza strain. If there is high morbidity, such that the patients require more and/or longer health care, it might be of importance to delay the epidemic, to prepare the health care system for the peak period. In addition, if there exists vaccines or other interventions, a delay in the epidemic can be of key importance for the effectiveness of the vaccination programme, since there is more time to distribute (and develop/improve) the vaccine or implement other interventions. The impact of vaccines depends on how early they are introduced (see for instance [25, 33, 62, 63]). For the European countries we considered (by visual inspection), the minimum clustering level was around *κ* = 0.5, and the highest around *κ* = 3.0, and they will likely become even more clustered in the future. This means that according to our model, internal travel restrictions are likely to be less and less effective in delaying epidemics, while they will be more effective in decreasing final sizes (especially in rural areas). In addition, proximate regions will, to an even higher extent than today, experience their peak simultaneously. Hence, it will be even more important to be able to predict the peak timing, in order to prepare the health care system for the peak period. In addition, in order to minimise the final sizes of the epidemic, it is important not to neglect the urban locations for vaccination, and thus specific vaccination sentiment campaigns might target urban locations.

## Supporting information

S1 Text Additional remarks and sensitivity analysis. (PDF)

S1 Fig Population size distribution for the municipalities of Norway. (TIF)

S2 Fig Population densities. Population density in administrative units in Norway, Iceland, Germany, France, Netherlands and United Kingdom.
(TIF)

S3 Fig *τ* = 0: Initial dates. Initial dates for infection when *τ* = 0. These are averages over the simulations where an epidemic occurred in the respective block units. The white locations never experienced the epidemic. Upper left: No clustering. Upper center: *κ* = 0.1. Upper right: *κ* = 0.2. Middle left: *κ* = 0.5. Middle center: *κ* = 0.8. Middle right: *κ* = 1.0. Bottom left: *κ* = 1.5. Bottom center: *κ* = 2.0. Bottom right: *κ* = 3.0.
(TIF)

S4 Fig *τ* = 0: Peak prevalence. Peak prevalence for *τ* = 0. These are averages over the simulations where an epidemic occurred in the respective block units. The white locations never experienced the epidemic. Upper left: No clustering. Upper center: *κ* = 0.1. Upper right: *κ* = 0.2. Middle left: *κ* = 0.5. Middle center: *κ* = 0.8. Middle right: *κ* = 1.0. Bottom left: *κ* = 1.5. Bottom center: *κ* = 2.0. Bottom right: *κ* = 3.0.
(TIF)

S5 Fig *τ* = 0: Probability of epidemic. Probability of infection for *τ* = 0. Upper left: No clustering. Upper center: *κ* = 0.1. Upper right: *κ* = 0.2. Middle left: *κ* = 0.5. Middle center: *κ* = 0.8. Middle right: *κ* = 1.0. Bottom left: *κ* = 1.5. Bottom center: *κ* = 2.0. Bottom right: *κ* = 3.0.
(TIF)

S6 Fig Global prevalence under delayed travel restrictions. Estimated global prevalence for the various smoothing levels with corresponding 95% confidence bands, in the setting with delay in implementation of travel restrictions.
(TIF)

S7 Fig Final size versus travel ratio with varying travel duration. Final size versus *τ* for various clustering levels, *κ*, when the length of stay for non-commuting travellers varies, with corresponding 95% confidence bands. a) All locations. b) Locations with population size smaller than the 25% quantile. c) Locations with population size between the 25% and 50% quantile. d) Locations with population size between the 50% quantile and the 75% quantile. e) Locations with population size larger than the 75% quantile.
(TIF)

S8 Fig Peak dates versus travel ratio with varying travel duration. Peak date versus *τ* for various clustering levels, *κ*, when the length of stay for non-commuting travellers varies, with corresponding 95% confidence bands.
(TIF)

S9 Fig Final size and peak date with travel ban targeting infectious symptomatic. Final size and peak date versus *τ* for various clustering levels, *κ*, with only travel restrictions for the infectious symptomatic, with corresponding 95% confidence bands.
(TIF)

S10 Fig Final size versus travel ratio with alternative disease parameters. Final size versus *τ* for various clustering levels, *κ*, for the alternative disease parameters, with corresponding 95% confidence bands. a) All locations. b) Locations with population size smaller than the 25% quantile. c) Locations with population size between the 25% and 50% quantile. d) Locations with population size between the 50% quantile and the 75% quantile. e) Locations with population size larger than the 75% quantile.
(TIF)

S11 Fig Peak date versus travel ratio for alternative disease parameters. Peak date versus *τ* for various clustering levels, *κ*, with corresponding 95% confidence bands. The results are for the alternative disease parameters.
(TIF)

S12 Fig Final size versus travel ratio for the UK based country. Final size versus *τ* for various clustering levels, *κ*, with corresponding 95% confidence bands. The results are in the countries based on data from the United Kingdom. a) All locations. b) Locations with population size smaller than the 25% quantile. c) Locations with population size between the 25% and 50% quantile. d) Locations with population size between the 50% quantile and the 75% quantile. e) Locations with population size larger than the 75% quantile.
(TIF)

S13 Fig Peak date versus travel ratio for the UK based country. Peak date versus *τ* for various clustering levels, *κ*, with corresponding 95% confidence bands. The results are in the country based on data from the United Kingdom.
(TIF)

S14 Fig Sensitivity analysis (halving distance parameter): Peak dates, peak prevalence, area not infected and final size. Peak dates for the global mean prevalence curve, peak prevalence, mean area not infected and mean final size as a function of clustering, with 95% confidence bands, when the distance parameter of the gravity law was halved. The lines correspond to the baseline scenario, 90% travel restrictions, 99% travel restrictions and 100% travel restrictions. Top left: peak date. Top right: peak prevalence. Bottom left: area not infected. Bottom right: final size.
(TIF)

S15 Fig Sensitivity analysis (halving distance parameter): Final size and peak date. Final size and peak date versus *τ* for various clustering levels, *κ*, with corresponding 95% confidence bands, when the distance parameter of the gravity law was halved.
(TIF)

S16 Fig Sensitivity analysis (doubling distance parameter): Peak dates, peak prevalence, area not infected and final size. Peak dates for the global mean prevalence curve, peak prevalence, mean area not infected and mean final size as a function of clustering, with 95% confidence bands, when the distance parameter of the gravity law was doubled. The lines correspond to the baseline scenario, 90% travel restrictions, 99% travel restrictions and 100% travel restrictions. Top left: peak date. Top right: peak prevalence. Bottom left: area not infected. Bottom right: final size.
(TIF)

S17 Fig Sensitivity analysis (doubling distance parameter): Final size and peak date. Final size and peak date versus *τ* for various clustering levels, *κ*, with corresponding 95% confidence bands, when the distance parameter of the gravity law was doubled.
(TIF)

S18 Fig Sensitivity analysis (halving destination population parameter): Peak dates, peak prevalence, area not infected and final size. Peak dates for the global mean prevalence curve, peak prevalence, mean area not infected and mean final size as a function of clustering, with 95% confidence bands, when the destination population parameter of the gravity law was halved. The lines correspond to the baseline scenario, 90% travel restrictions, 99% travel restrictions and 100% travel restrictions. Top left: peak date. Top right: peak prevalence. Bottom left: area not infected. Bottom right: final size.
(TIF)

S19 Fig Sensitivity analysis (halving destination population parameter): Final size and peak date. Final size and peak date versus *τ* for various clustering levels, *κ*, with corresponding 95% confidence bands, when the destination population parameter of the gravity law was halved.
(TIF)

S20 Fig Sensitivity analysis (doubling destination population parameter): Peak dates, peak prevalence, area not infected and final size. Peak dates for the global mean prevalence curve, peak prevalence, mean area not infected and mean final size as a function of clustering, with 95% confidence bands, when the destination population parameter of the gravity law was doubled. The lines correspond to the baseline scenario, 90% travel restrictions, 99% travel restrictions and 100% travel restrictions. Top left: peak date. Top right: peak prevalence. Bottom left: area not infected. Bottom right: final size.
(TIF)

S21 Fig Sensitivity analysis (doubling destination population parameter): Final size and peak date. Final size and peak date versus *τ* for various clustering levels, *κ*, with corresponding 95% confidence bands, when the destination population parameter of the gravity law was doubled.
(TIF)

S22 Fig Peak dates, peak prevalence, area not infected and final size, exponential distance function. Peak dates for the global mean prevalence curve, peak prevalence, mean area not infected and mean final size as a function of clustering, with 95% confidence bands, with an exponential function of distance in the gravity law. The lines correspond to the baseline scenario, 90% travel restrictions, 99% travel restrictions and 100% travel restrictions. Top left: peak date. Top right: peak prevalence. Bottom left: area not infected. Bottom right: final size.
(TIF)

S23 Fig Final size and peak date with exponential distance function. Final size and peak date versus *τ* for various clustering levels, *κ*, with corresponding 95% confidence bands, with an exponential function of distance in the gravity law.
(TIF)

S24 Fig Peak dates, peak prevalence, area not infected and final size, range parameter 10.0 in the covariance function. Peak dates for the global mean prevalence curve, peak prevalence, mean area not infected and mean final size as a function of clustering, with 95% confidence bands, when the range parameter of the Matérn covariance function was increased from 5.0 to 10.0. The lines correspond to the baseline scenario, 90% travel restrictions, 99% travel restrictions and 100% travel restrictions. Top left: peak date. Top right: peak prevalence. Bottom left: area not infected. Bottom right: final size.
(TIF)

S25 Fig Final size and peak date with range parameter 10.0 in the covariance function. Final size and peak date versus *τ* for various clustering levels, *κ*, with corresponding 95% confidence bands, when the range parameter of the Matérn covariance function was increased from 5.0 to 10.0.
(TIF)

S26 Fig Peak dates, peak prevalence, area not infected and final size, when the radiation law was used to model commuting. Peak dates for the global mean prevalence curve, peak prevalence, mean area not infected and mean final size as a function of clustering, with 95% confidence bands, when the commuting was implemented by the radiation law. The lines correspond to the baseline scenario, 90% travel restrictions, 99% travel restrictions and 100% travel restrictions. Top left: peak date. Top right: peak prevalence. Bottom left: area not infected. Bottom right: final size.
(TIF)

S27 Fig Final size and peak date when the radiation law was used to model commuting. Final size and peak date versus *τ* for various clustering levels, *κ*, with corresponding 95% confidence bands, for the results where commuting was implemented by the radiation law.
(TIF)

S28 Fig Peak dates, peak prevalence, area not infected and final size, 20% reduced infectiousness. Peak dates for the global mean prevalence curve, peak prevalence, mean area not infected and mean final size as a function of clustering, with 95% confidence bands, when the assumed infectiousness of non-immune vaccinated is reduced by 20%. The lines correspond to the baseline scenario, uniform vaccination, urban vaccination and rural vaccination. Top left: peak date. Top right: peak prevalence. Bottom left: area not infected. Bottom right: final size.
(TIF)

S1 Table Estimated *a* for urban and rural locations. Estimated power *a* for final size = *τ*^*a*^+ *b*, for different levels of clustering, for the Q1 (most rural), Q2, Q3 and Q4 (most urban) locations.
(PDF)

S2 Table Estimated *a* for all locations. Estimated power *a* for final size = *τ*^*a*^+ *b*, for different levels of clustering.
(PDF)

S3 Table Delayed travel restrictions. Global peak day, global peak prevalence, percentage of area not infected and final sizes in the situation with a delay in the implementation of the travel restrictions. Standard deviations are given in parenthesis.
(PDF)
