# Supplementary material for: A theoretical single-parameter model for urbanisation to study infectious disease spread and interventions
Source: PLoS Comput Biol. 2019 Mar 7;15(3):e1006879. doi: 10.1371/journal.pcbi.1006879 (PMC6424465; doi:10.1371/journal.pcbi.1006879)
Supplement: S1 Text — (PDF) [file pcbi.1006879.s001.pdf]

Supporting information

S1 Text. Additional remarks and sensitivity analysis

Estimated population density We fit a gamma distribution to the logarithm of the population size for all municipalities of Norway. The histogram of the population size for the municipalities of Norway and the fitted distribution are given in S1 Fig. S1 Fig also shows a QQ-plot for the fitted distribution, and we see that a gamma distribution fits the data well. The estimated density for population size, N, is

$$f(N) = \text{Gamma}(31.47, 3.97) = \frac{3.97^{31.47} \cdot N^{30.47} \cdot \exp(-3.97N)}{\Gamma(31.47)}.$$

Population clustering in various countries In order to assess a realistic range for κ, we plot the population density in administrative regions for various countries. This also gives us an idea of their relative levels of population clustering. The countries we consider are Norway (Statistics Norway, 2016), Iceland (data for 2017 from Statistics Iceland), Germany [1, 2], France [3], the Netherlands [4] and the United Kingdom (Office for National Statistics GB, estimates for 2016), given in S2 Fig. The shape files are downloaded from GADM. We note that Iceland seems to have the highest population clustering level (similar to the $\kappa = 3.0$ country). Norway and France also seem to have a high population clustering, similar to the $\kappa = 1.5$ or $\kappa = 2.0$ countries. The United Kingdom is somewhere between $\kappa = 1.5$ and $\kappa = 1.0$. The Netherlands and Germany are less clustered countries, with more cities spread out geographically, with Germany being the least clustered country. Germany seems to be somewhere between the $\kappa = 0.5$ and $\kappa = 0.8$ country, while the Netherlands seem similar to something between the $\kappa = 0.8$ and $\kappa = 1.0$ country. Similarities are found by visual inspection. A formal estimation of κ is beyond the scope of this paper.

Initial dates, peak prevalence and probability of epidemic in the commuting-only setting We plotted the initial dates, peak prevalences and

probabilities of experiencing an epidemic in each block unit, for $\tau = 0$. The initial date was defined as the date where, for seven consecutive days, the prevalence is larger than a baseline. The baseline is 1.0%, except from in the locations where the population size is less than 100, then the baseline is one case. The peak prevalence is the proportion of infected individuals on the peak day (the day with the largest number of infected symptomatic individuals). The initial dates are given in S3 Fig, the peak prevalences are given in S4 Fig and the probabilities of experiencing the epidemic are given in S5 Fig. By visual inspection, we found spatial clustering in both the initial dates, peak incidences and probabilities of experiencing the epidemic, and that the spatial clustering increased with increasing (population) clustering levels.

The plot of initial dates (S3 Fig) seemed very similar to the plot of peak dates (cf. Fig 6 in the manuscript). We computed the correlations between initial dates and peak dates for all the different travel restriction settings and clustering levels, and found a high correlation, ranging from 0.87 to 0.99.

Fitted function of final size versus τ We fitted functions of the form $y = \tau^a + b$, where y is the final size, for the different population size locations. The parameter of interest is a, which determines the functional dependence on τ. The results are given in S1 Table. We found that for Q1, Q2 and Q3, the best fitting a decreased with increased clustering, so the increase was higher for the higher levels of clustering. This also seemed to be the trend for Q4, but the relationship was not monotone. We also did this for the final size of all locations combined. The results are given in S2 Table. With all locations combined, we also found that a decreased with increased clustering, so the more clustering, the faster increase.

Delayed travel restrictions It might not be reasonable that travel restrictions within a country would be implemented before the first case is detected. There is therefore likely a delay in implementation of the travel restriction. We investigated the consequences of this delay by simulating in the setting where the travel restrictions were implemented 50 days after the first introduction event, in the setting with 99% travel restrictions. The global prevalence curves are given in S6 Fig. Peak dates, peak prevalences, final sizes and proportion of area not infected are given in S3 Table.

Comparing with the setting where the travel restrictions were implemented immediately in Table 3 in the main text, we note that the effect on delaying the epidemic was smaller when the restrictions were implemented later. Comparing with $\tau = 1/100$ (90% travel restrictions), we note that the epidemic was more delayed with immediate implementations of 90% travel restrictions than with a 50-days delay of 99% travel restrictions. Considering the final sizes, we note that they were less affected by the delay in the implementation. The final sizes were smaller with a 50-days delayed 99% travel restriction than for the immediate 90% travel restriction.

Varying the length of stay For the non-commuters, we have let their length of stay be 24 hours. We investigated how sensitive the results were to this assumption, by varying the length of stay uniformly between one and five days. Hence, every non-commuter stayed at their destination location for one, two, three, four or five days, with equal probability. The final sizes versus travel ratio for the various clustering levels are given in S7 Fig. The shapes of the curves are very similar to the results where the length of stay was 24 hours for all travellers. Hence, the overall pattern did not seem to be sensitive to the length of stay. For all clustering levels, the final size increased with increased amount of non-commuting travel, and the growth was larger for the higher clustering levels. For the Q1, Q2 and Q3 locations, the final size had a larger increase with non-commuting travel for the higher clustering levels, while for the Q4 locations, the curve quickly levelled off. There was however a slightly larger final size with increased amount of non-commuting travel in this setting compared to the fixed length of stay, and this was most prominent in the Q1 and Q2 locations.

The peak dates versus travel ratio for the various clustering levels are given in S8 Fig. The pattern was again the same, with the lower clustering levels having a later peak, and an earlier peak date with increased travel ratio. The delay in peak dates under travel restrictions was larger the less clustering. The advance in peak date with increased amount of non-commuting travel was slightly larger in the setting with a varying length of stay, than in the setting where all trips lasted for 24 hours. We note that the confidence bands are quite wide, but there is no overlap between the highest and lowest clustering levels.

Travel restriction targeting infectious individuals We investigated the setting where only symptomatic, infectious individuals are restricted from (non-commuting) travelling. This only suppresses one of the two possible paths of disease spread from one location to another. Infectious individuals can no longer travel to a susceptible location and infect it, but susceptible individuals can travel to an infectious location and infect their home location. The final sizes and peak dates for the different clustering levels, as functions of travel ratio, are given in S9 Fig. Here, the travel ratio is only for the infectious individuals, while the rest of the population travel according to the baseline scenario. The effect of travel restrictions on the final size was a lot smaller when we only restricted travelling for infectious individuals. The relationship between the final size and the travel ratio seemed to be quite similar for the different levels of population clustering, and we found no protective effect for the higher clustering levels in terms of final size. The differences in peak dates were also a lot smaller in this setting, and the confidence bands are overlapping for almost all clustering levels.

Different disease parameters In order to test sensitivity to the disease parameters, we performed simulations with different parameters. The parameters were the same as in [5], where they use an SEIR-model to model a SARS epidemic. The average incubation period was 5.0 days, the average infectious period was 4.0 days and the basic reproductive number was 2.37.

The final sizes versus travel ratio are given in S10 Fig. We found a rapid increase in final size with increased amount of non-commuting travel for the higher clustering levels. The final sizes for the various population size areas (the Q1, Q2, Q3 and Q4 areas) are given in S10 Fig. As for the main results, the final size had a larger increase with travel ratio for higher κ in the Q1, Q2 and Q3 locations, while there was no difference in the most urban (Q4) locations. Hence, the most rural locations were more protected from the epidemic for the higher clustering levels for lower travel ratios. The peak dates versus travel ratio are given in S11 Fig. The pattern was the same as for the parameters used in the main analysis, with a delay in peak dates under travel restrictions, and a larger delay for the lower clustering levels.

Country based on United Kingdom In order to investigate how sensitive the results are to using data from Norway to fit the population sizes and commuting patterns, we performed an analysis where we used population data from the United Kingdom. The population size distribution we used is the one in Fig 1 in the main text. We used the fitted gravity law from [6] which is fitted to an aggregation of 8850 wards in England and Wales.

As before, we plot the final size of the epidemic versus travel ratio for the different κ. The final sizes are given in S12 Fig. We found that also for the United Kingdom-based country, the final size was smaller for the higher clustering levels, under travel restrictions. The final size for the country with no clustering was 28% higher than the final size for $\kappa = 3.0$, under the complete travel ban scenario. This was larger than the 14% difference found in the main analysis. We also found that the effect of travel restrictions on reducing the final size was larger for the higher clustering levels. For the complete travel ban, the reduction was 28% for $\kappa = 3.0$ and 2% for the no clustering scenario. This was a larger reduction for $\kappa = 3.0$ than the results for the main analysis (21% reduction), and a smaller reduction for the no clustering-setting (9% reduction in the main analysis). We plotted the final sizes for the various population size areas. The population size quantiles are for the United Kingdom population, where Q1, Q2, Q3 and Q4 are defined as before. The final sizes for Q1, Q2, Q3 and Q4 areas are given in S12 Fig. The Q1, Q2 and Q3 areas were more protected for the higher clustering levels, under travel restrictions. For the Q4 areas, travel restrictions did not seem to have an effect regardless of clustering level. The peak dates versus travel ratio are given in S13 Fig. The pattern in peak dates was less clear than for the Norway-based country, but we found that with increased amount of non-commuting travel, the peak date was advanced for the lower clustering levels, as we also had before. Hence, the effect of travel restrictions on delaying the peak was larger for the lower clustering levels. There was no delay in the peak with travel restrictions for the highest clustering levels, while the delay was slightly more than two weeks for the lowest clustering levels. These delay effects were smaller than for the main analysis, where we found a delay of up to one week for the highest clustering levels, and up to five weeks for the lowest clustering levels.

Gravity law parameters We perform single parameter sensitivity analyses by varying the gravity law parameters in the following way: 1) halving the distance parameter, 2) doubling the distance parameter, 3) halving the destination population parameters and 4) doubling the destination population parameter. We have plotted the peak date for the mean global prevalence curve, peak prevalence, mean area not infected and mean final size for the various levels of clustering, for the baseline scenario and the travel restriction scenarios (S14 Fig, S16 Fig, S18 Fig and S20 Fig, for the halving of the distance parameter, doubling of the distance parameter, halving of the destination population parameter and doubling of the destination population parameter, respectively). The qualitative results were similar to the main analysis (cf. Fig 9). The peak dates occurred earlier for the higher clustering levels than for the lower clustering levels, for the halving of the distance parameter and the doubling of the destination population parameter. This was also the case for the halving of the destination population parameter, but the pattern was less clear than for the main analysis. For the doubling of the distance parameter, there was no apparent relation between peak dates and clustering. The peak prevalence increased with increased clustering levels for all settings, except for the doubling of the destination population parameter, where the relationship was not monotone. The amount of area which was not infected increased with increased clustering for all the settings. In general, the final size decreased with increased clustering. For the halving of the distance parameter and the doubling of the destination population parameter, $\kappa = 3.0$ deviated from this monotone pattern. For the doubling of the distance parameter, the relationship between final size and clustering did not seem monotone for the $\tau = 0$-scenario. The peak dates and final sizes versus τ are given in S15 Fig, S17 Fig, S19 Fig and S21 Fig, for the halving of the distance parameter, doubling of the distance parameter, halving of the destination population parameter and doubling of the destination population parameter, respectively. The peak dates were clearly delayed with travel restrictions for all clustering levels for the halving of the distance parameter and the halving of the destination population parameter, and the delay was larger for the lower clustering levels than for the higher clustering levels. For a doubling of the distance parameter, the pattern was less clear, but the delay of peak dates was larger for the lower clustering levels. For the doubling of the destination population parameter, the peak date was

actually not delayed by travel restrictions for the higher clustering levels (on the contrary, they were actually slightly advanced), while the pattern was as for the main analysis for the lower clustering levels (cf. Fig 11), however the delay effect was lower. The final sizes decreased with increased travel restrictions for all clustering levels, for all the settings, just like in the main analysis (cf. Fig 10a). In addition, the decrease was larger for the higher clustering levels, but for the setting with a halving of the distance parameter, the differences between the clustering levels were very small. For the halving of the distance parameter, the decrease in final size with travel restrictions was larger than for the main analysis. Hence, the qualitative results were quite robust to the distance parameter and destination population parameter of the gravity law. Note that these parameters are not necessarily realistic, since they are not fitted to data.

Exponential distance function The travel restriction analysis was repeated, where we fitted a different gravity law to the commuting data. We used an exponential distance function, resulting in the gravity law

$$w_{ij} \propto \frac{N_i^{0.50} N_j^{0.23}}{\exp\left(0.00011 d_{ij}\right)},$$

where, as before, w_{ij} is the number of commuters from location i to location j, N_i is the population size in location i, N_j is the population size in location j and d_{ij} is the distance (in meters) between the locations i and j. Note that this shape of commuting law had a worse fit to the commuting data ($R^2 = 0.66$ versus $R^2 = 0.82$ with the power kernel).

We have plotted the peak date for the mean global prevalence curve, peak prevalence, mean area not infected and mean final size for the various levels of clustering, for the baseline scenario and the travel restrictions (S22 Fig). The qualitative results are similar to the main analysis. The peak dates decreased with increased clustering. Considering the peak prevalences, they were robust to the underlying clustering, except from under the full travel ban scenario, where they increased with increased clustering. The decrease in peak prevalence under travel restrictions was larger than for the main analysis. The proportion of protected area increased with increased clustering, and the final size decreased with increased clustering, just like in the main analysis. The peak

dates and final sizes versus travel ratio are given in S23 Fig. We clearly see that the peak dates were delayed with travel restrictions for all clustering levels, and that the delay was larger for the lower clustering levels than for the higher clustering levels. The final sizes also increased with increased travel ratio, but the increase was largest for the lowest travel ratios, and the relationship quickly levels off. The decrease in final size with travel restrictions was largest for the highest clustering levels as in the main analysis, but extensive travel restrictions are required to have an effect. Hence, the effect of travel restrictions on final sizes were larger with a power kernel in the gravity law.

Range parameter of the covariance function The travel restriction analysis was repeated, where we increased the range parameter of the covariance function that was used to generate the clustering levels, from 5.0 to 10.0. We have plotted the peak date for the mean global prevalence curve, peak prevalence, mean area not infected and mean final size for the various levels of clustering, for the baseline scenario and the travel restrictions. The plot is given in S24 Fig. We clearly see that the qualitative results were the same as for the main analysis. In the travel restriction scenarios, the final sizes were higher for the lower clustering levels compared to the higher clustering levels. The peak prevalence was higher for the higher clustering levels. The peak date was earlier for the higher clustering levels, and the amount of protected area increased with increased clustering. The peak dates and final sizes versus travel ratio are given in S25 Fig. We clearly see that the peak dates were delayed with travel restrictions for all clustering levels, and that the delay was larger for the lower clustering levels than for the higher clustering levels. The final sizes decreased with increased travel restrictions for all clustering levels, and the decrease was larger for the higher clustering levels. These results were the same as for the main analysis.

Radiation law The radiation law is an alternative to the gravity law. We performed the analyses with a radiation law for modelling the commuting, where it was assumed that the proportion of commuters in each block unit (location) was proportional to the population size. Note that the radiation law had a worse fit to the commuting data ($R^2 = 0.67$ versus $R^2 = 0.82$ for the gravity law).

We have plotted the peak date for the mean global prevalence curve, peak

prevalence, mean area not infected and mean final size for the various levels of clustering, for the baseline scenario and the travel restriction scenarios. The plot is given in S26 Fig. The qualitative results were similar to the main analysis. The peak dates decreased with increased clustering, with an exception of $\kappa = 1.0$, which deviated from the pattern. The relationship between peak prevalence and clustering was not monotone. The proportion of protected area increased with increased clustering, and the final size decreased with increased clustering, just like in the main analysis. The peak dates and final sizes versus travel ratio are given in S27 Fig. For the highest clustering levels, travel restrictions did not delay the peak, while for the lower clustering levels, travel restrictions delayed the peak, and the delay was larger the less clustering. We clearly see that the final sizes increased with increased travel ratio, and that the effect of travel restrictions was larger the higher the clustering. The effects of travel restrictions on reducing the final size were smaller than for the main analysis.

Infectiousness of non-immune vaccinated individuals In our vaccination scheme, we have assumed that 70% of those who were vaccinated gained immunity. In the main analysis, we assumed that those individuals who were vaccinated but not immune, had a 20% reduced infectiousness. Here, we present the results for a more optimistic scenario, where they had an 80% reduced infectiousness.

The peak date for the mean global prevalence curve, peak prevalence, mean area not infected and mean final size for the various levels of clustering, for the baseline scenario and the different vaccination strategies are given in S28 Fig. As for the main analysis, the epidemic seemed robust to the underlying population clustering, under the various vaccination strategies. All vaccination strategies reduced the peak and the final size, and the reductions were larger for the uniform and urban vaccination strategies (which performed very similar), than for the rural vaccination strategy. Hence, the qualitative results were very similar to the setting in the main analysis. Quantitatively, the final sizes and peak prevalences were slightly smaller in this setting.

References

1. Wikipedia contributors. Liste der Landkreise in Deutschland — Wikipedia, The

Free Encyclopedia; 2018. Available from:
https://de.wikipedia.org/wiki/Liste_der_Landkreise_in_Deutschland.
Cited 24 April 2018

2. Wikipedia contributors. Liste der kreisfreien Städte in Deutschland— Wikipedia, The Free Encyclopedia; 2018. Available from: https://de.wikipedia.org/wiki/Liste_der_kreisfreien_Stadte_in_Deutschland. Cited 24 April 2018

3. Wikipedia contributors. List of arrondissements of France — Wikipedia, The Free Encyclopedia; 2018. Available from: https://en.wikipedia.org/wiki/List_of_arrondissements_of_France. Cited 24 April 2018

4. Wikipedia contributors. List of municipalities of the Netherlands — Wikipedia, The Free Encyclopedia; 2018. Available from: https://en.wikipedia.org/wiki/List_of_municipalities_of_the_Netherlands. Cited 24 April 2018

5. Wang J, McMichael AJ, Meng B, Becker NG, Han W, Glass K, et al. Spatial dynamics of an epidemic of severe acute respiratory syndrome in an urban area. Bull World Health Organ. 2006;84(12):965–968.

6. Masucci AP, Serras J, Johansson A, Batty M. Gravity versus radiation models: On the importance of scale and heterogeneity in commuting flows. Phys Rev E Stat Nonlin Soft Matter Phys. 2013;88(2):022812.

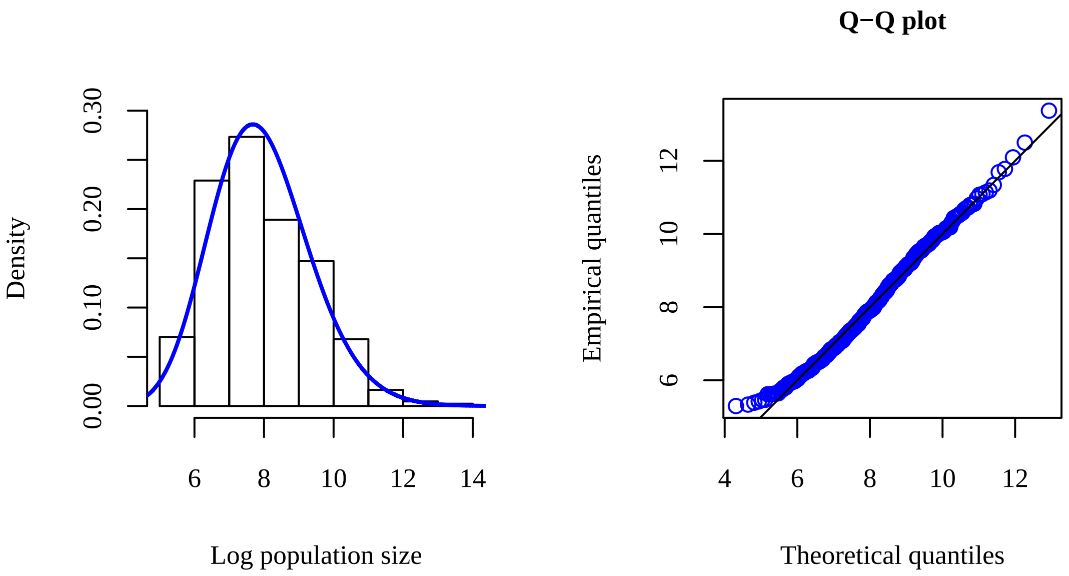

Population size distribution for the municipalities of Norway.

S1 Fig.

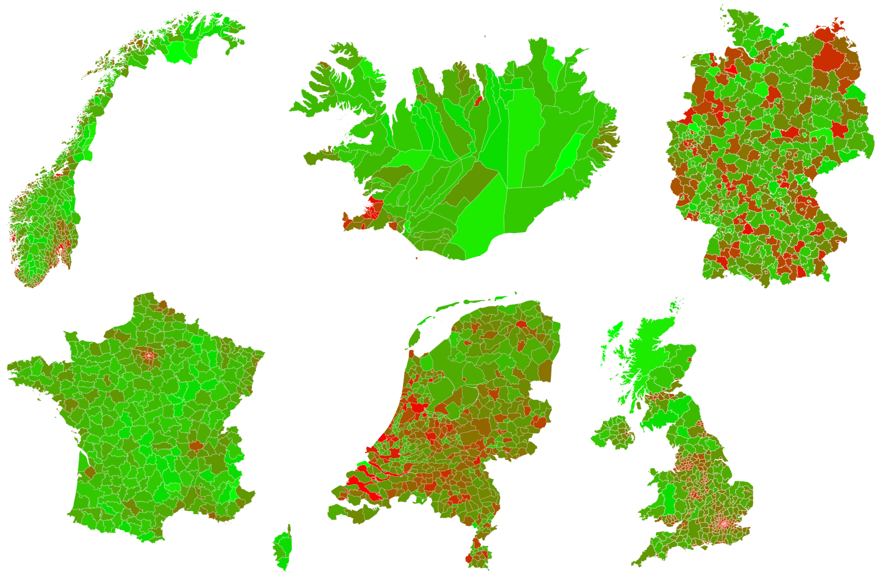

Population densities. Population density in administrative units in Norway, Iceland, Germany, France, Netherlands and United Kingdom.

S2 Fig.

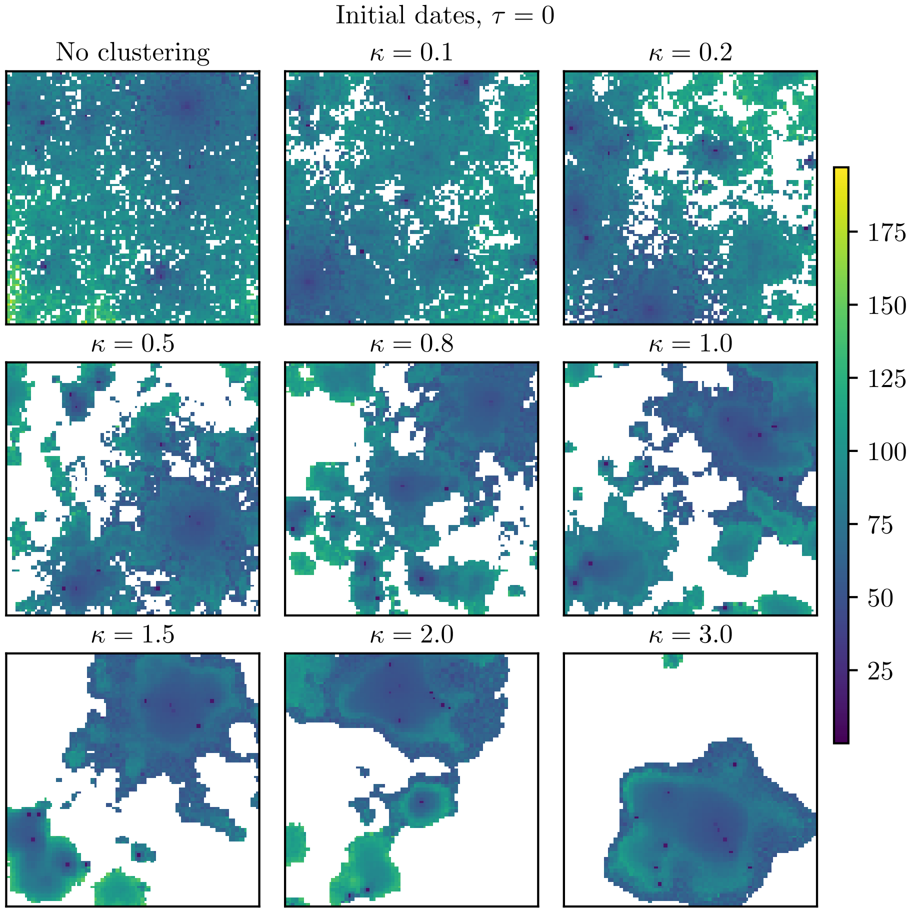

τ= 0: initial dates. Initial dates for infection when $\tau = 0$. These are averages over the simulations where an epidemic occurred in the respective block units. The white locations never experienced the epidemic. Upper left: No clustering. Upper center: $\kappa = 0.1$. Upper right: $\kappa = 0.2$. Middle left: $\kappa = 0.5$. Middle center: $\kappa = 0.8$. Middle right: $\kappa = 1.0$. Bottom left: $\kappa = 1.5$. Bottom center: $\kappa = 2.0$. Bottom right: $\kappa = 3.0$.

S3 Fig.

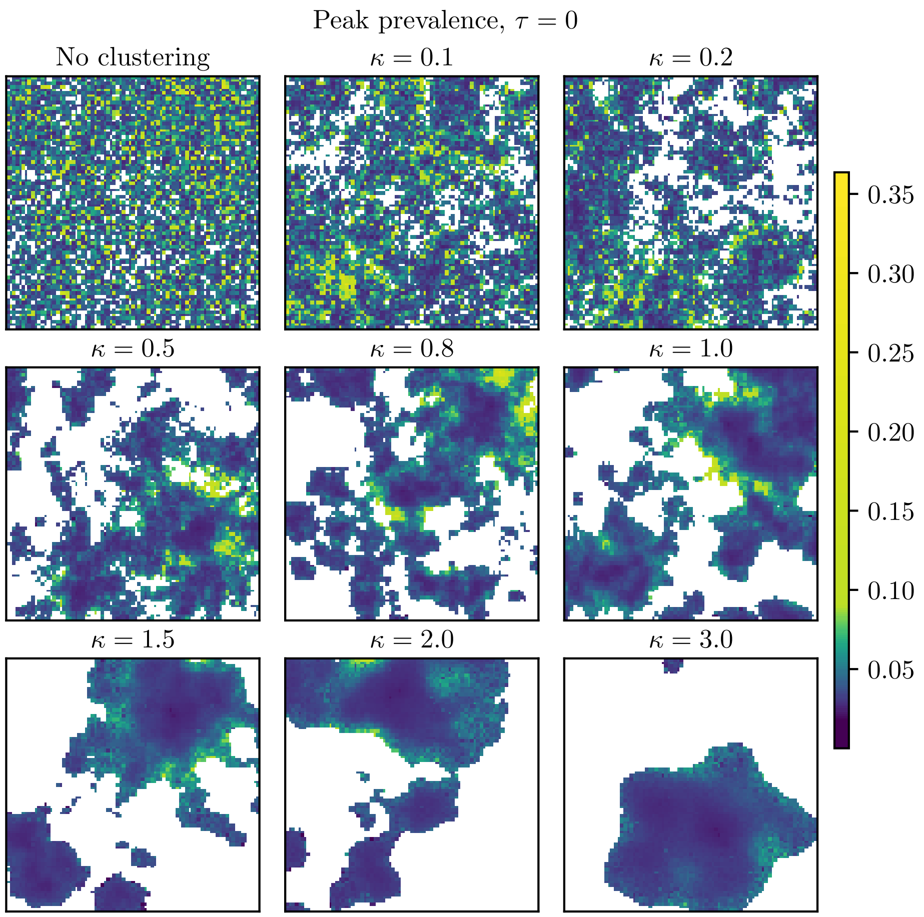

τ= 0: peak prevalence. Peak prevalence for $\tau = 0$. These are averages over the simulations where an epidemic occurred in the respective block units. The white locations never experienced the epidemic. Upper left: No clustering. Upper center: $\kappa = 0.1$. Upper right: $\kappa = 0.2$. Middle left: $\kappa = 0.5$. Middle center: $\kappa = 0.8$. Middle right: $\kappa = 1.0$. Bottom left: $\kappa = 1.5$. Bottom center: $\kappa = 2.0$. Bottom right: $\kappa = 3.0$.

S4 Fig.

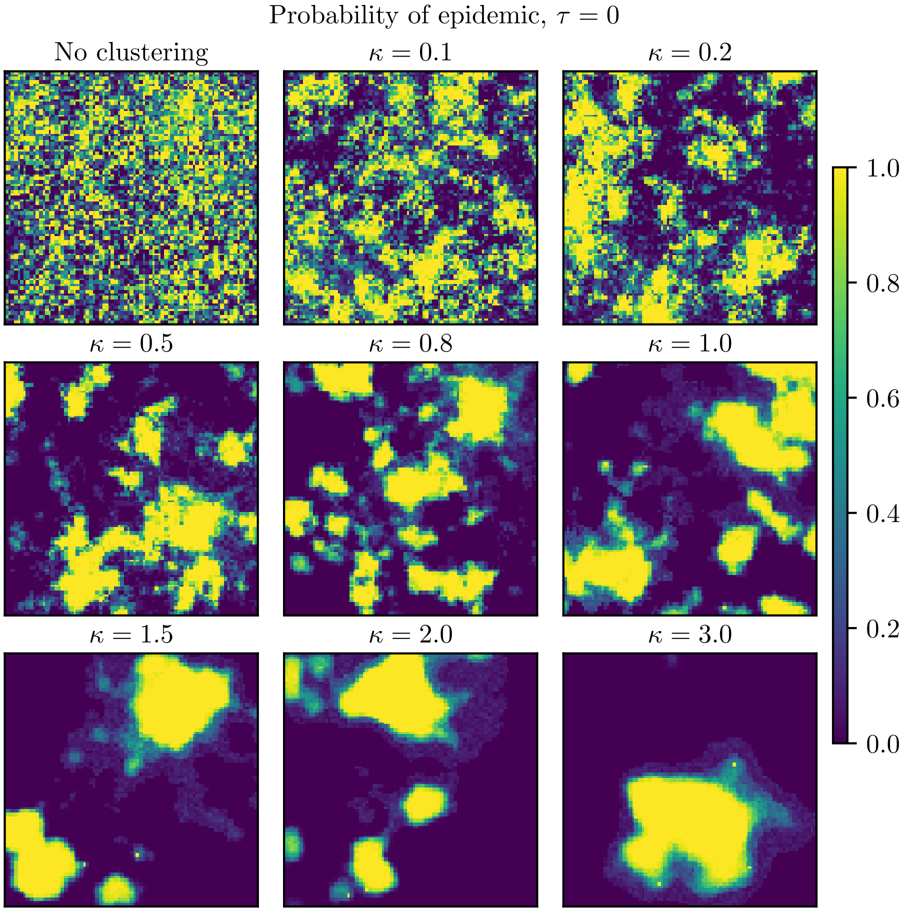

$\boldsymbol{\tau}$= 0: probability of epidemic. Probability of infection for $\tau = 0$. Upper left: No clustering. Upper center: $\kappa = 0.1$. Upper right: $\kappa = 0.2$. Middle left: $\kappa = 0.5$. Middle center: $\kappa = 0.8$. Middle right: $\kappa = 1.0$. Bottom left: $\kappa = 1.5$. Bottom center: $\kappa = 2.0$. Bottom right: $\kappa = 3.0$.

S5 Fig.

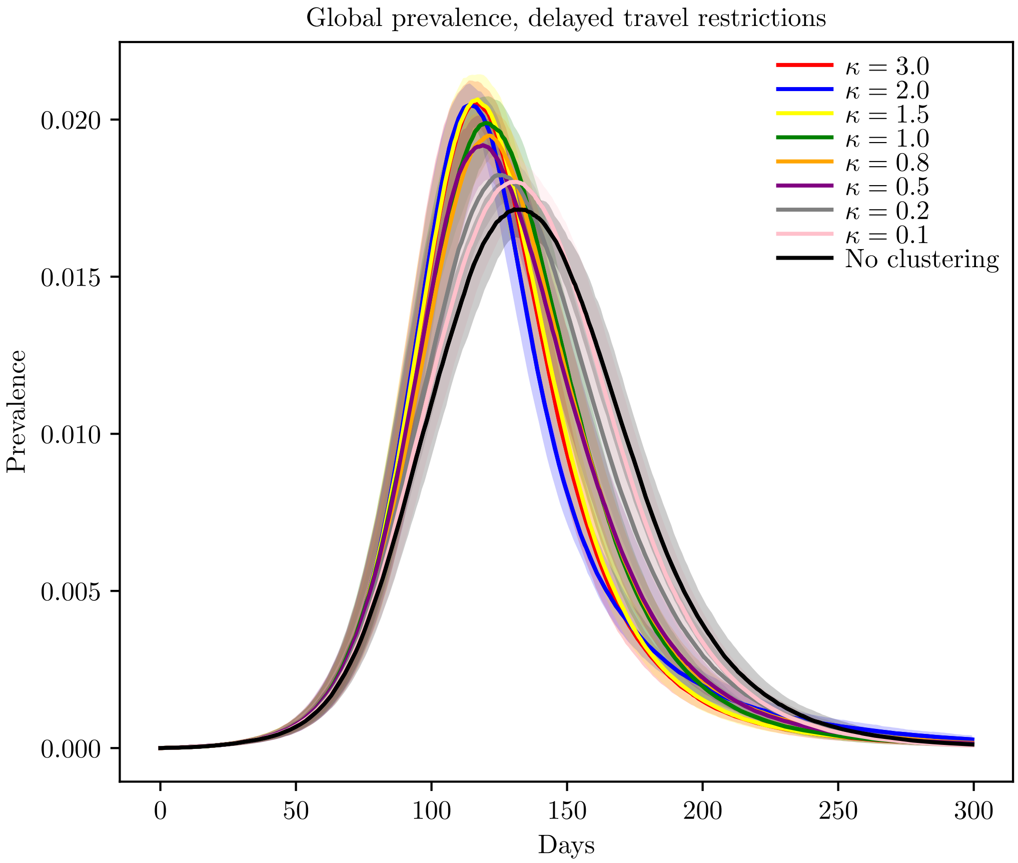

Global prevalence under delayed travel restrictions. Estimated global prevalence for the various smoothing levels with corresponding 95% confidence bands, in the setting with delay in implementation of travel restrictions.

S6 Fig.

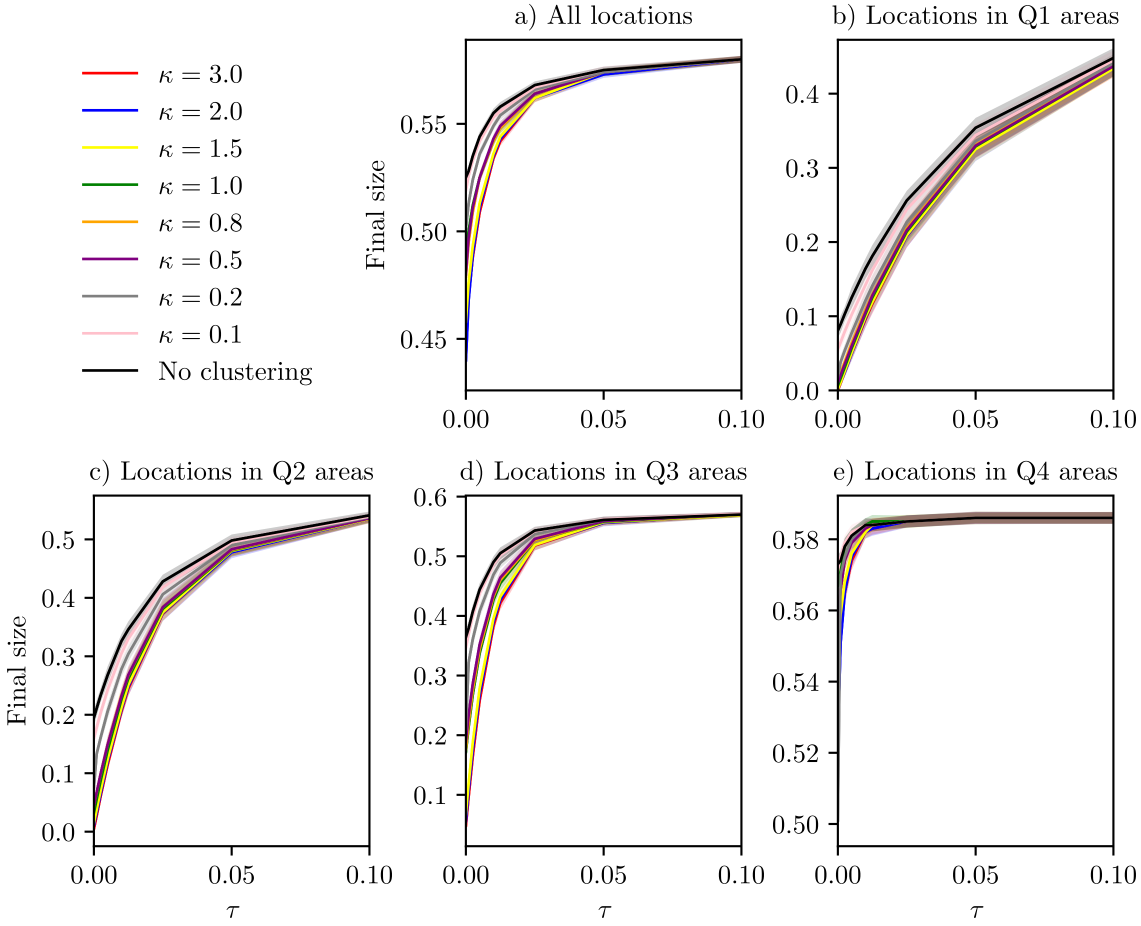

Final size versus travel ratio with varying travel duration. Final size versus τ for various clustering levels, κ, when the length of stay for non-commuting travellers varies, with corresponding 95% confidence bands. a) All locations. b) Locations with population size smaller than the 25% quantile. c) Locations with population size between the 25% and 50% quantile. d) Locations with population size between the 50% quantile and the 75% quantile. e) Locations with population size larger than the 75% quantile.

S7 Fig.

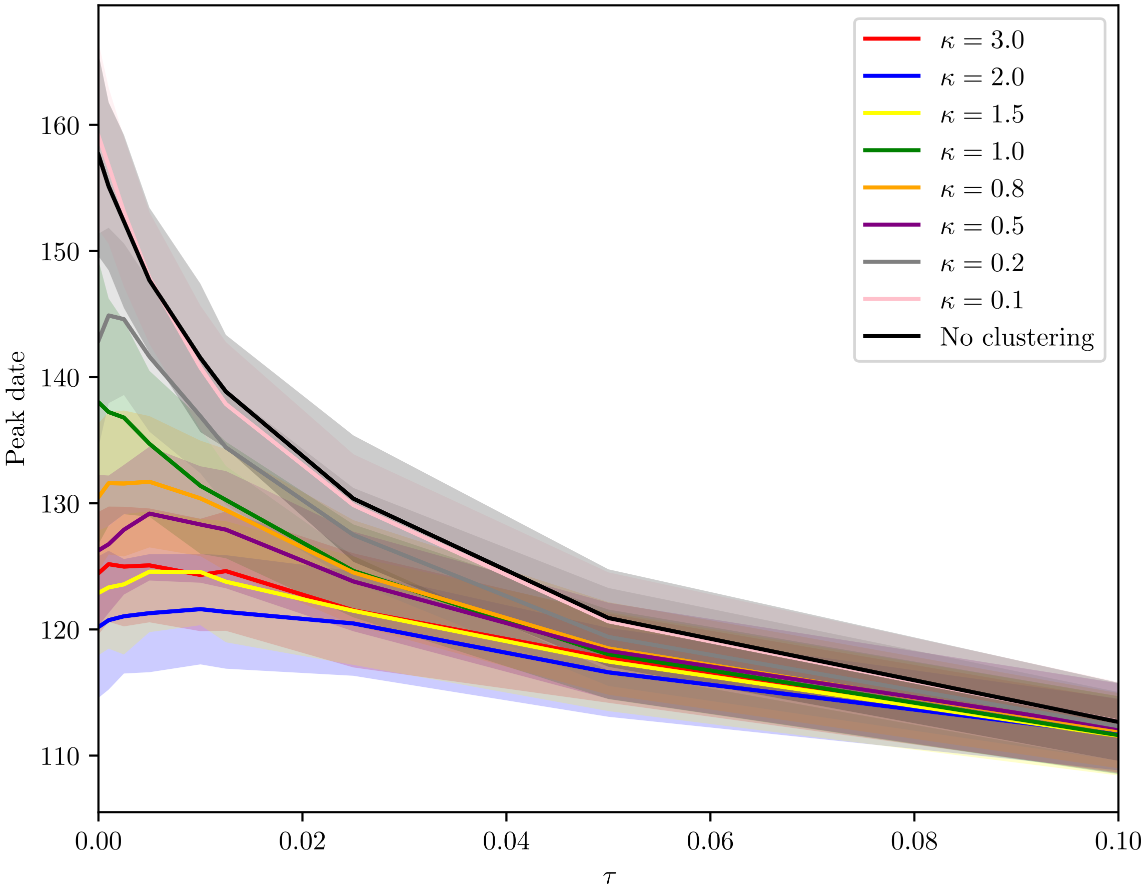

Peak dates versus travel ratio with varying travel duration. Peak date versus τ for various clustering levels, κ, when the length of stay for non-commuting travellers varies, with corresponding 95% confidence bands.

S8 Fig.

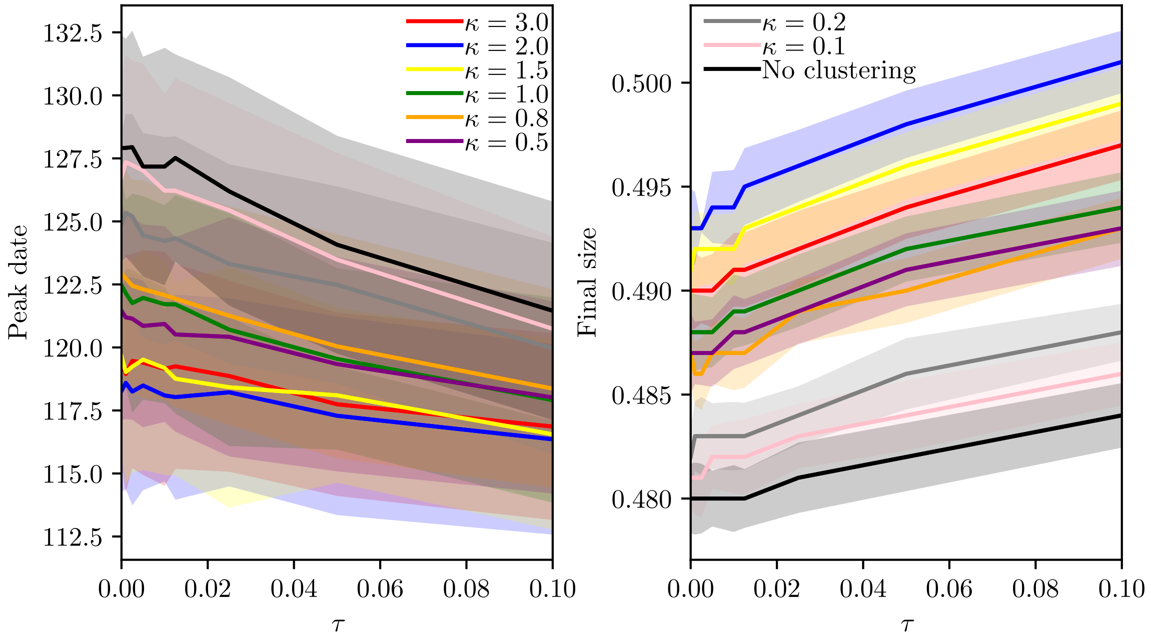

Final size and peak date with travel ban targeting infectious symptomatic. Final size and peak date versus τ for various clustering levels, κ, with only travel restrictions for the infectious symptomatic, with corresponding 95% confidence bands.

S9 Fig.

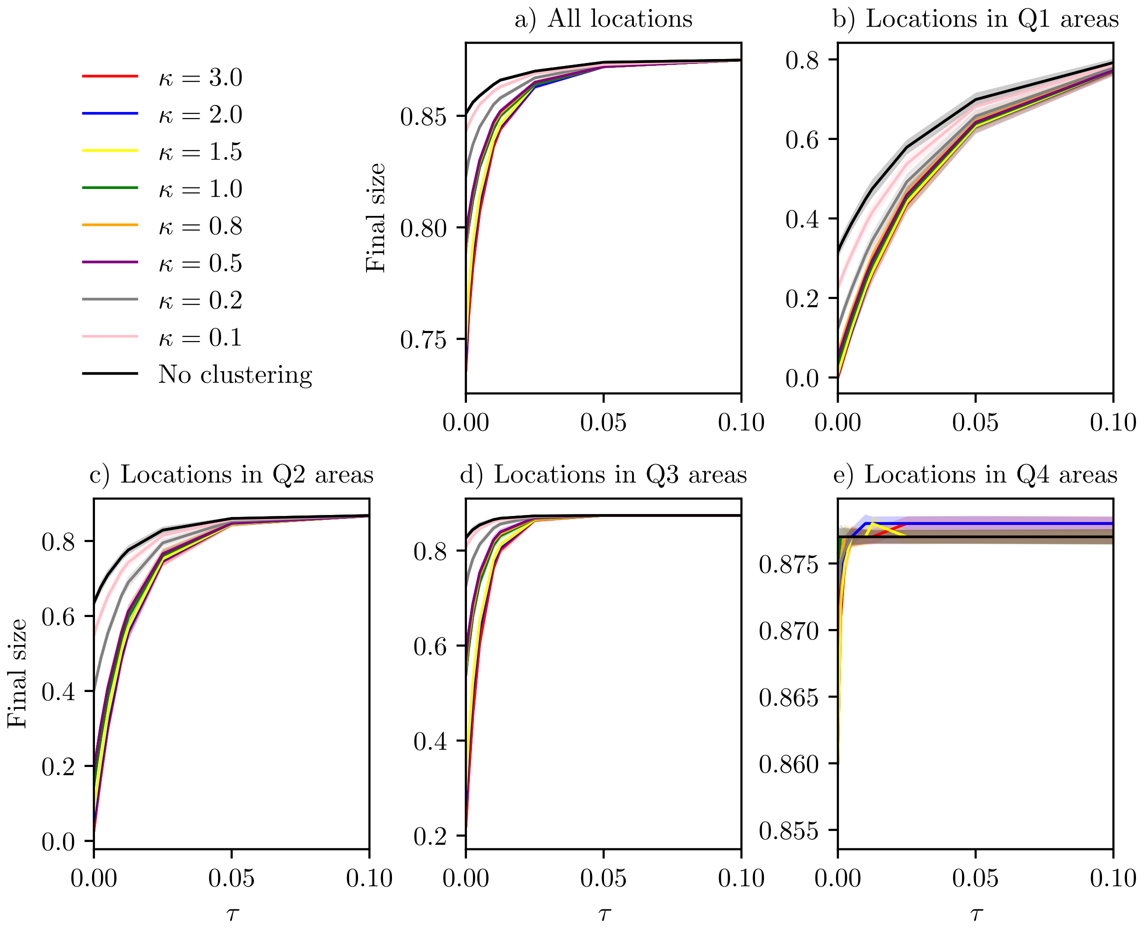

Final size versus travel ratio with alternative disease parameters. Final size versus τ for various clustering levels, κ, for the alternative disease parameters, with corresponding 95% confidence bands. a) All locations. b) Locations with population size smaller than the 25% quantile. c) Locations with population size between the 25% and 50% quantile. d) Locations with population size between the 50% quantile and the 75% quantile. e) Locations with population size larger than the 75% quantile.

S10 Fig.

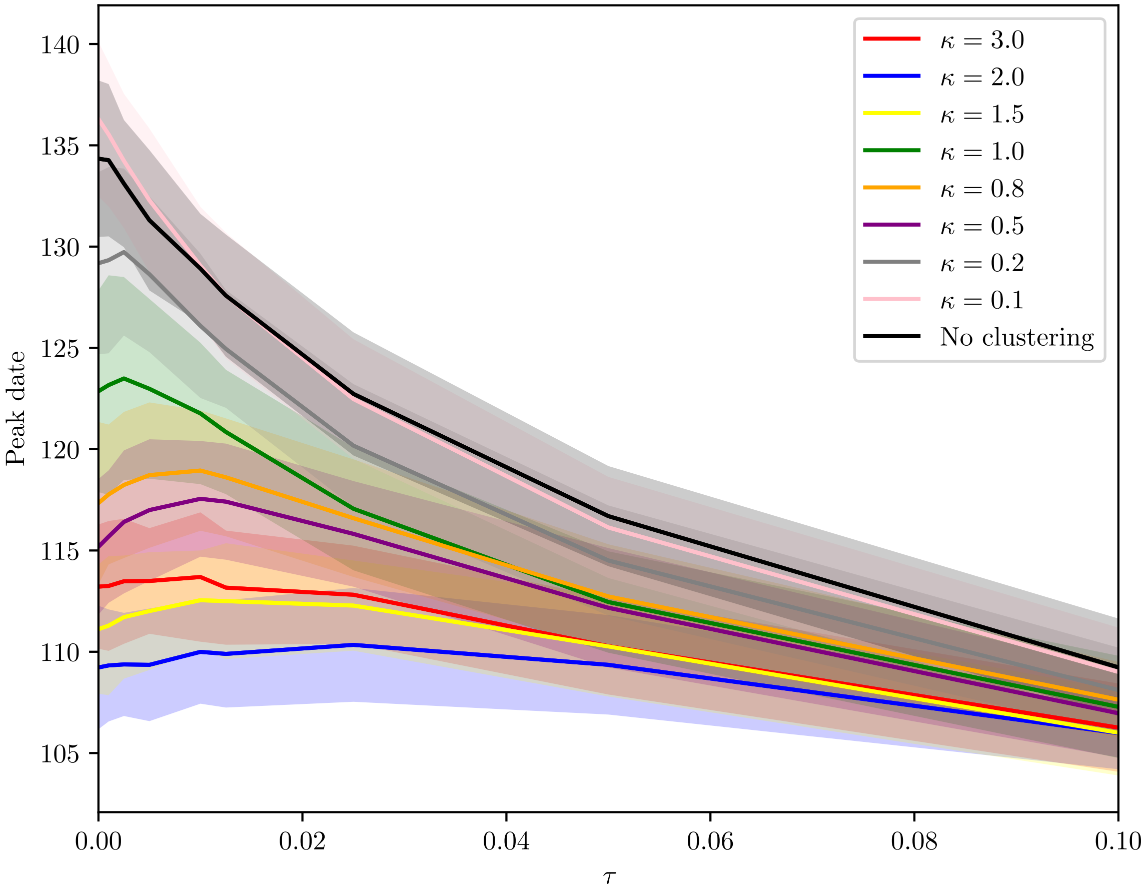

Peak date versus travel ratio for alternative disease parameters. Peak date versus τ for various clustering levels, κ, with corresponding 95% confidence bands. The results are for the alternative disease parameters.

S11 Fig.

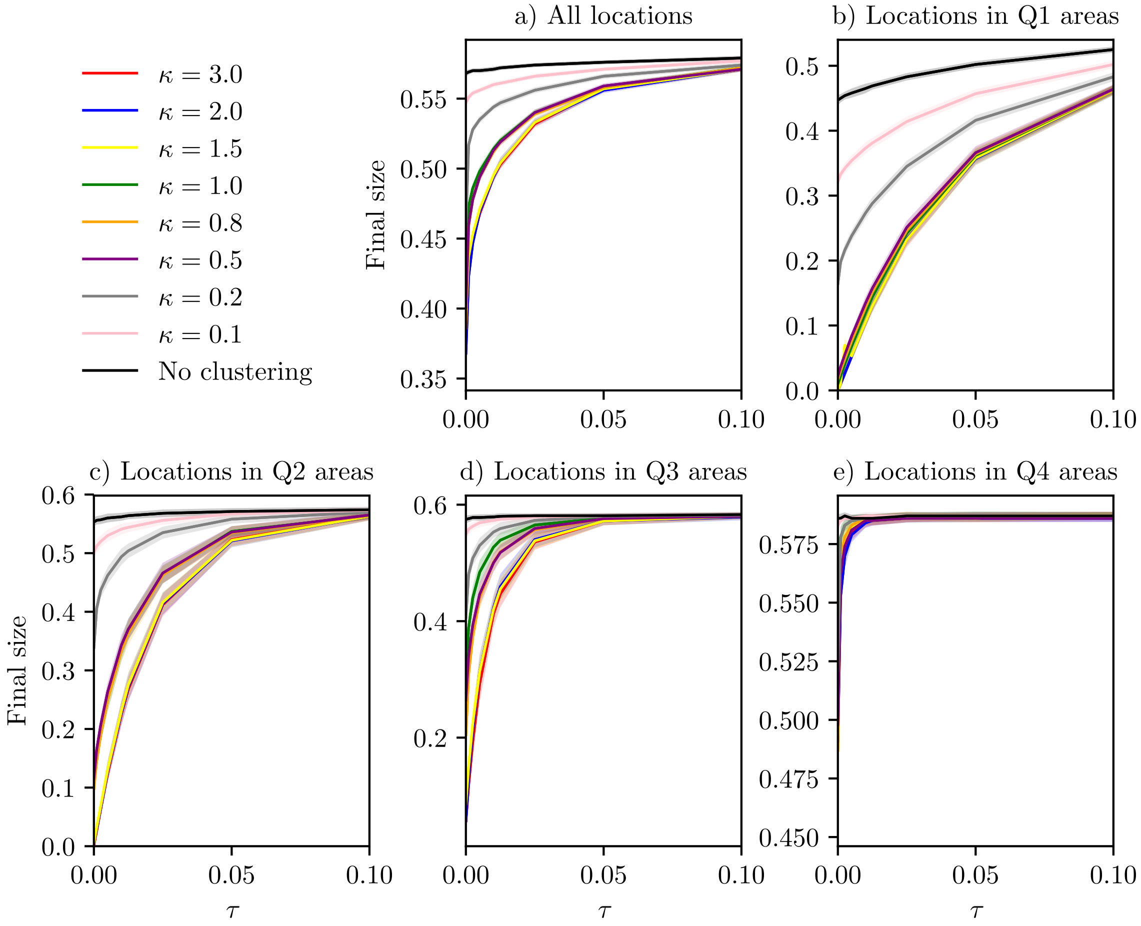

Final size versus travel ratio for the UK based country. Final size versus τ for various clustering levels, κ, with corresponding 95% confidence bands. The results are in the countries based on data from the United Kingdom. a) All locations. b) Locations with population size smaller than the 25% quantile. c) Locations with population size between the 25% and 50% quantile. d) Locations with population size between the 50% quantile and the 75% quantile. e) Locations with population size larger than the 75% quantile.

S12 Fig.

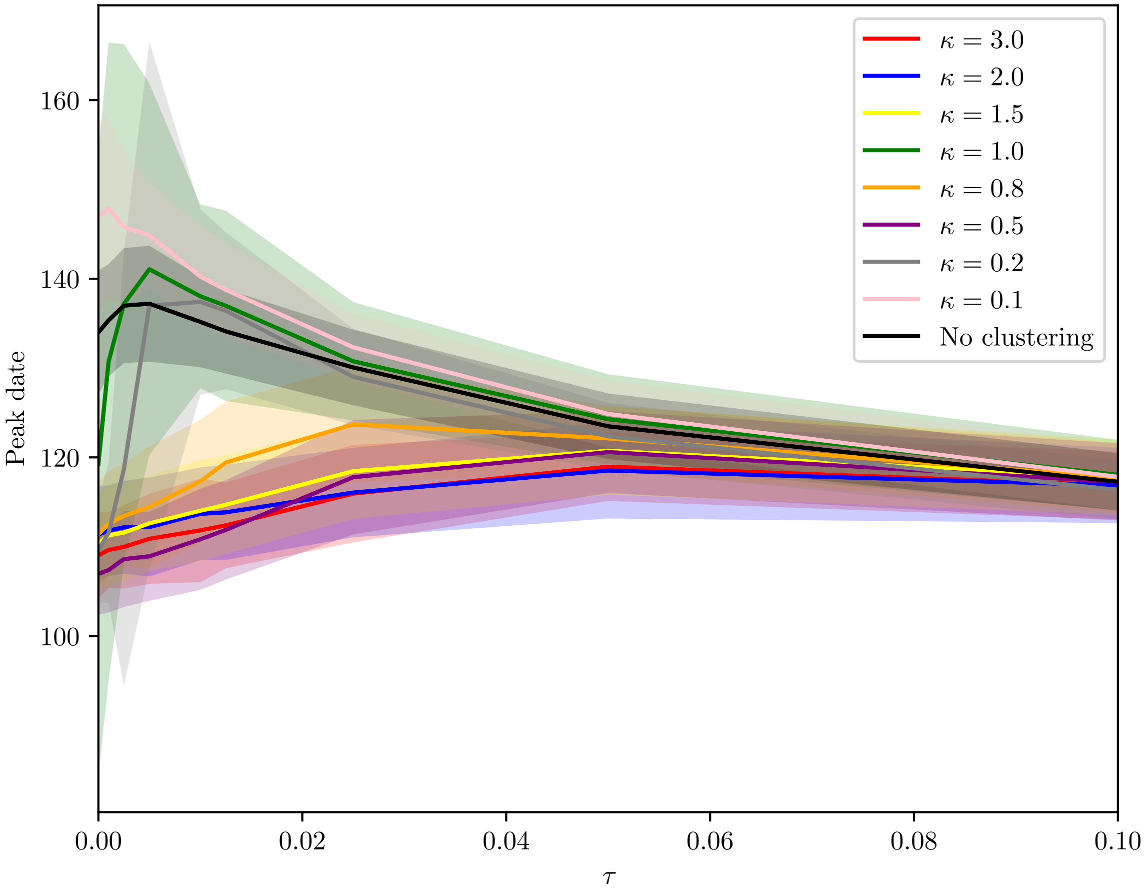

Peak date versus travel ratio for the UK based country. Peak date versus τ for various clustering levels, κ, with corresponding 95% confidence bands. The results are in the country based on data from the United Kingdom.

S13 Fig.

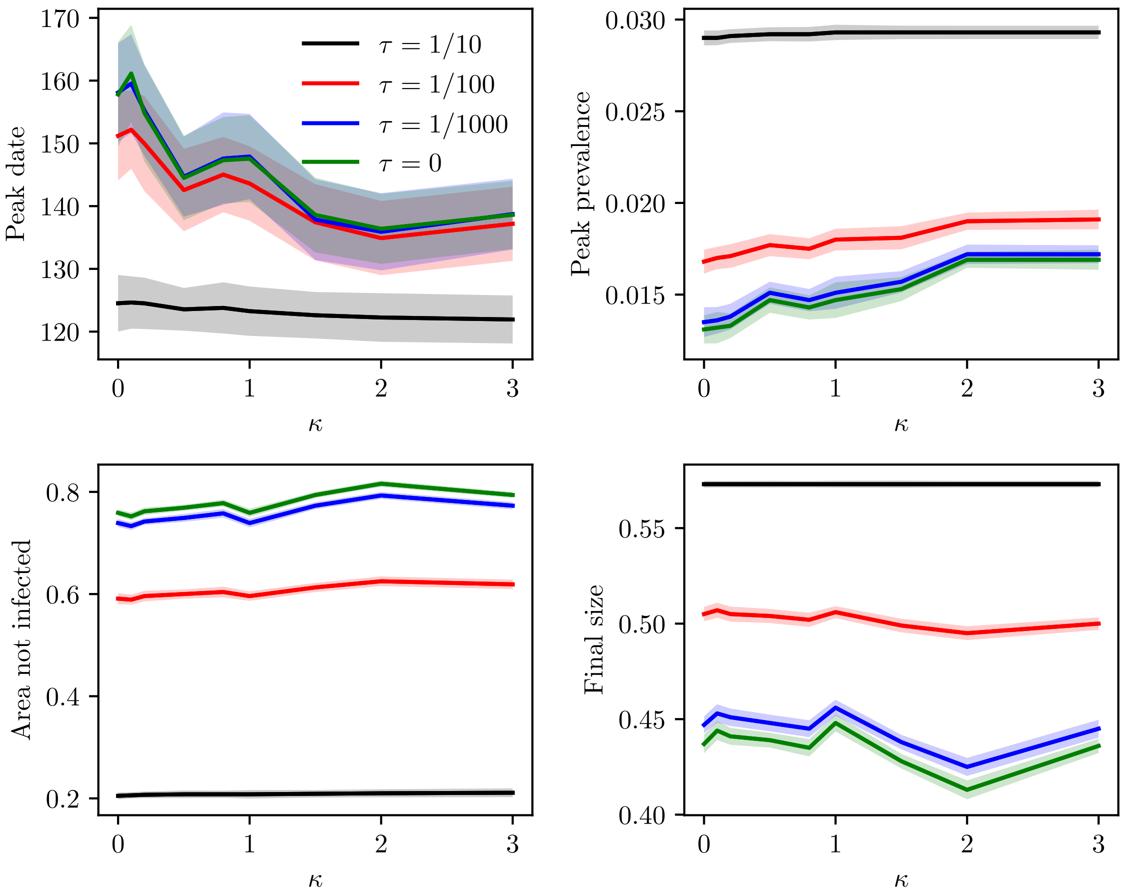

Sensitivity analysis (halving distance parameter): Peak dates, peak prevalence, area not infected and final size Peak dates for the global mean prevalence curve, peak prevalence, mean area not infected and mean final size as a function of clustering, with 95% confidence bands, when the distance parameter of the gravity law was halved. The lines correspond to the baseline scenario, 90% travel restrictions, 99% travel restrictions and 100% travel restrictions. Top left: peak date. Top right: peak prevalence. Bottom left: area not infected. Bottom right: final size.

S14 Fig.

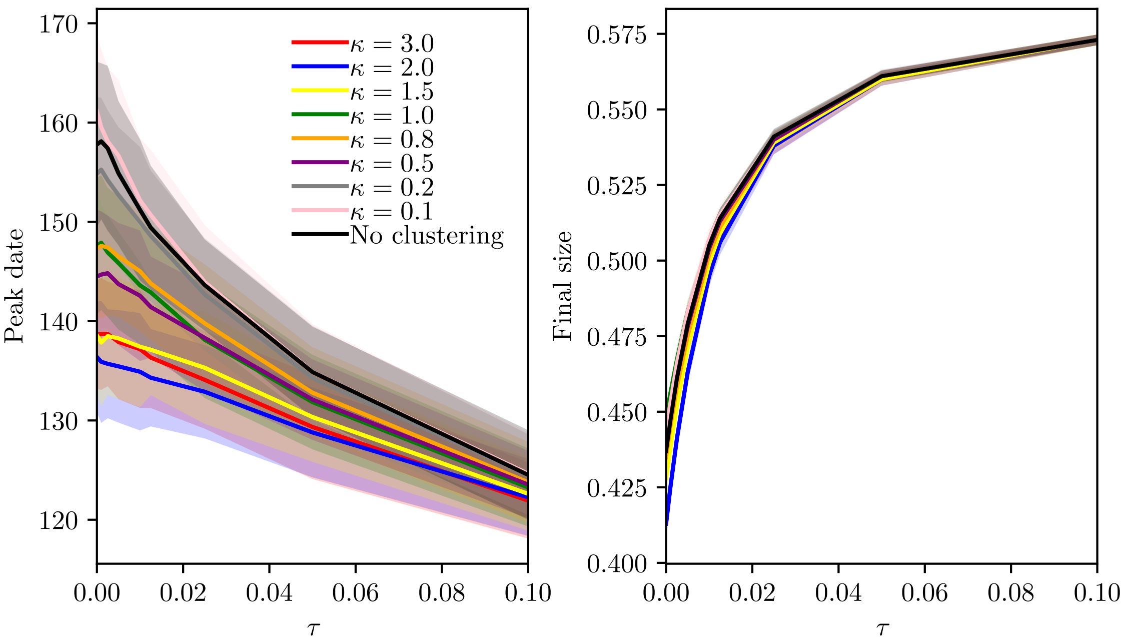

Sensitivity analysis (halving distance parameter): Final size and peak date Final size and peak date versus τ for various clustering levels, κ, with corresponding 95% confidence bands, when the distance parameter of the gravity law was halved.

S15 Fig.

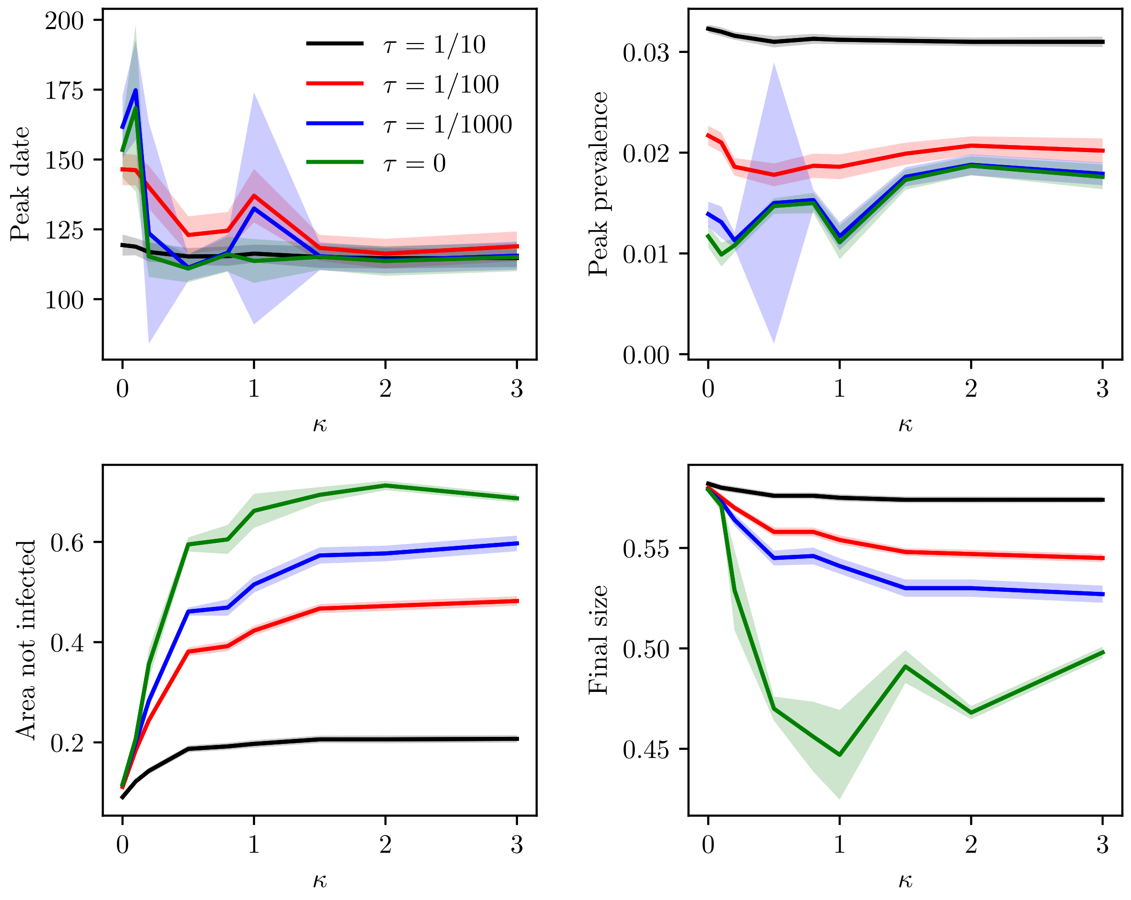

Sensitivity analysis (doubling distance parameter): Peak dates, peak prevalence, area not infected and final size Peak dates for the global mean prevalence curve, peak prevalence, mean area not infected and mean final size as a function of clustering, with 95% confidence bands, when the distance parameter of the gravity law was doubled. The lines correspond to the baseline scenario, 90% travel restrictions, 99% travel restrictions and 100% travel restrictions. Top left: peak date. Top right: peak prevalence. Bottom left: area not infected. Bottom right: final size.

S16 Fig.

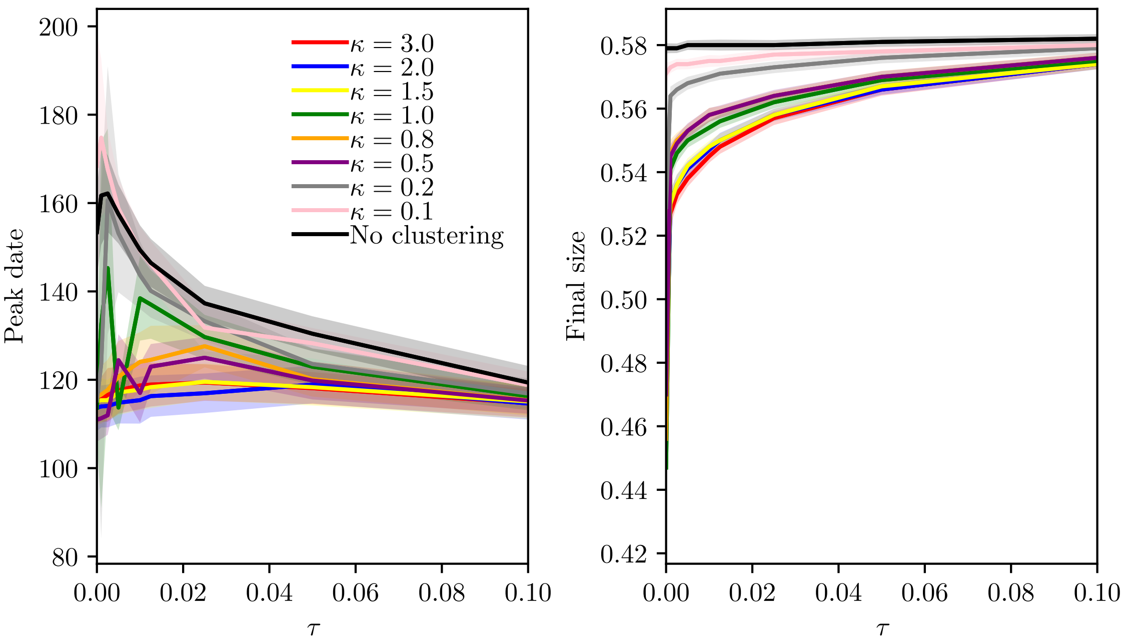

Sensitivity analysis (doubling distance parameter): Final size and peak date
Final size and peak date versus τ for various clustering levels, κ, with corresponding 95% confidence bands, when the distance parameter of the gravity law was doubled.

S17 Fig.

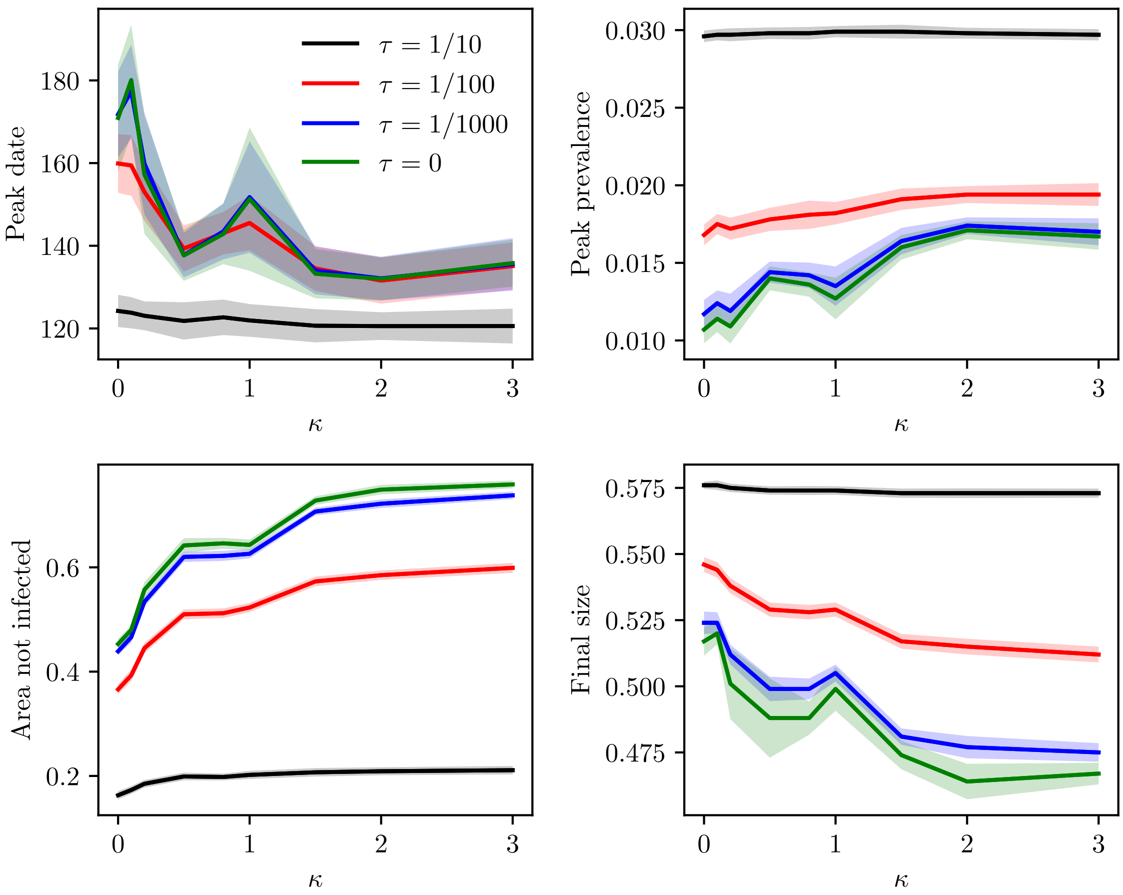

Sensitivity analysis (halving destination population parameter): Peak dates, peak prevalence, area not infected and final size Peak dates for the global mean prevalence curve, peak prevalence, mean area not infected and mean final size as a function of clustering, with 95% confidence bands, when the destination population parameter of the gravity law was halved. The lines correspond to the baseline scenario, 90% travel restrictions, 99% travel restrictions and 100% travel restrictions. Top left: peak date. Top right: peak prevalence. Bottom left: area not infected. Bottom right: final size.

S18 Fig.

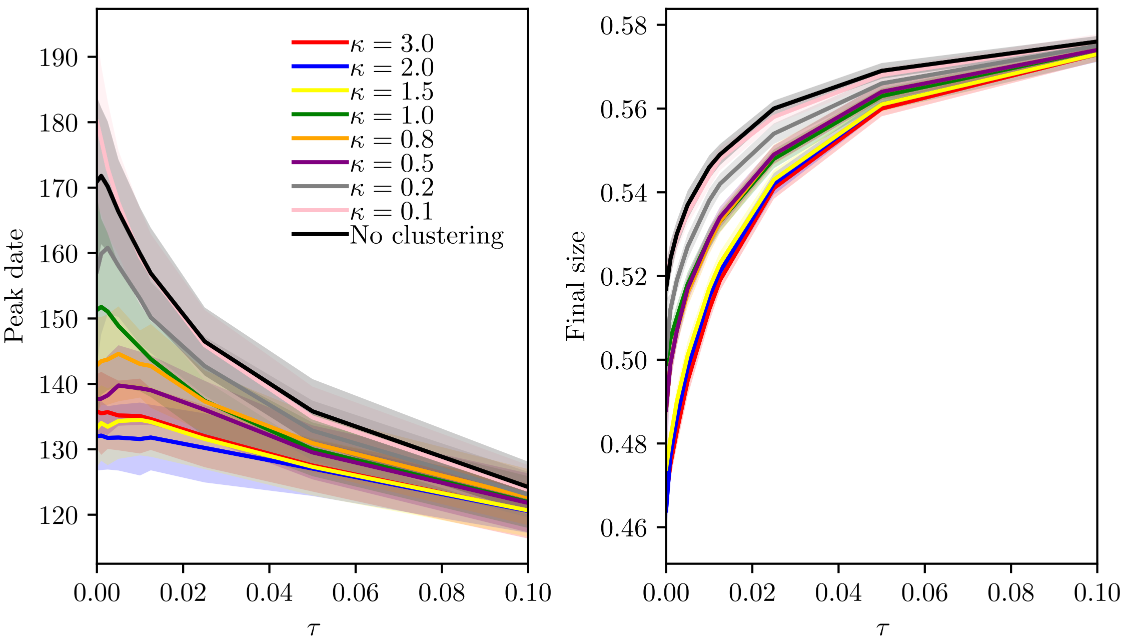

Sensitivity analysis (halving destination population parameter): Final size and peak date Final size and peak date versus τ for various clustering levels, κ, with corresponding 95% confidence bands, when the destination population parameter of the gravity law was halved.

S19 Fig.

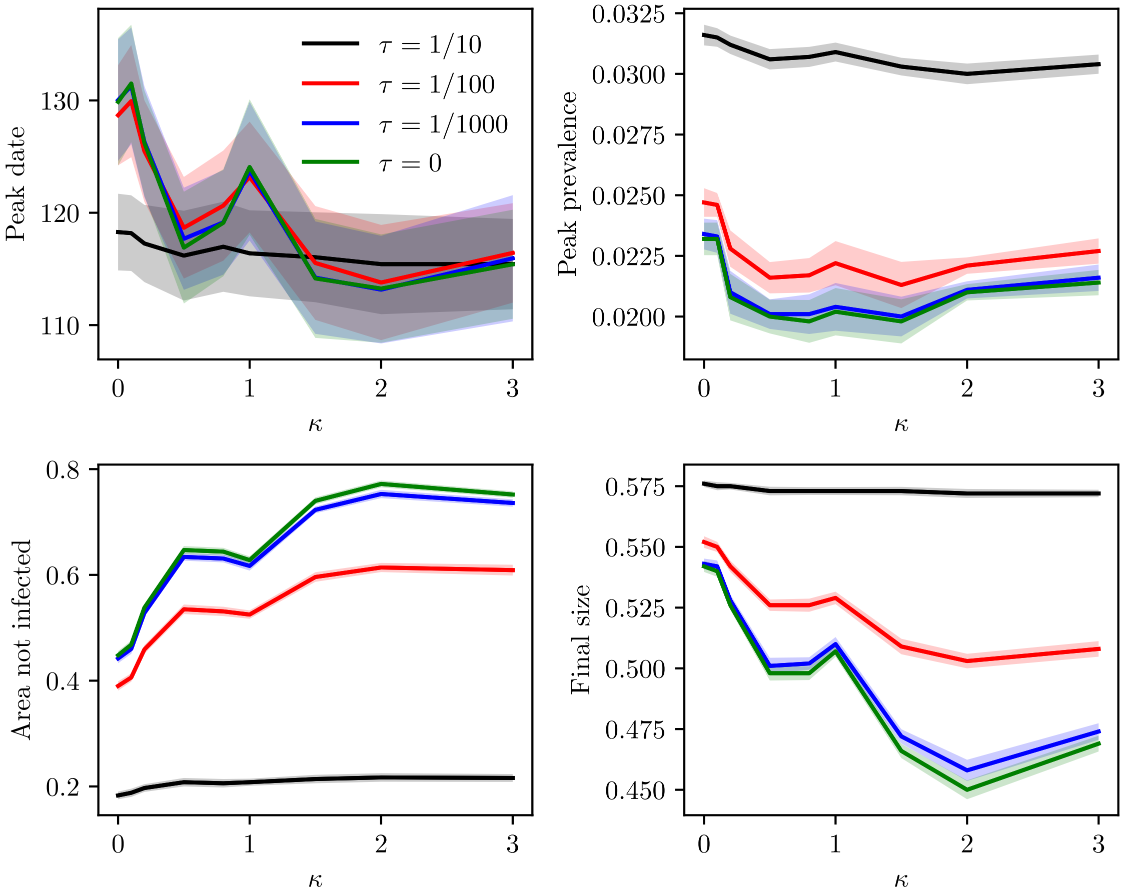

Sensitivity analysis (doubling destination population parameter): Peak dates, peak prevalence, area not infected and final size Peak dates for the global mean prevalence curve, peak prevalence, mean area not infected and mean final size as a function of clustering, with 95% confidence bands, when the destination population parameter of the gravity law was doubled. The lines correspond to the baseline scenario, 90% travel restrictions, 99% travel restrictions and 100% travel restrictions. Top left: peak date. Top right: peak prevalence. Bottom left: area not infected. Bottom right: final size.

S20 Fig.

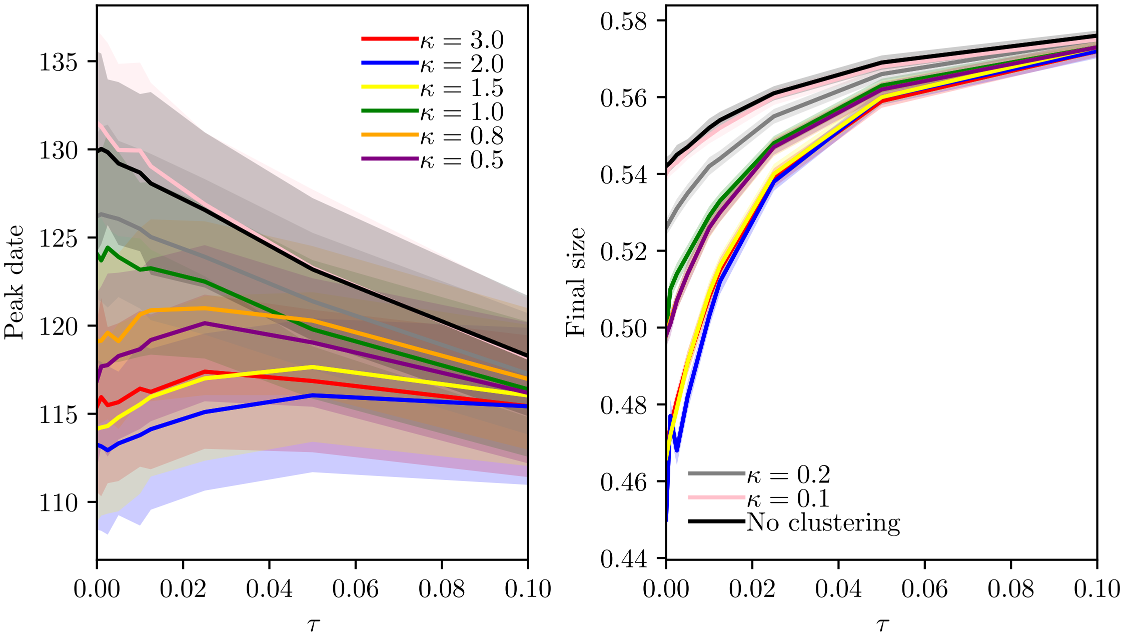

Sensitivity analysis (doubling destination population parameter): Final size and peak date Final size and peak date versus τ for various clustering levels, κ, with corresponding 95% confidence bands, when the destination population parameter of the gravity law was doubled.

S21 Fig.

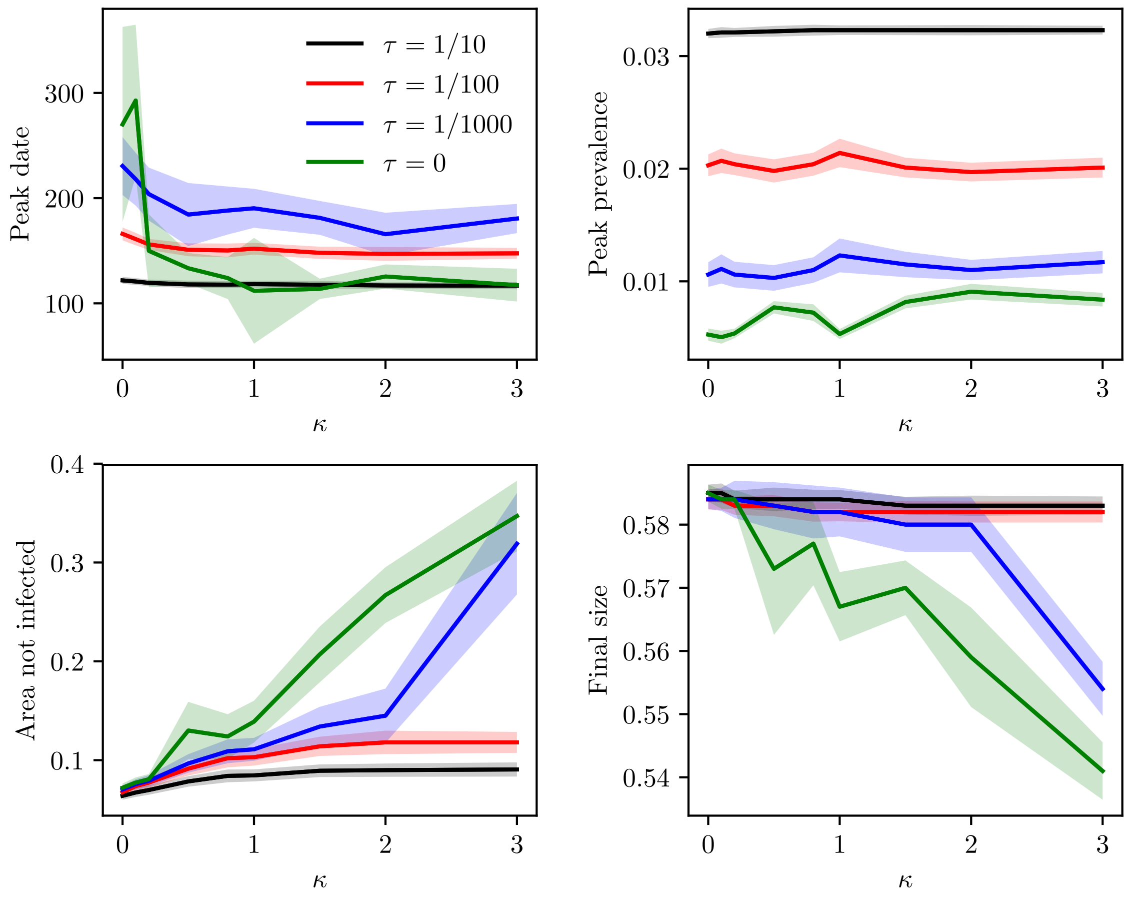

Peak dates, peak prevalence, area not infected and final size, exponential distance function Peak dates for the global mean prevalence curve, peak prevalence, mean area not infected and mean final size as a function of clustering, with 95% confidence bands, with an exponential function of distance in the gravity law. The lines correspond to the baseline scenario, 90% travel restrictions, 99% travel restrictions and 100% travel restrictions. Top left: peak date. Top right: peak prevalence. Bottom left: area not infected. Bottom right: final size.

S22 Fig.

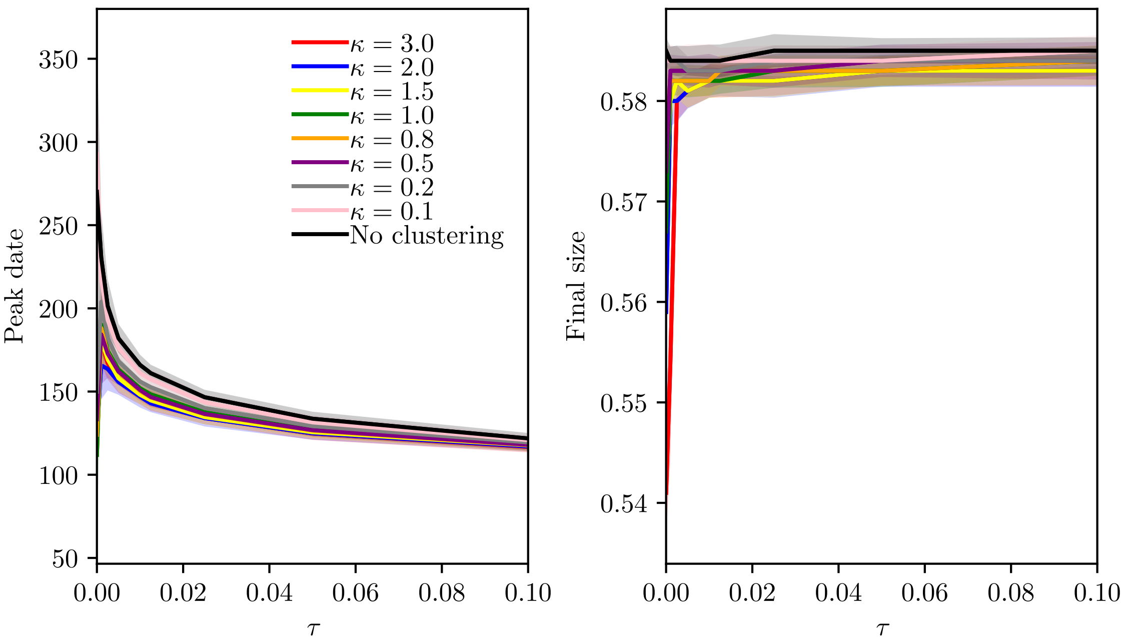

Final size and peak date with exponential distance function Final size and peak date versus τ for various clustering levels, κ, with corresponding 95% confidence bands, with an exponential function of distance in the gravity law.

S23 Fig.

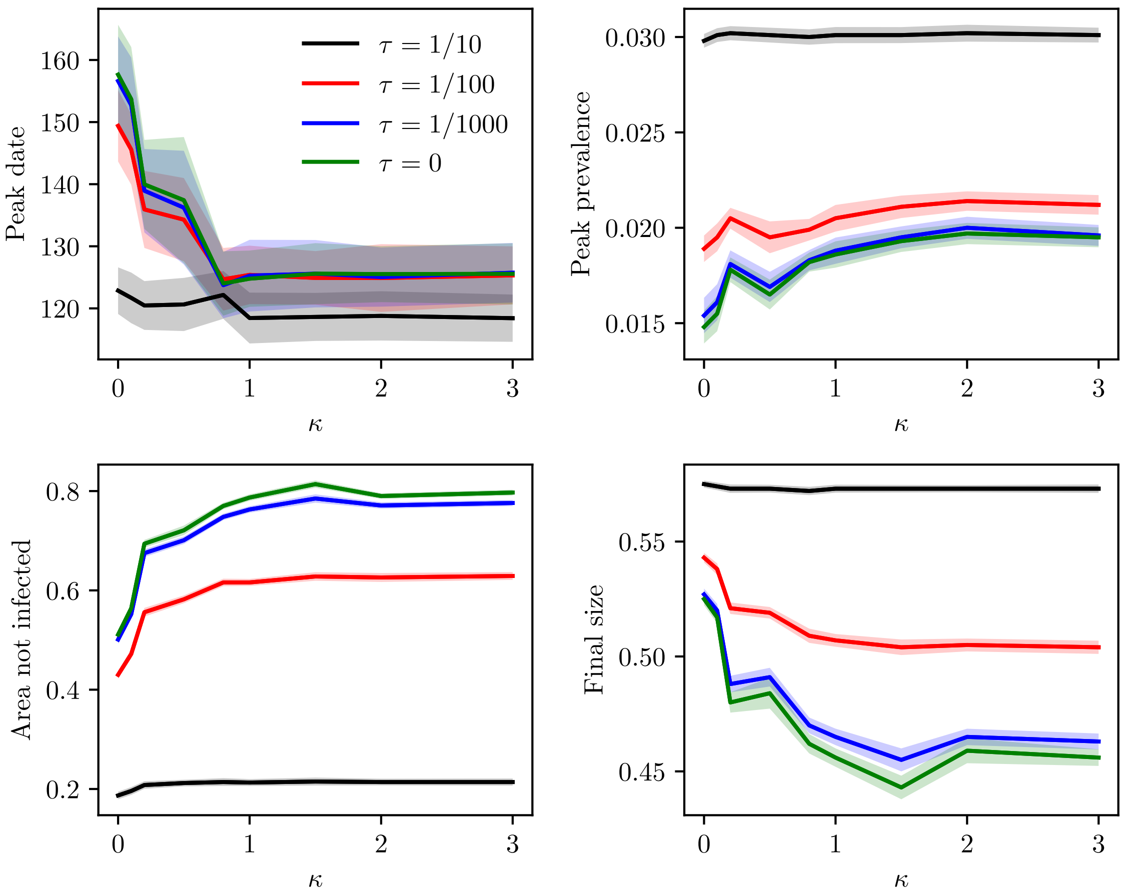

Peak dates, peak prevalence, area not infected and final size, range parameter 10.0 in the covariance function Peak dates for the global mean prevalence curve, peak prevalence, mean area not infected and mean final size as a function of clustering, with 95% confidence bands, when the range parameter of the Matérn covariance function was increased from 5.0 to 10.0. The lines correspond to the baseline scenario, 90% travel restrictions, 99% travel restrictions and 100% travel restrictions. Top left: peak date. Top right: peak prevalence. Bottom left: area not infected. Bottom right: final size.

S24 Fig.

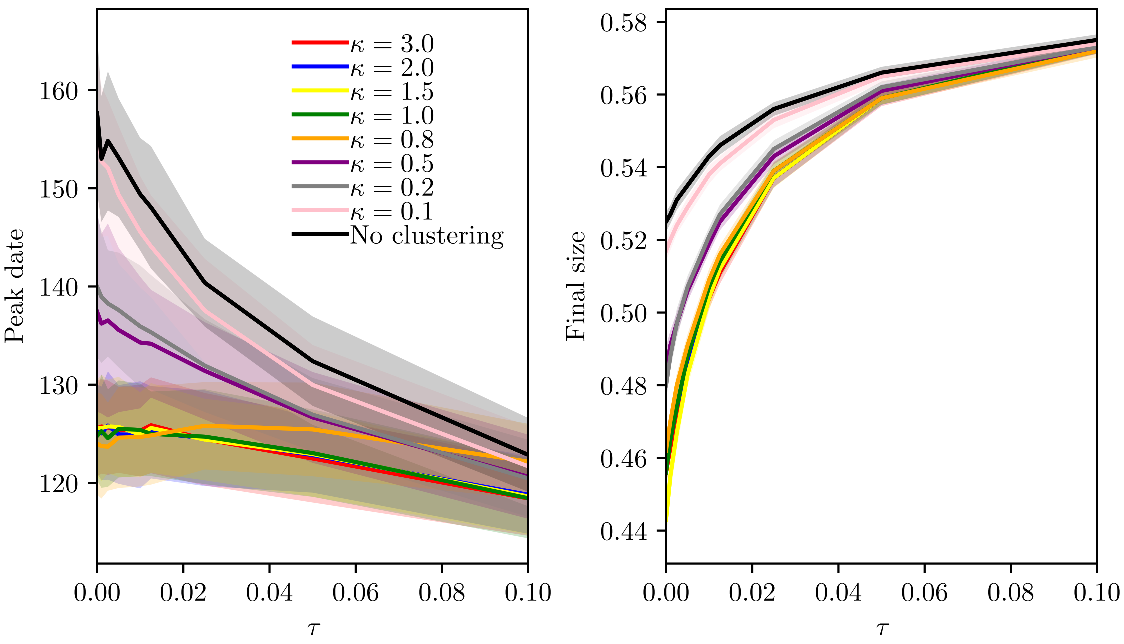

Final size and peak date with range parameter 10.0 in the covariance function
Final size and peak date versus τ for various clustering levels, κ, with corresponding 95% confidence bands, when the range parameter of the Matérn covariance function was increased from 5.0 to 10.0.

S25 Fig.

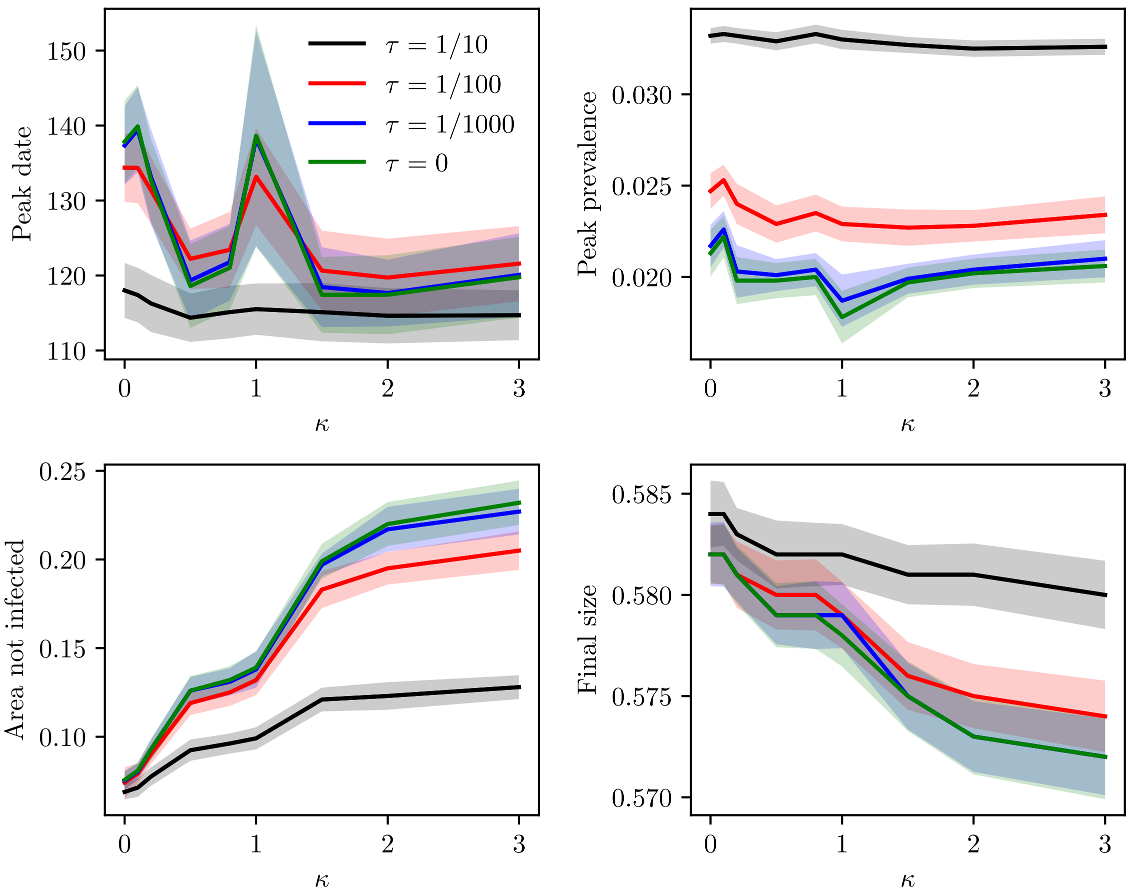

Peak dates, peak prevalence, area not infected and final size, when the radiation law was used to model commuting Peak dates for the global mean prevalence curve, peak prevalence, mean area not infected and mean final size as a function of clustering, with 95% confidence bands, when the commuting was implemented by the radiation law. The lines correspond to the baseline scenario, 90% travel restrictions, 99% travel restrictions and 100% travel restrictions. Top left: peak date. Top right: peak prevalence. Bottom left: area not infected. Bottom right: final size.

S26 Fig.

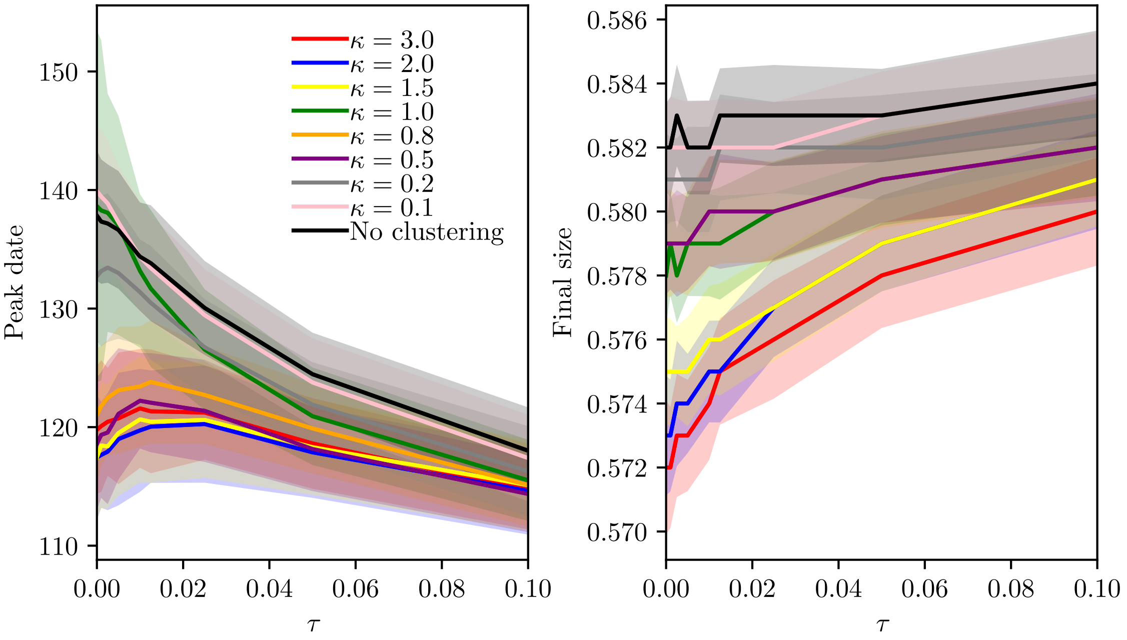

Final size and peak date when the radiation law was used to model commuting Final size and peak date versus τ for various clustering levels, κ, with corresponding 95% confidence bands, for the results where commuting was implemented by the radiation law.

S27 Fig.

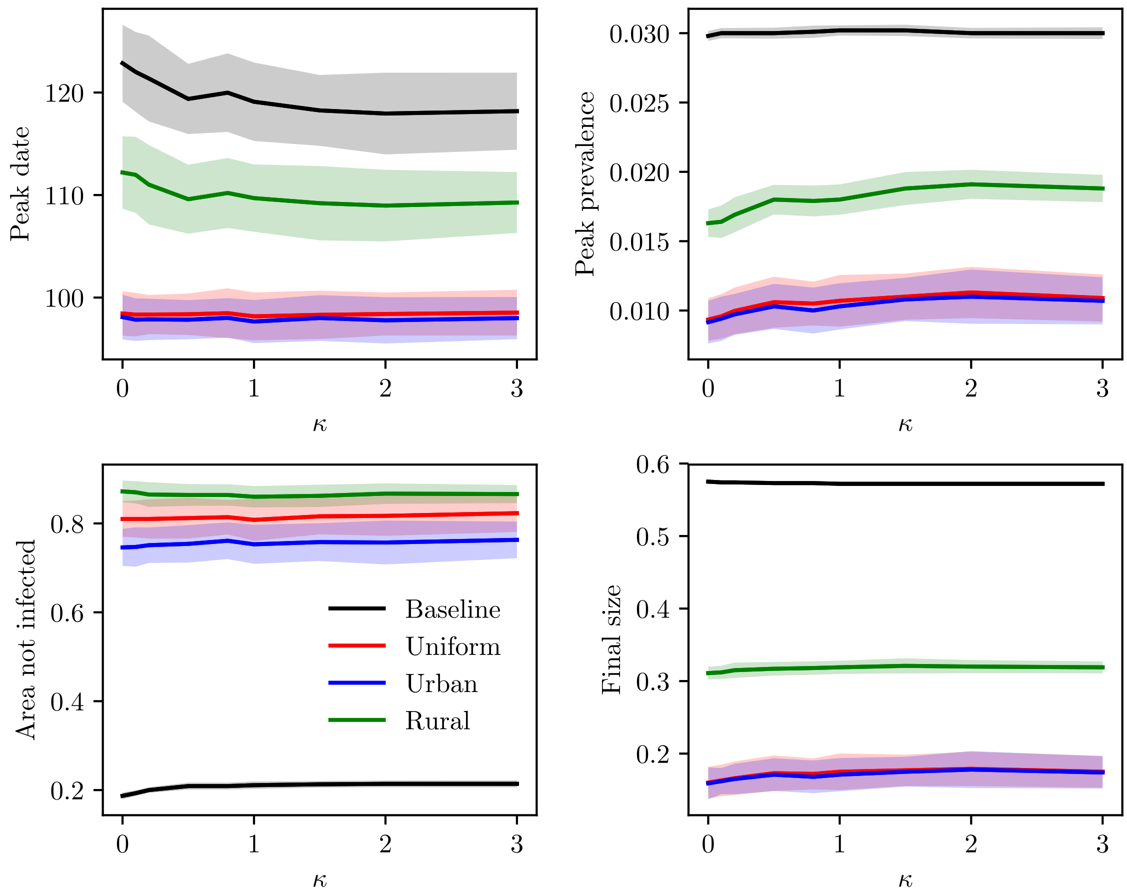

Peak dates, peak prevalence, area not infected and final size, 20% reduced infectiousness Peak dates for the global mean prevalence curve, peak prevalence, mean area not infected and mean final size as a function of clustering, with 95% confidence bands, when the assumed infectiousness of non-immune vaccinated is reduced by 20%. The lines correspond to the baseline scenario, uniform vaccination, urban vaccination and rural vaccination. Top left: peak date. Top right: peak prevalence. Bottom left: area not infected. Bottom right: final size.

S28 Fig.

Estimated a for urban and rural locations.

κ	Q1	Q2	Q3	Q4
No clustering	0.599	0.512	0.640	2.05
0.1	0.573	0.476	0.603	2.00
0.2	0.541	0.401	0.402	1.47
0.5	0.540	0.390	0.379	1.67
0.8	0.543	0.391	0.369	1.68
1.0	0.539	0.385	0.368	1.92
1.5	0.535	0.377	0.307	1.53
2.0	0.535	0.374	0.302	1.37
3.0	0.532	0.371	0.302	1.47

Estimated power a for final size $= \tau^a + b$, for different levels of clustering, for the Q1 (most rural), Q2, Q3 and Q4 (most urban) locations.

S1 Table

Estimated a for all locations.

κ	No clustering	0.1	0.2	0.5	0.8	1.0	1.5	2.0	3.0
**a**	1.31	1.27	1.14	1.03	1.02	1.07	0.919	0.862	0.891

Estimated power a for final size $= \tau^a + b$, for different levels of clustering.

S2 Table

Delayed travel restrictions.

κ	Peak day	Peak prevalence	Area not infected	Final size
No clustering	132.7 (3.5)	0.0172 (0.0004)	0.498 (0.00490)	0.527 (0.00176)
0.1	130.8 (3.5)	0.0181 (0.0005)	0.524 (0.00446)	0.525 (0.00144)
0.2	125.8 (3.2)	0.0183 (0.0004)	0.580 (0.00403)	0.513 (0.00169)
0.5	118.7 (2.7)	0.0193 (0.0004)	0.661 (0.00426)	0.495 (0.00192)
0.8	120.5 (2.6)	0.0196 (0.0004)	0.667 (0.00423)	0.494 (0.00193)
1.0	121.1 (2.8)	0.0200 (0.0004)	0.668 (0.00402)	0.498 (0.00166)
1.5	116.8 (2.5)	0.0207 (0.0004)	0.739 (0.00405)	0.474 (0.00188)
2.0	115.1 (2.3)	0.0206 (0.0003)	0.753 (0.00431)	0.466 (0.00237)
3.0	116.7 (2.1)	0.0206 (0.0004)	0.758 (0.00371)	0.467 (0.00195)

Global peak day, global peak prevalence, percentage of area not infected and final sizes in the situation with a delay in the implementation of the travel restrictions. Standard deviations are given in parenthesis.

S3 Table
