# Supplementary figures and images for: A theoretical single-parameter model for urbanisation to study infectious disease spread and interventions
Source: PLoS Comput Biol. 2019 Mar 7;15(3):e1006879. doi: 10.1371/journal.pcbi.1006879 (PMC6424465; doi:10.1371/journal.pcbi.1006879)

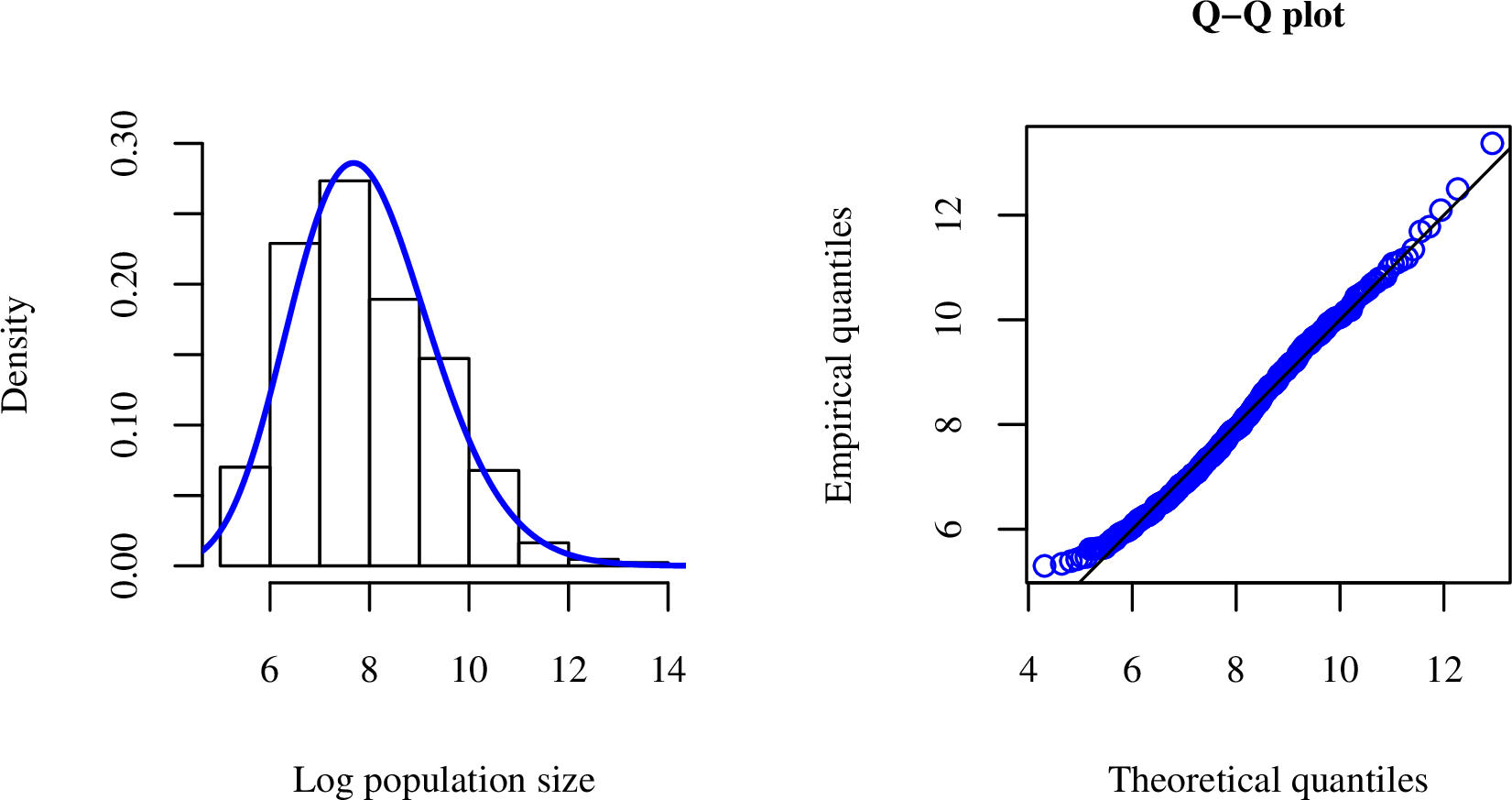

Supplement: S1 Fig — (TIF) [file pcbi.1006879.s002.tif]

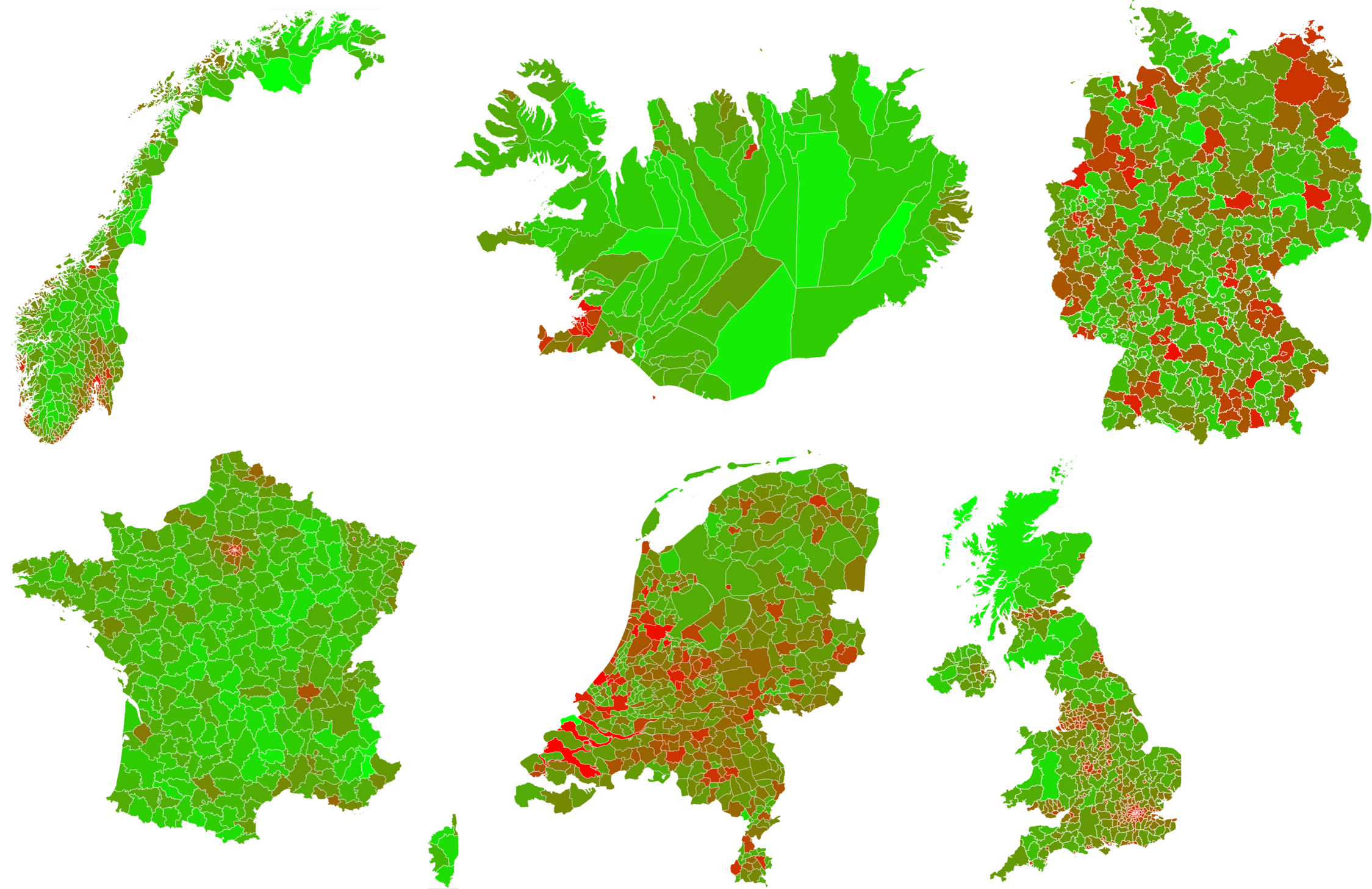

Supplement: S2 Fig — Population density in administrative units in Norway, Iceland, Germany, France, Netherlands and United Kingdom. (TIF) [file pcbi.1006879.s003.tif]

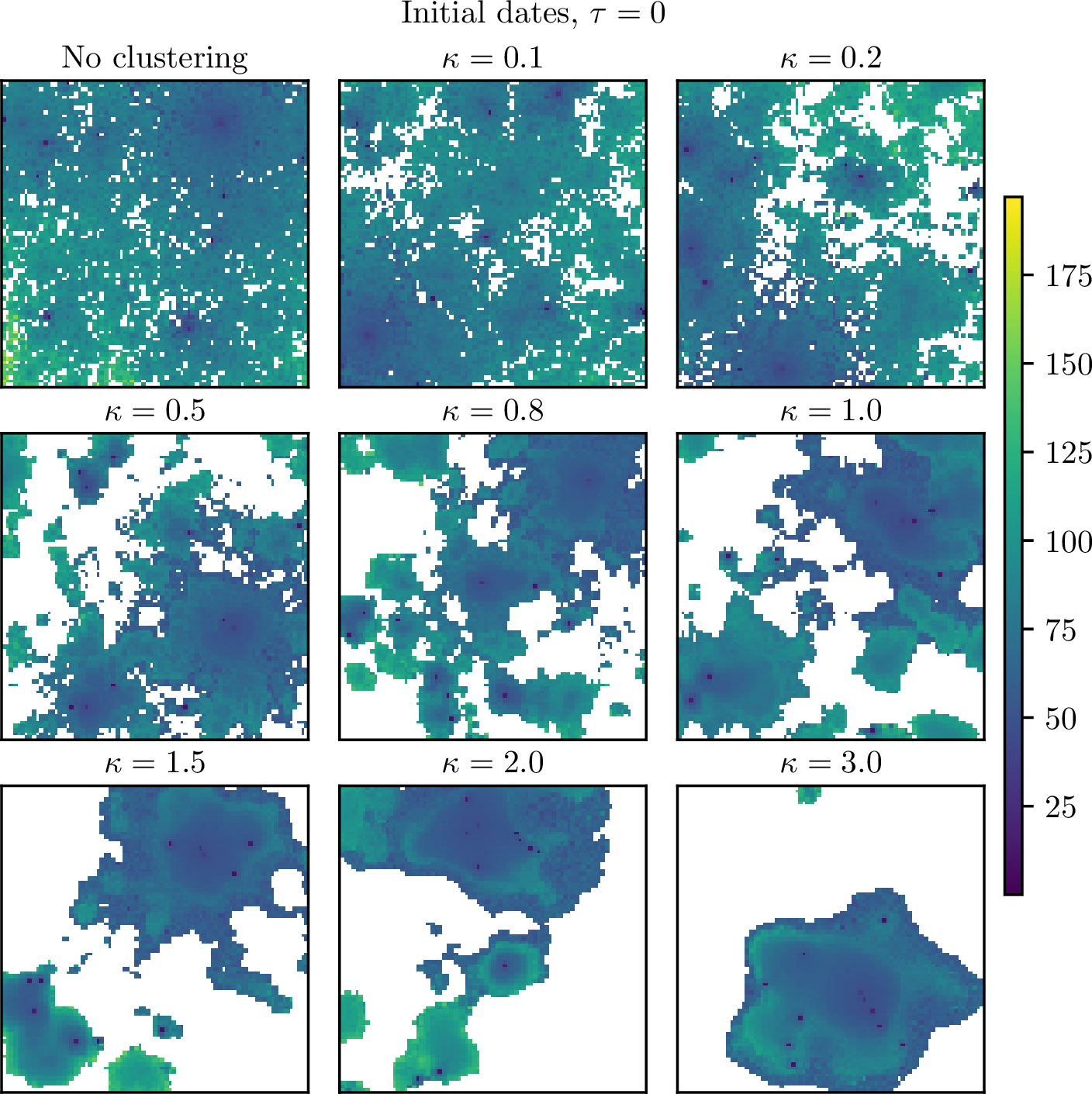

Supplement: S3 Fig — Initial dates for infection when τ = 0. These are averages over the simulations where an epidemic occurred in the respective block units. The white locations never experienced the epidemic. Upper left: No clustering. Upper center: κ = 0.1. Upper right: κ = 0.2. Middle left: κ = 0.5. Middle center: κ = 0.8. Middle right: κ = 1.0. Bottom left: κ = 1.5. Bottom center: κ = 2.0. Bottom right: κ = 3.0. (TIF) [file pcbi.1006879.s004.tif]

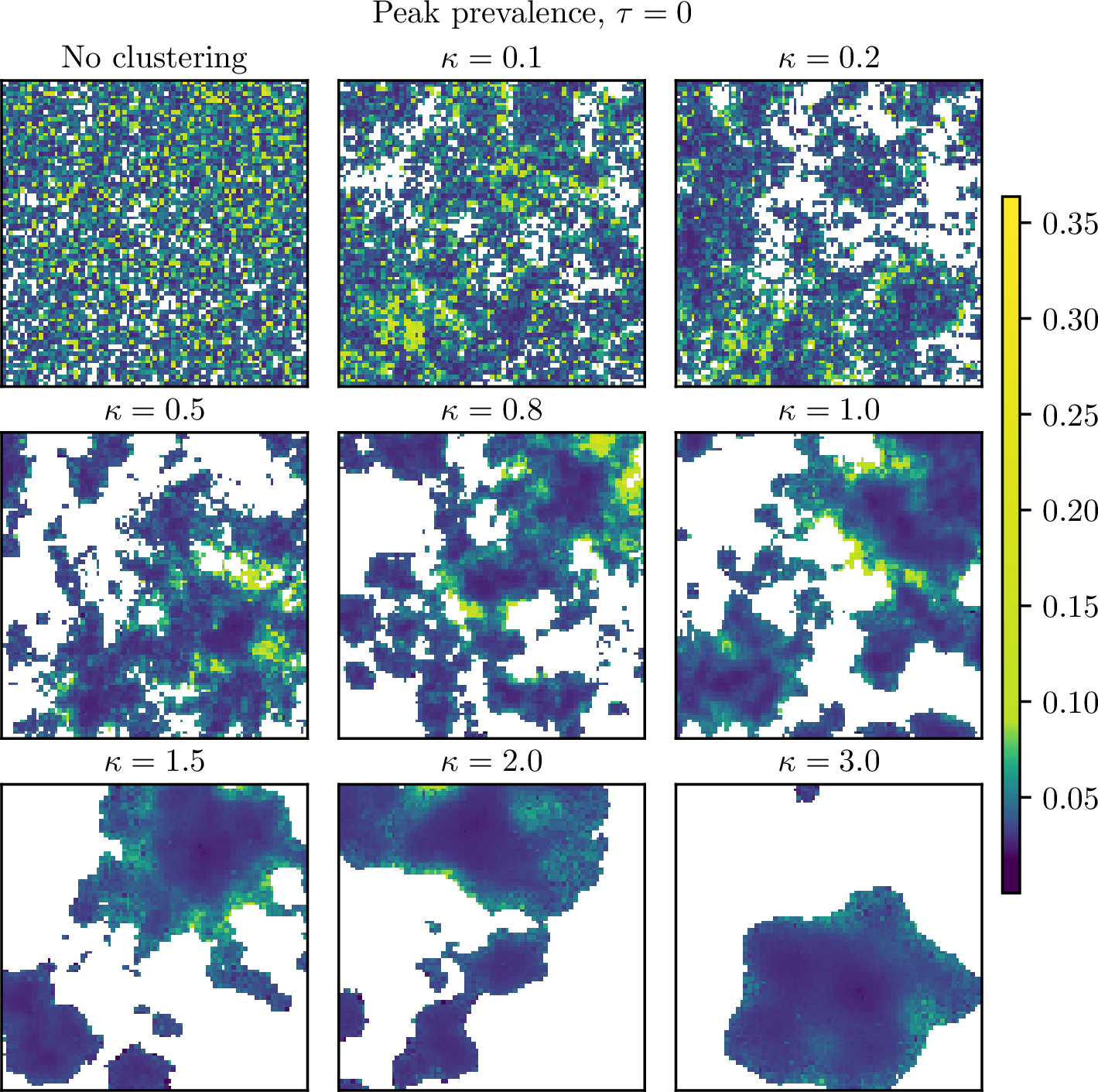

Supplement: S4 Fig — Peak prevalence for τ = 0. These are averages over the simulations where an epidemic occurred in the respective block units. The white locations never experienced the epidemic. Upper left: No clustering. Upper center: κ = 0.1. Upper right: κ = 0.2. Middle left: κ = 0.5. Middle center: κ = 0.8. Middle right: κ = 1.0. Bottom left: κ = 1.5. Bottom center: κ = 2.0. Bottom right: κ = 3.0. (TIF) [file pcbi.1006879.s005.tif]

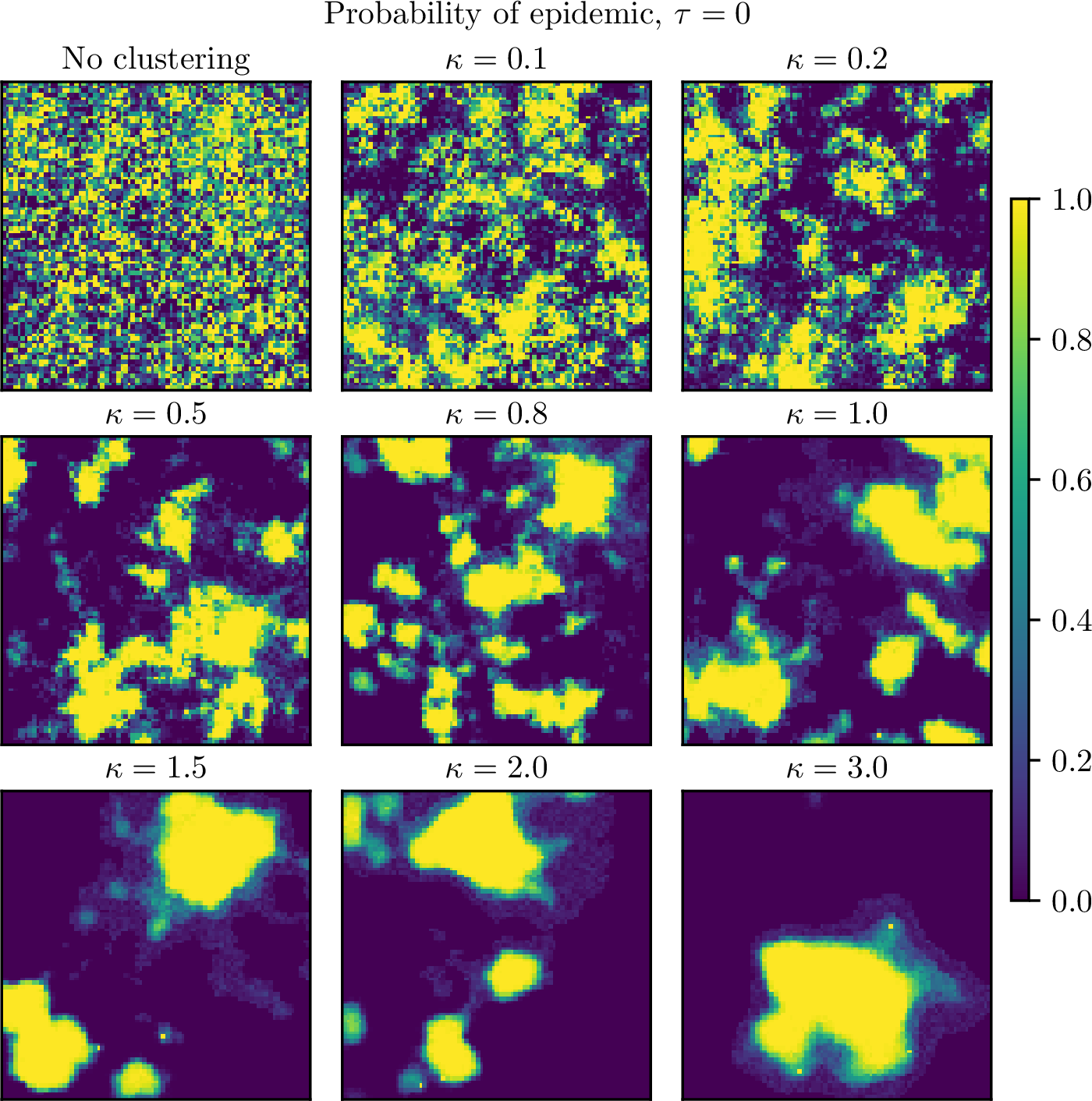

Supplement: S5 Fig — Probability of infection for τ = 0. Upper left: No clustering. Upper center: κ = 0.1. Upper right: κ = 0.2. Middle left: κ = 0.5. Middle center: κ = 0.8. Middle right: κ = 1.0. Bottom left: κ = 1.5. Bottom center: κ = 2.0. Bottom right: κ = 3.0. (TIF) [file pcbi.1006879.s006.tif]

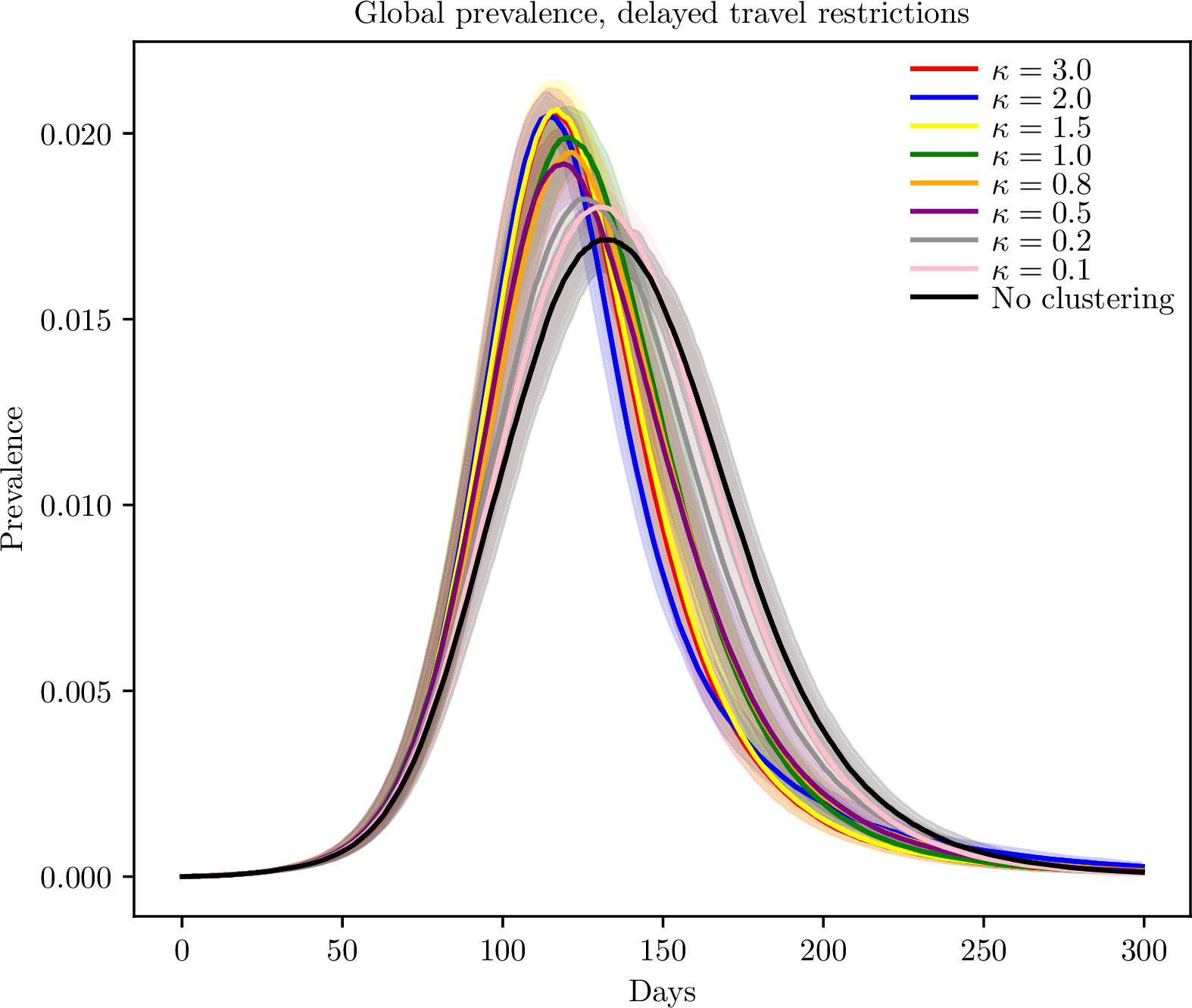

Supplement: S6 Fig — Estimated global prevalence for the various smoothing levels with corresponding 95% confidence bands, in the setting with delay in implementation of travel restrictions. (TIF) [file pcbi.1006879.s007.tif]

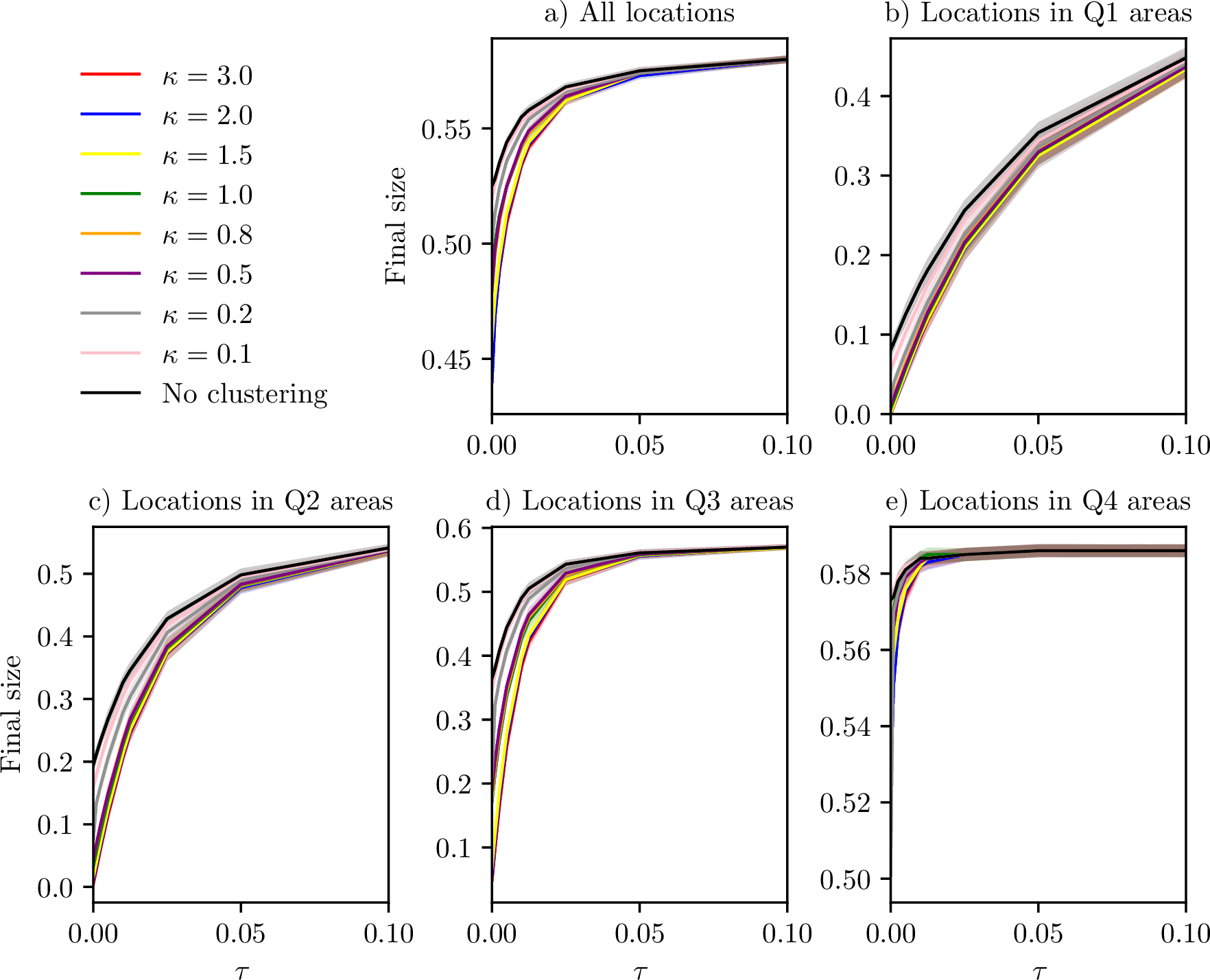

Supplement: S7 Fig — Final size versus τ for various clustering levels, κ, when the length of stay for non-commuting travellers varies, with corresponding 95% confidence bands. a) All locations. b) Locations with population size smaller than the 25% quantile. c) Locations with population size between the 25% and 50% quantile. d) Locations with population size between the 50% quantile and the 75% quantile. e) Locations with population size larger than the 75% quantile. (TIF) [file pcbi.1006879.s008.tif]

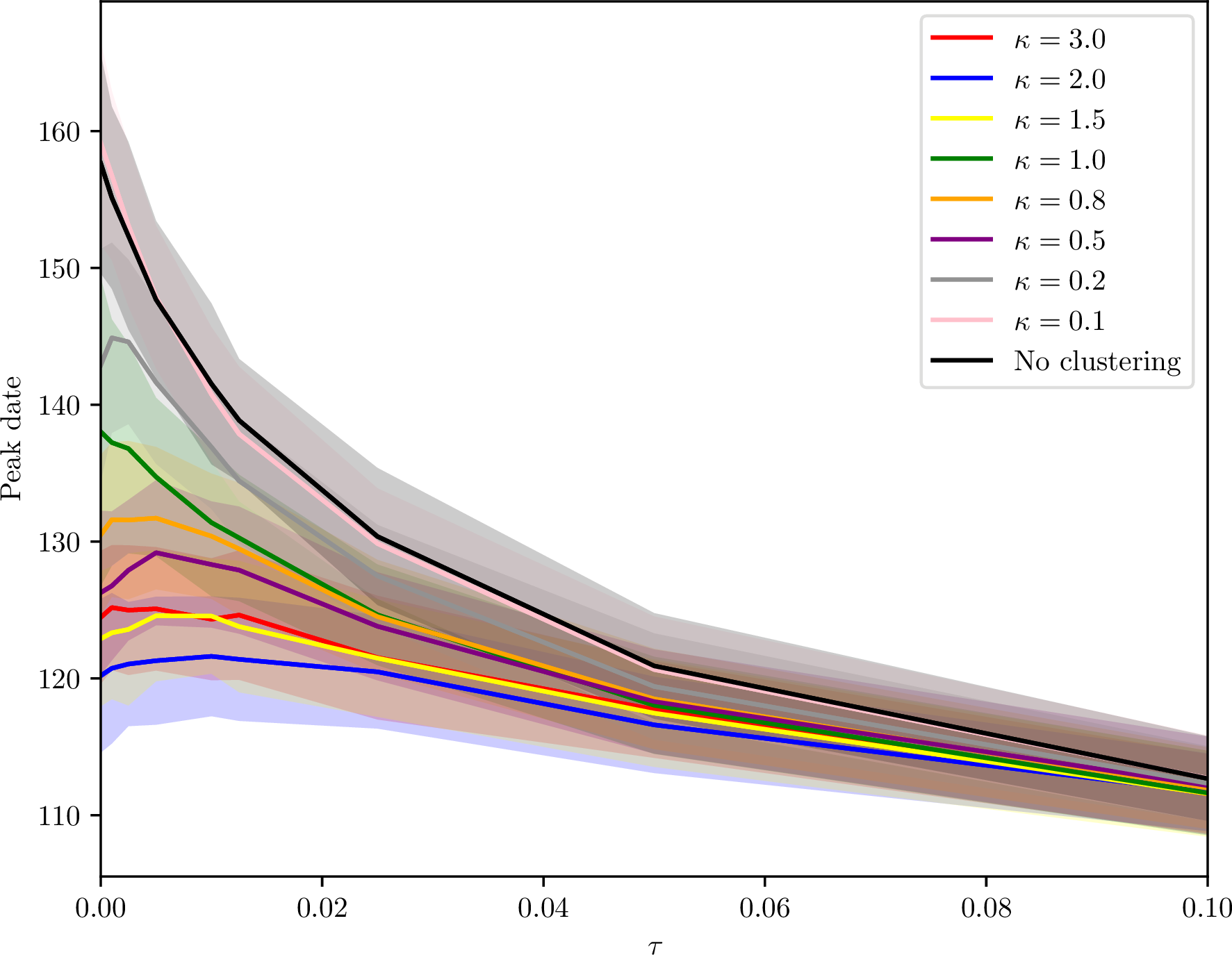

Supplement: S8 Fig — Peak date versus τ for various clustering levels, κ, when the length of stay for non-commuting travellers varies, with corresponding 95% confidence bands. (TIF) [file pcbi.1006879.s009.tif]

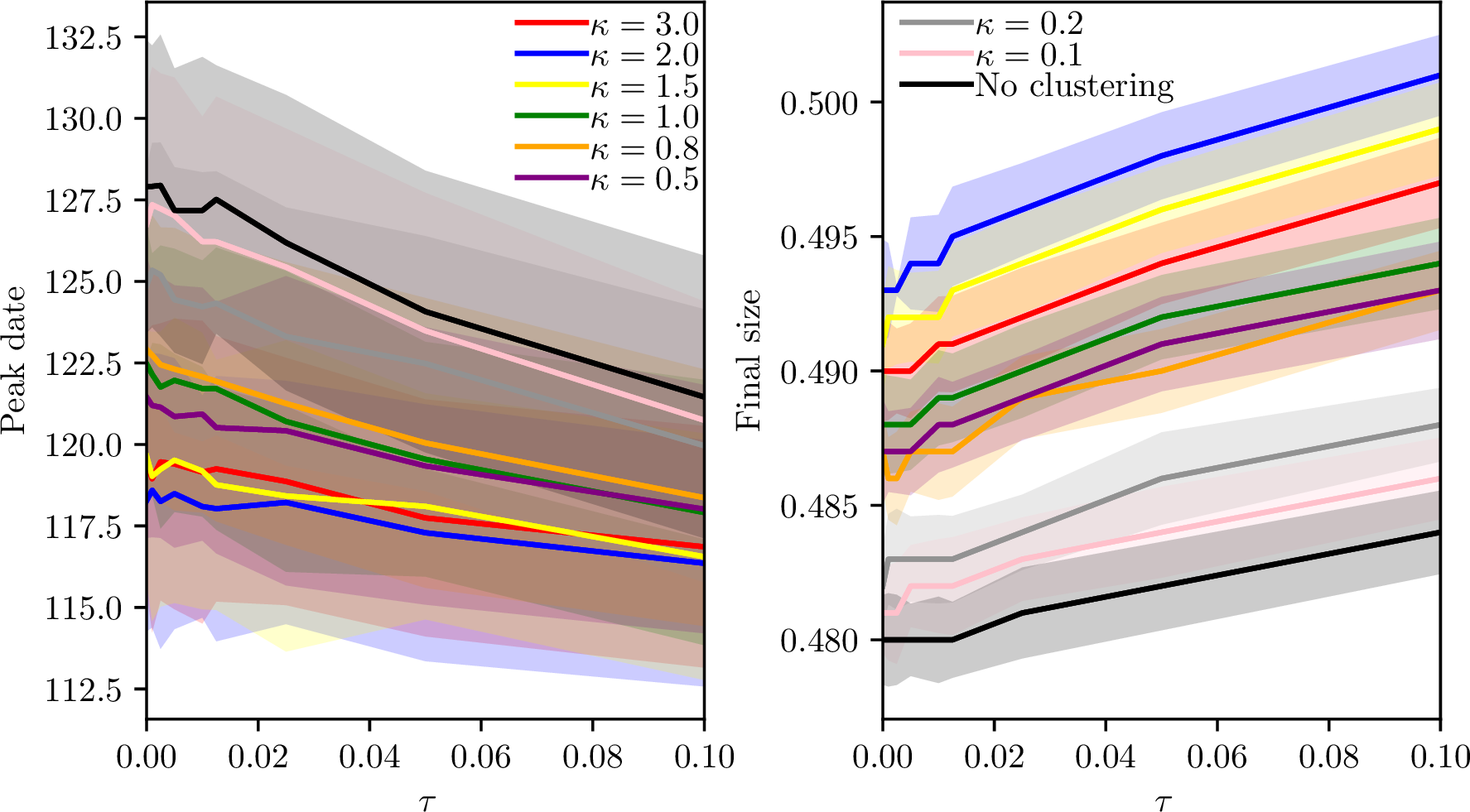

Supplement: S9 Fig — Final size and peak date versus τ for various clustering levels, κ, with only travel restrictions for the infectious symptomatic, with corresponding 95% confidence bands. (TIF) [file pcbi.1006879.s010.tif]

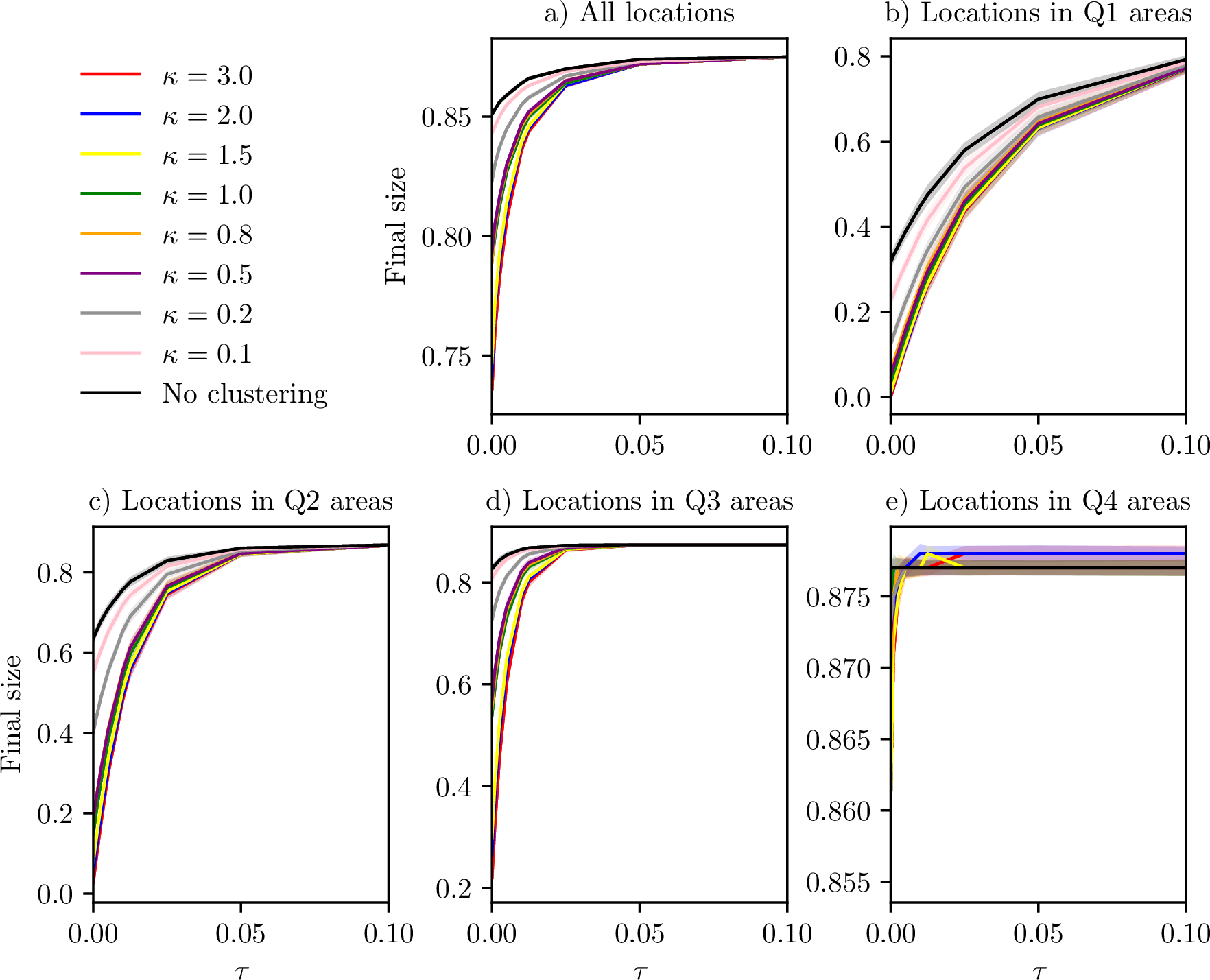

Supplement: S10 Fig — Final size versus τ for various clustering levels, κ, for the alternative disease parameters, with corresponding 95% confidence bands. a) All locations. b) Locations with population size smaller than the 25% quantile. c) Locations with population size between the 25% and 50% quantile. d) Locations with population size between the 50% quantile and the 75% quantile. e) Locations with population size larger than the 75% quantile. (TIF) [file pcbi.1006879.s011.tif]

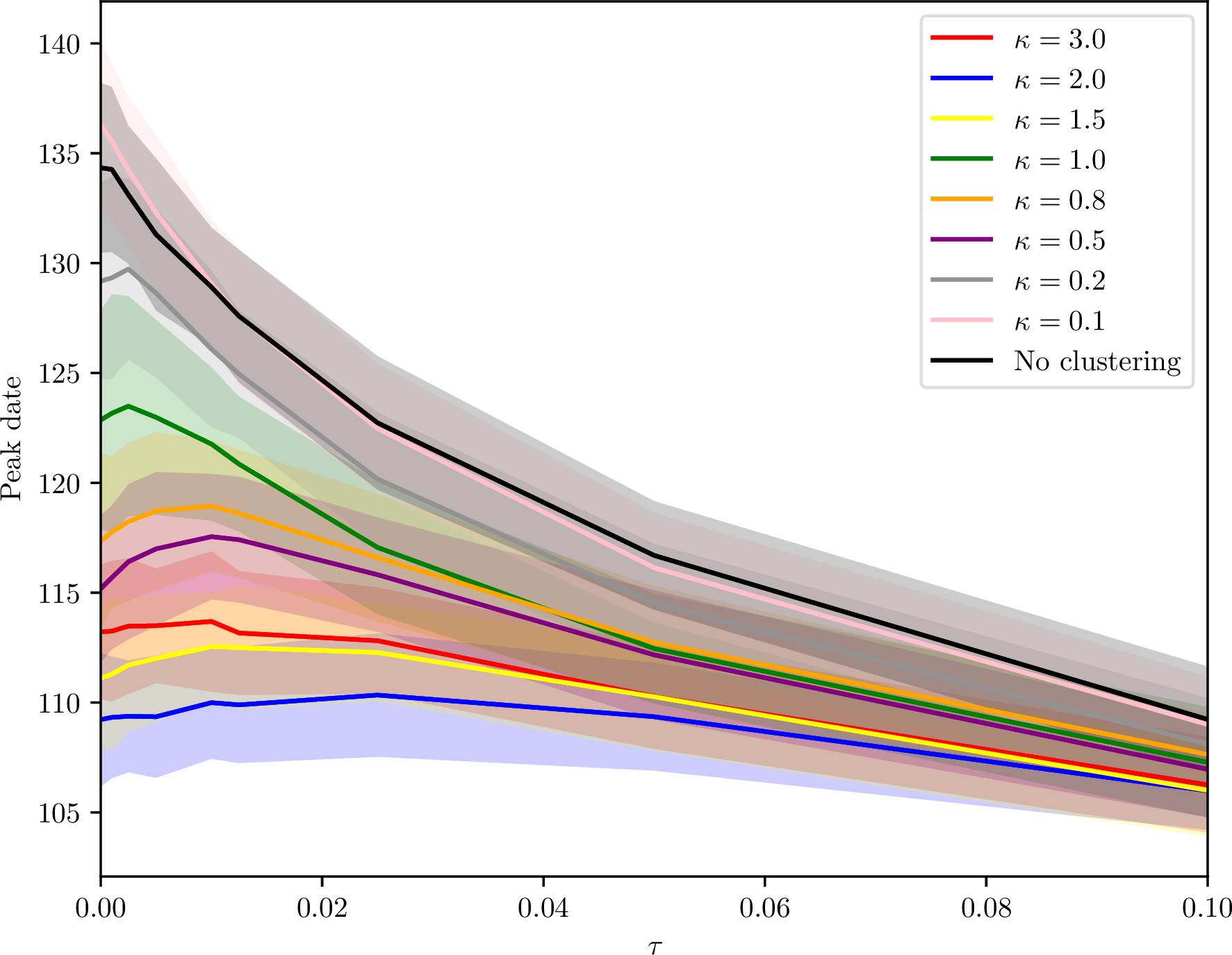

Supplement: S11 Fig — Peak date versus τ for various clustering levels, κ, with corresponding 95% confidence bands. The results are for the alternative disease parameters. (TIF) [file pcbi.1006879.s012.tif]

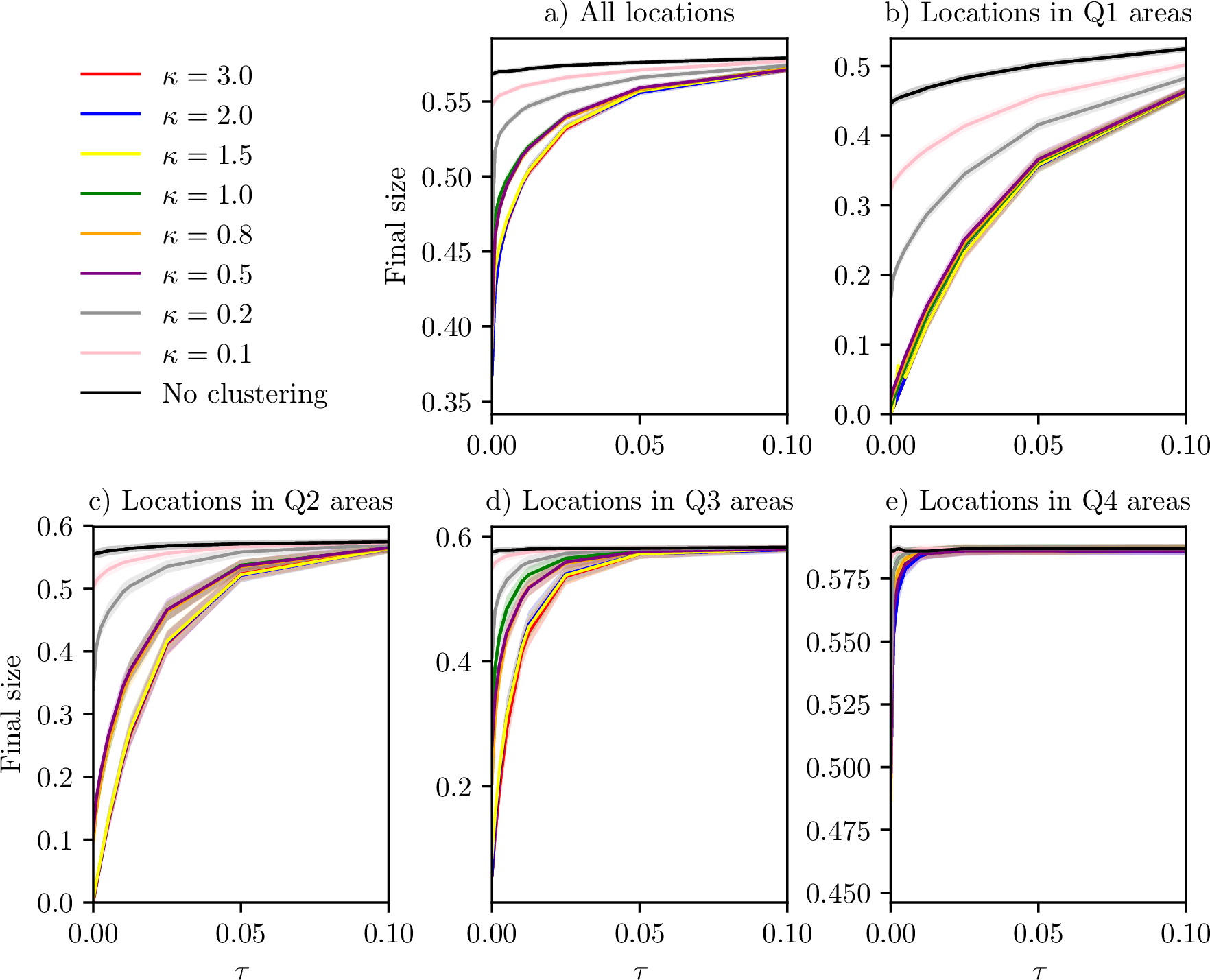

Supplement: S12 Fig — Final size versus τ for various clustering levels, κ, with corresponding 95% confidence bands. The results are in the countries based on data from the United Kingdom. a) All locations. b) Locations with population size smaller than the 25% quantile. c) Locations with population size between the 25% and 50% quantile. d) Locations with population size between the 50% quantile and the 75% quantile. e) Locations with population size larger than the 75% quantile. (TIF) [file pcbi.1006879.s013.tif]

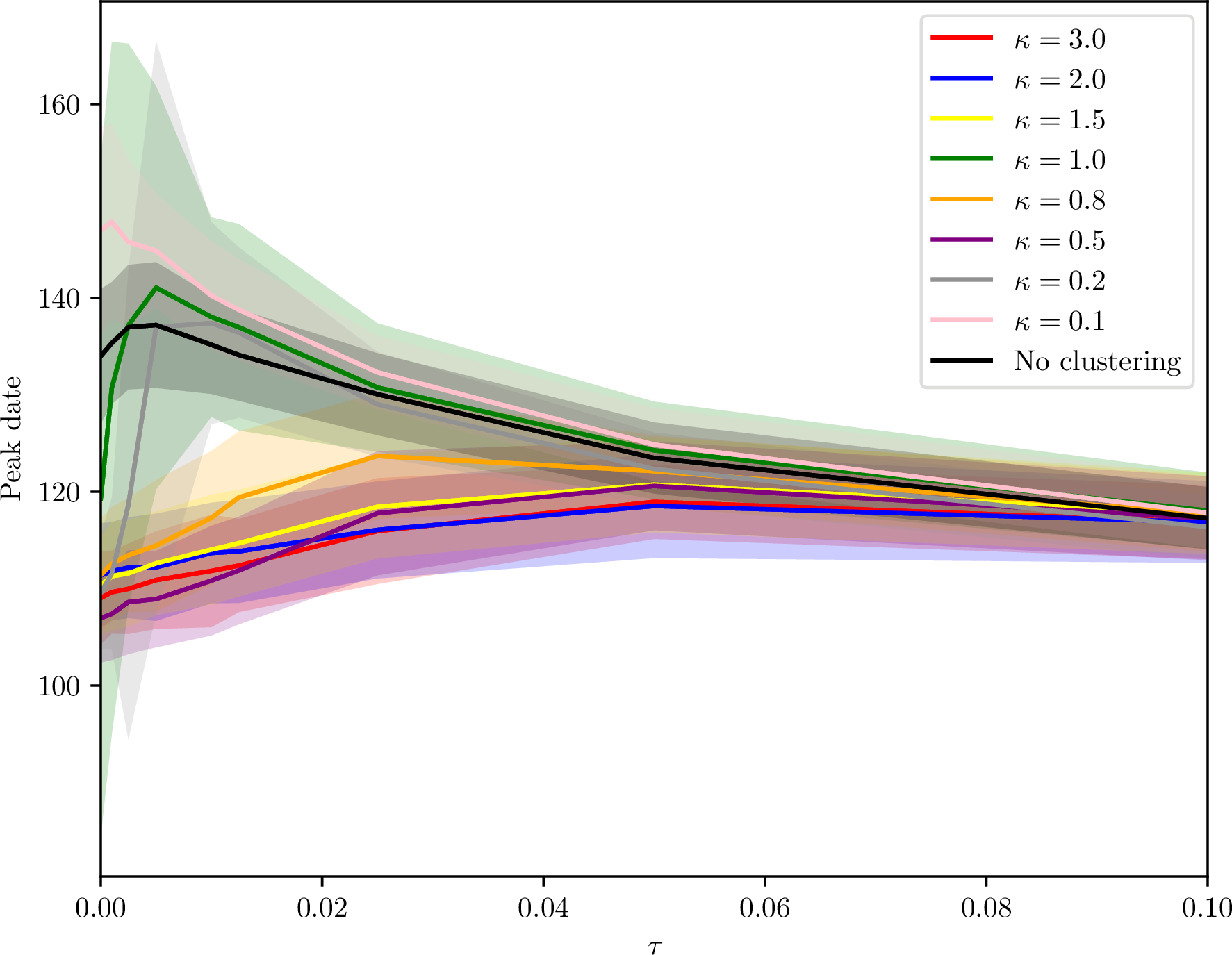

Supplement: S13 Fig — Peak date versus τ for various clustering levels, κ, with corresponding 95% confidence bands. The results are in the country based on data from the United Kingdom. (TIF) [file pcbi.1006879.s014.tif]

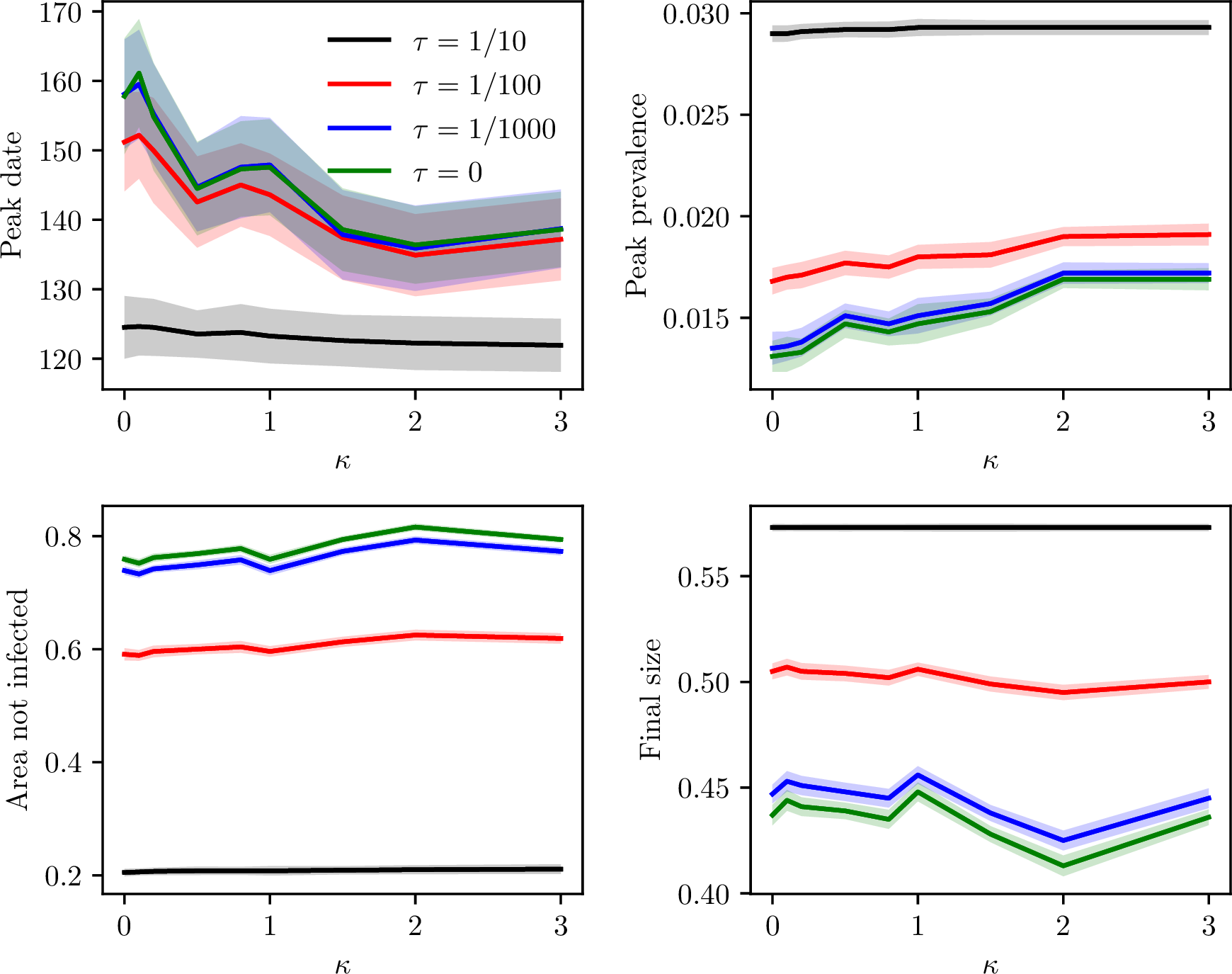

Supplement: S14 Fig — Peak dates for the global mean prevalence curve, peak prevalence, mean area not infected and mean final size as a function of clustering, with 95% confidence bands, when the distance parameter of the gravity law was halved. The lines correspond to the baseline scenario, 90% travel restrictions, 99% travel restrictions and 100% travel restrictions. Top left: peak date. Top right: peak prevalence. Bottom left: area not infected. Bottom right: final size. (TIF) [file pcbi.1006879.s015.tif]

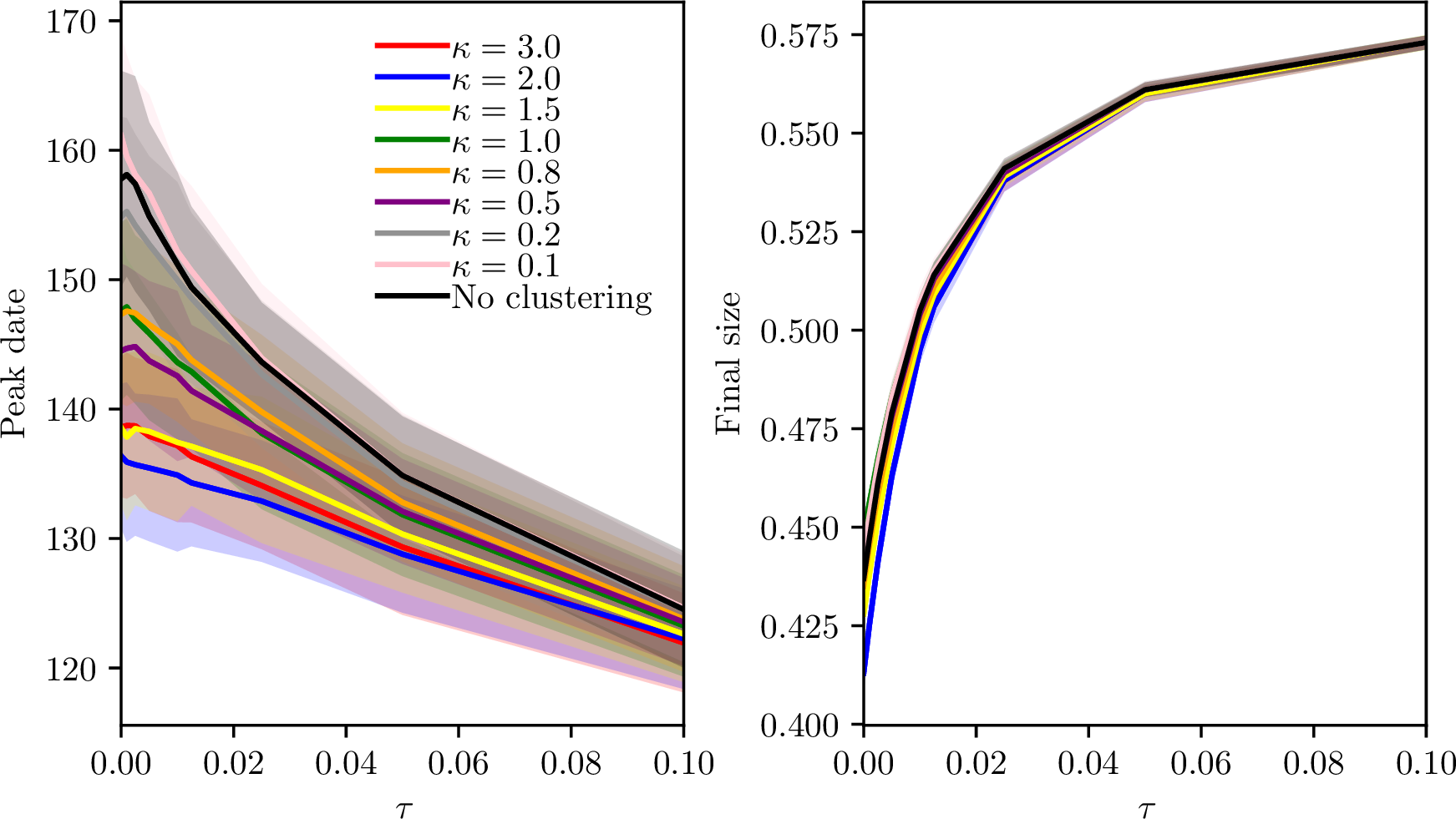

Supplement: S15 Fig — Final size and peak date versus τ for various clustering levels, κ, with corresponding 95% confidence bands, when the distance parameter of the gravity law was halved. (TIF) [file pcbi.1006879.s016.tif]

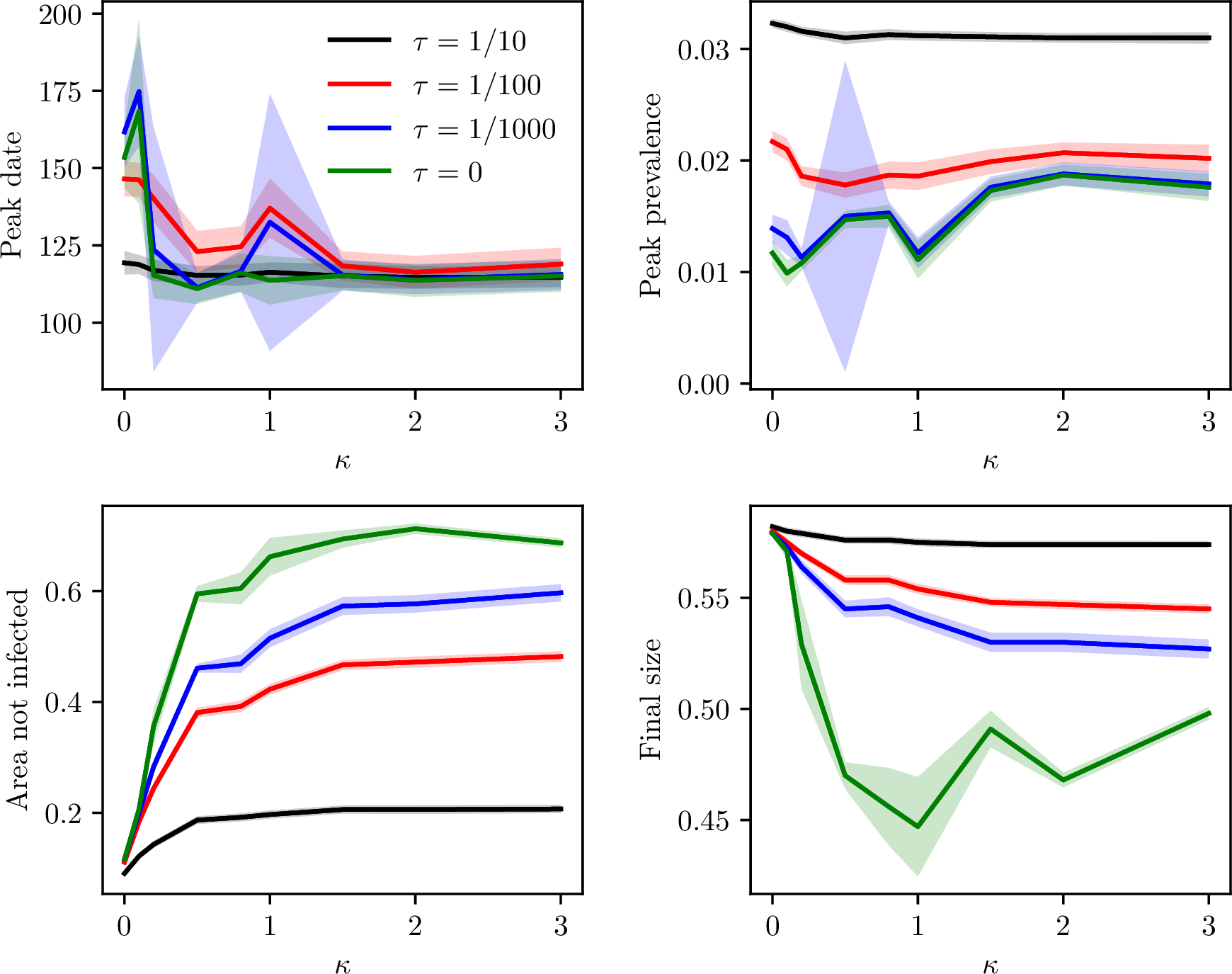

Supplement: S16 Fig — Peak dates for the global mean prevalence curve, peak prevalence, mean area not infected and mean final size as a function of clustering, with 95% confidence bands, when the distance parameter of the gravity law was doubled. The lines correspond to the baseline scenario, 90% travel restrictions, 99% travel restrictions and 100% travel restrictions. Top left: peak date. Top right: peak prevalence. Bottom left: area not infected. Bottom right: final size. (TIF) [file pcbi.1006879.s017.tif]

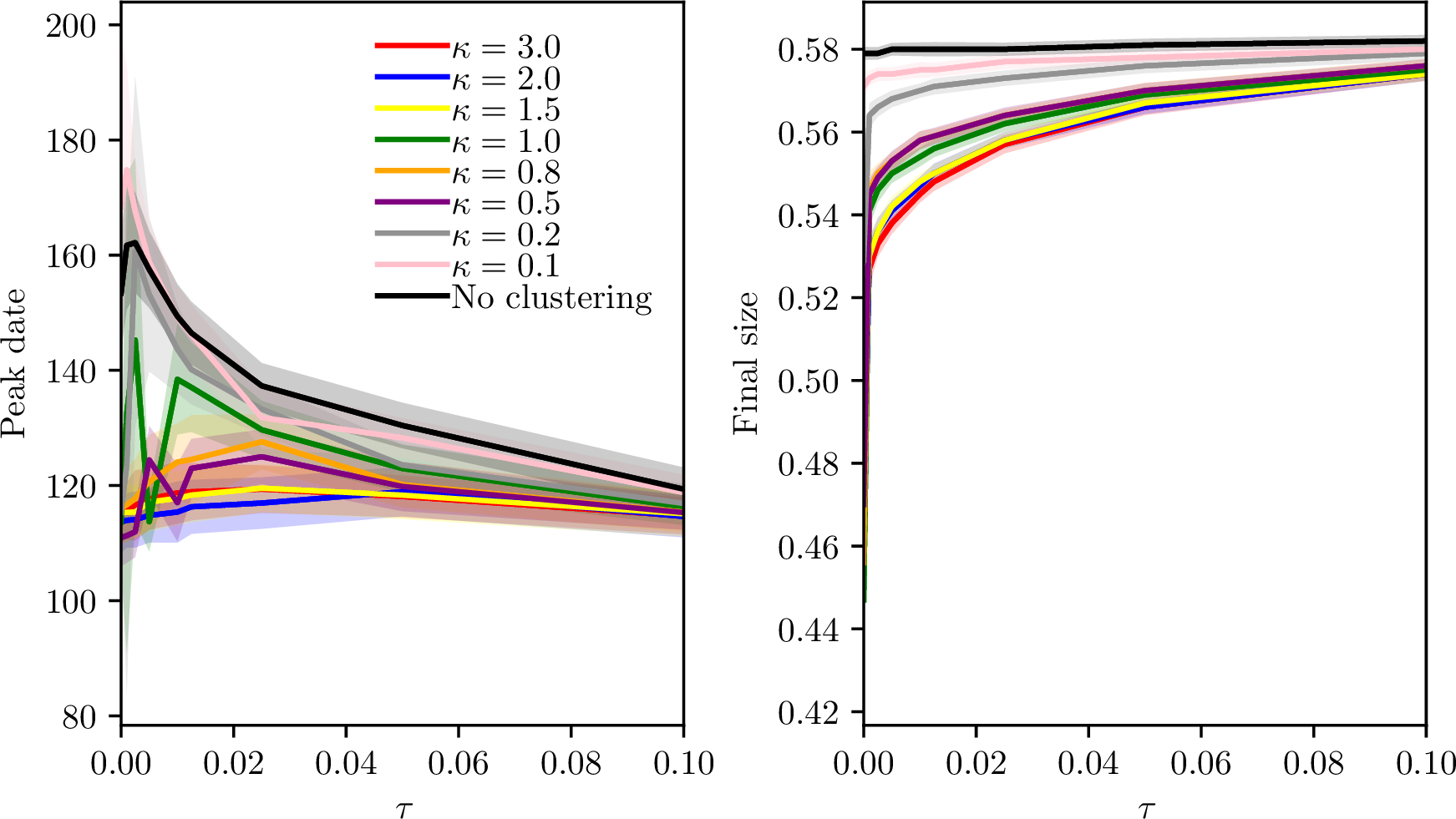

Supplement: S17 Fig — Final size and peak date versus τ for various clustering levels, κ, with corresponding 95% confidence bands, when the distance parameter of the gravity law was doubled. (TIF) [file pcbi.1006879.s018.tif]

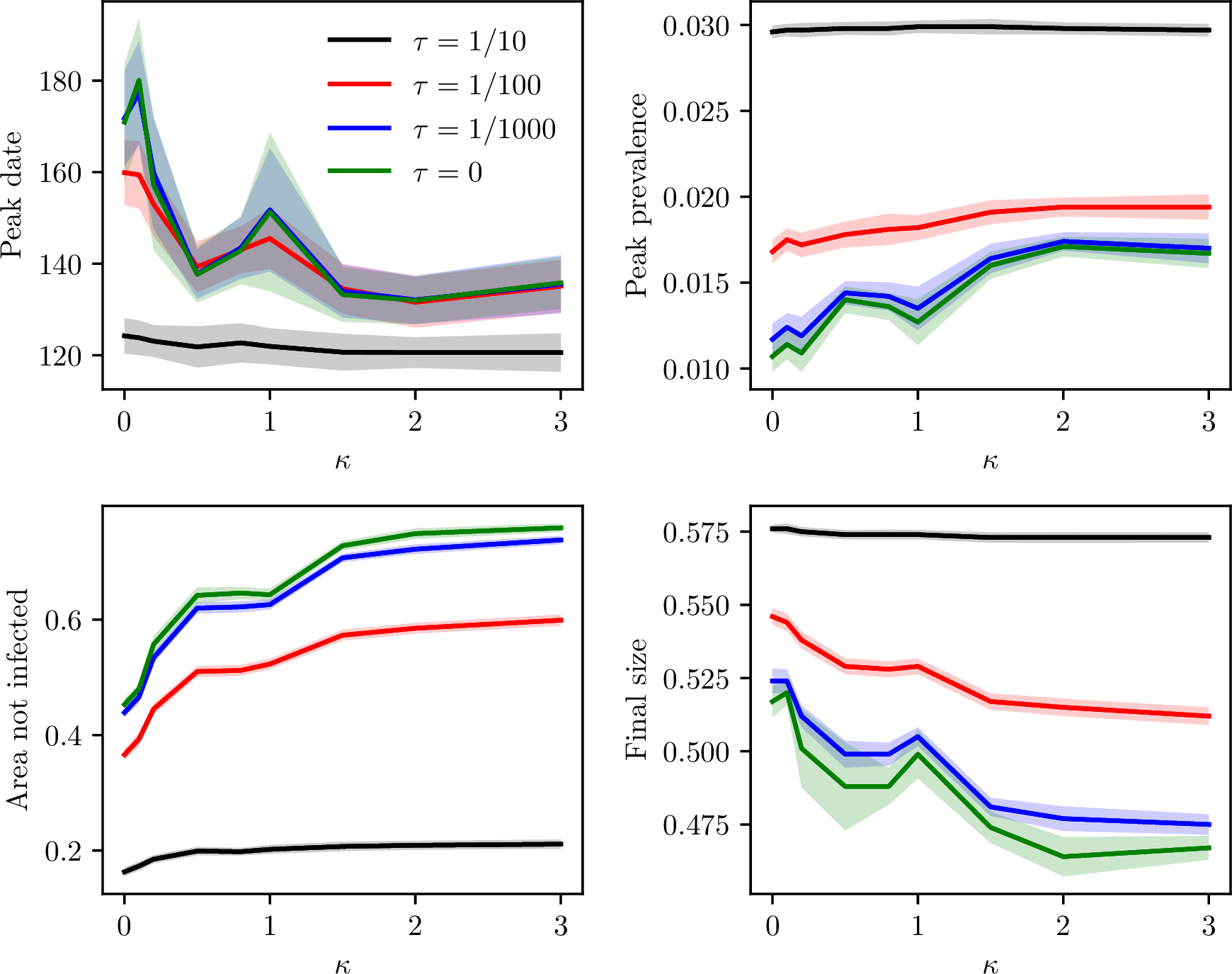

Supplement: S18 Fig — Peak dates for the global mean prevalence curve, peak prevalence, mean area not infected and mean final size as a function of clustering, with 95% confidence bands, when the destination population parameter of the gravity law was halved. The lines correspond to the baseline scenario, 90% travel restrictions, 99% travel restrictions and 100% travel restrictions. Top left: peak date. Top right: peak prevalence. Bottom left: area not infected. Bottom right: final size. (TIF) [file pcbi.1006879.s019.tif]

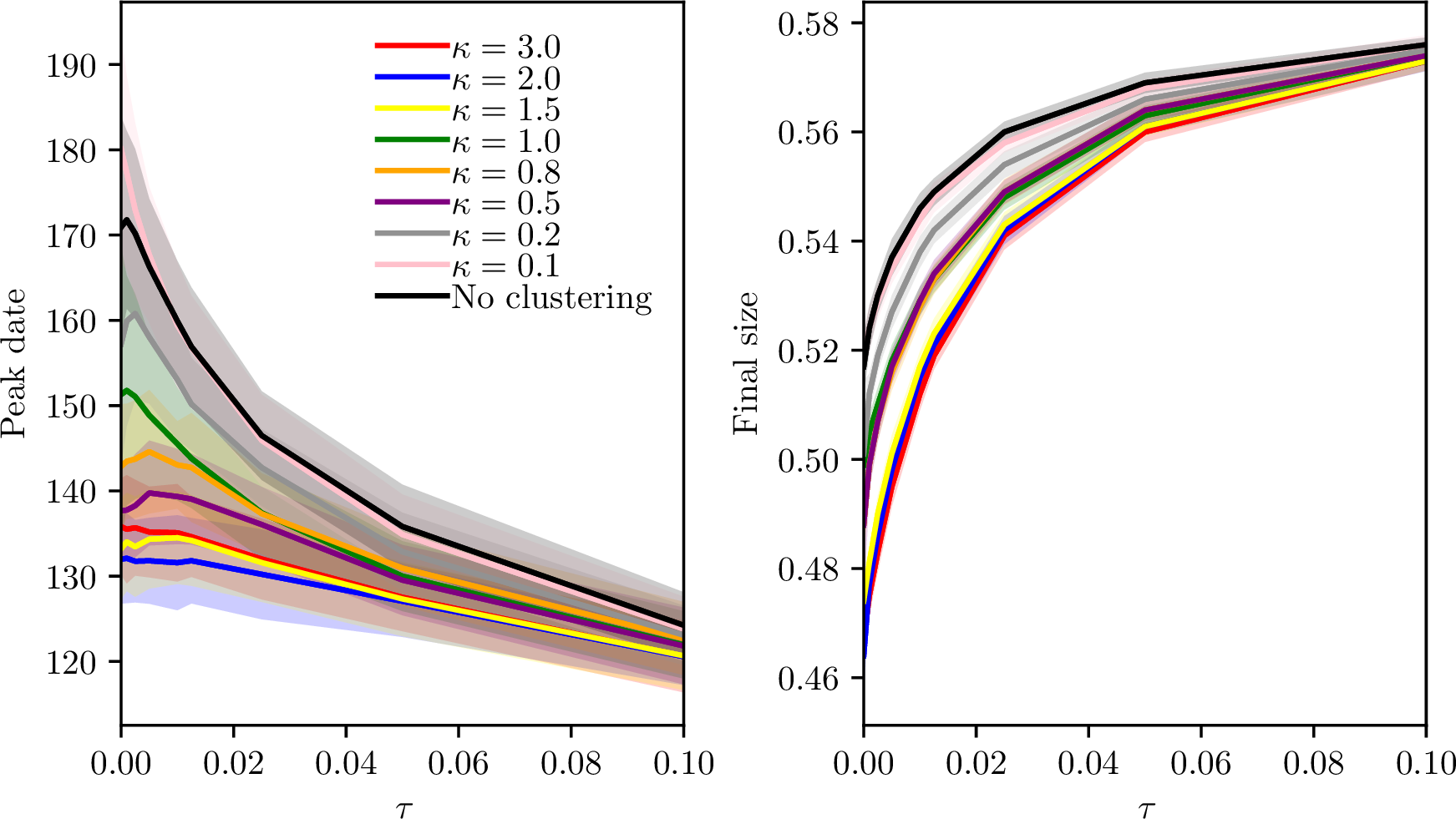

Supplement: S19 Fig — Final size and peak date versus τ for various clustering levels, κ, with corresponding 95% confidence bands, when the destination population parameter of the gravity law was halved. (TIF) [file pcbi.1006879.s020.tif]

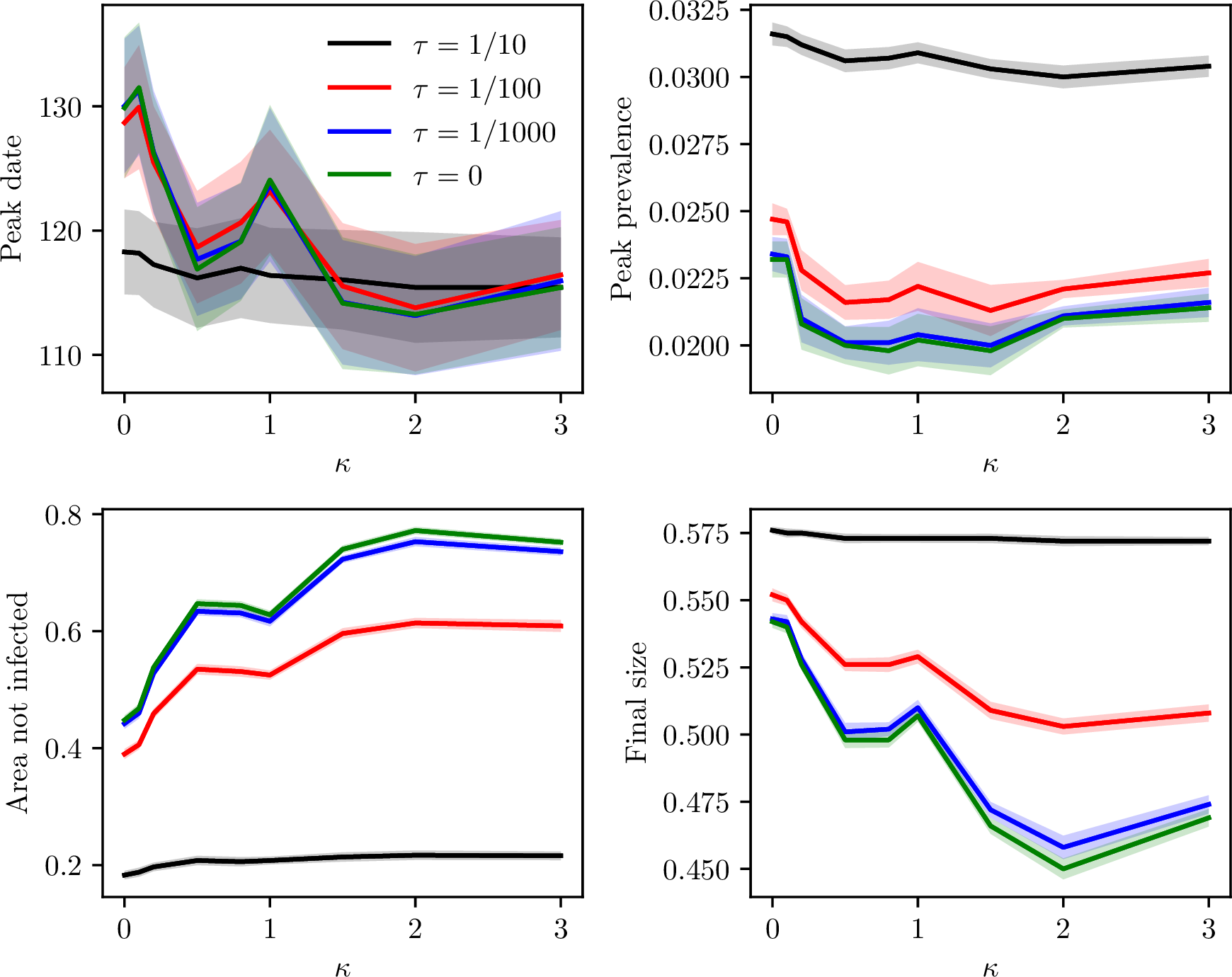

Supplement: S20 Fig — Peak dates for the global mean prevalence curve, peak prevalence, mean area not infected and mean final size as a function of clustering, with 95% confidence bands, when the destination population parameter of the gravity law was doubled. The lines correspond to the baseline scenario, 90% travel restrictions, 99% travel restrictions and 100% travel restrictions. Top left: peak date. Top right: peak prevalence. Bottom left: area not infected. Bottom right: final size. (TIF) [file pcbi.1006879.s021.tif]

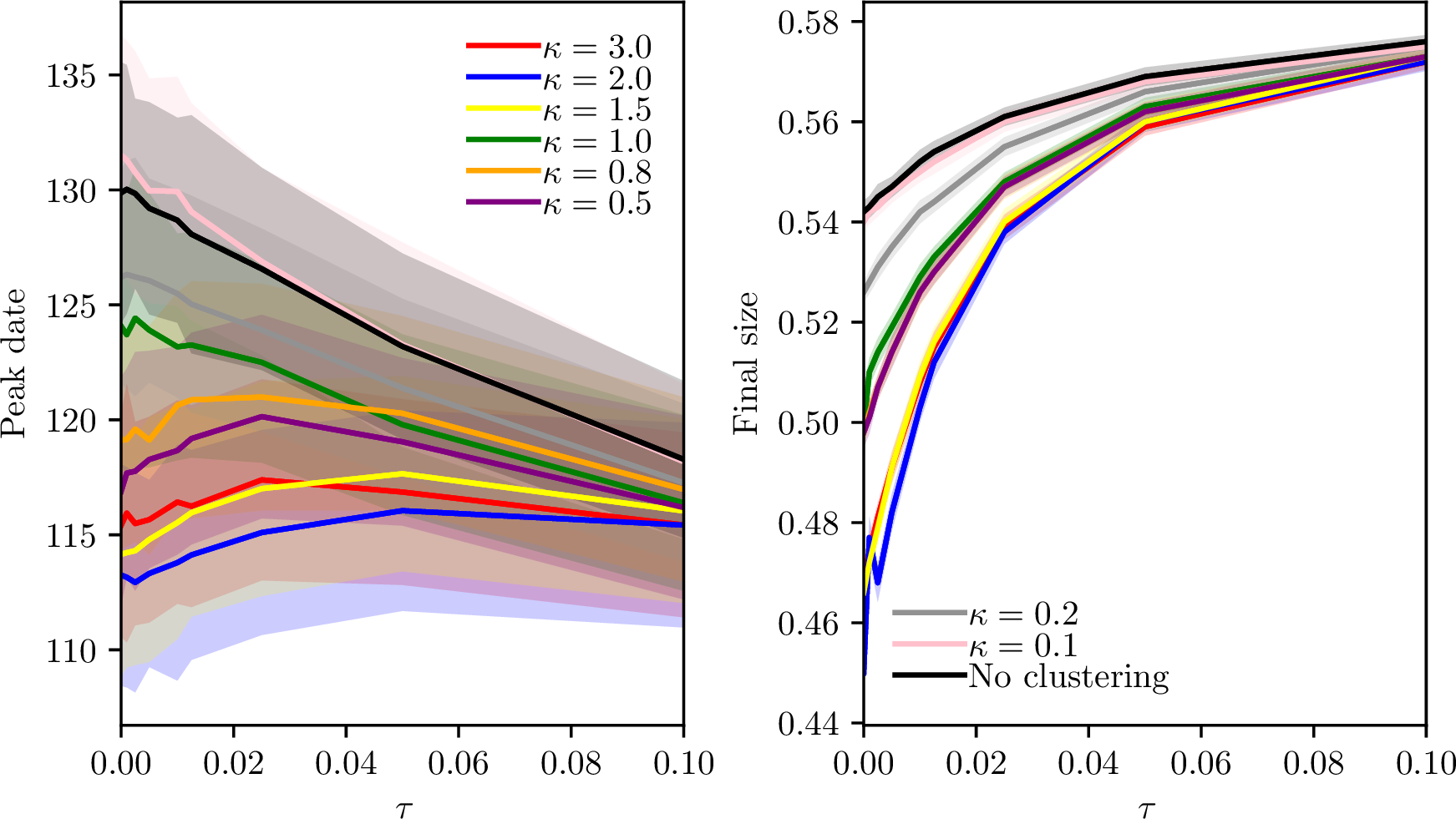

Supplement: S21 Fig — Final size and peak date versus τ for various clustering levels, κ, with corresponding 95% confidence bands, when the destination population parameter of the gravity law was doubled. (TIF) [file pcbi.1006879.s022.tif]

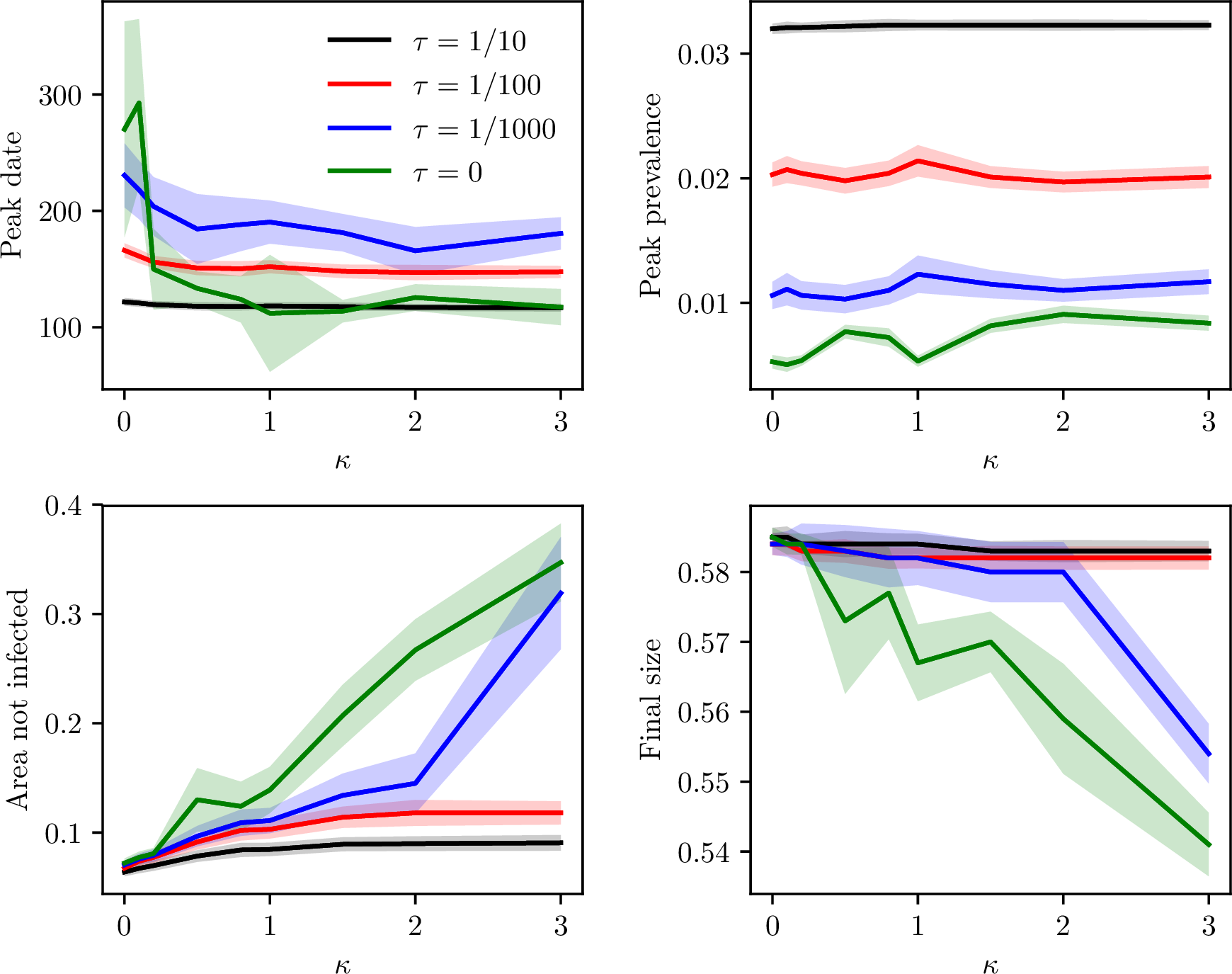

Supplement: S22 Fig — Peak dates for the global mean prevalence curve, peak prevalence, mean area not infected and mean final size as a function of clustering, with 95% confidence bands, with an exponential function of distance in the gravity law. The lines correspond to the baseline scenario, 90% travel restrictions, 99% travel restrictions and 100% travel restrictions. Top left: peak date. Top right: peak prevalence. Bottom left: area not infected. Bottom right: final size. (TIF) [file pcbi.1006879.s023.tif]

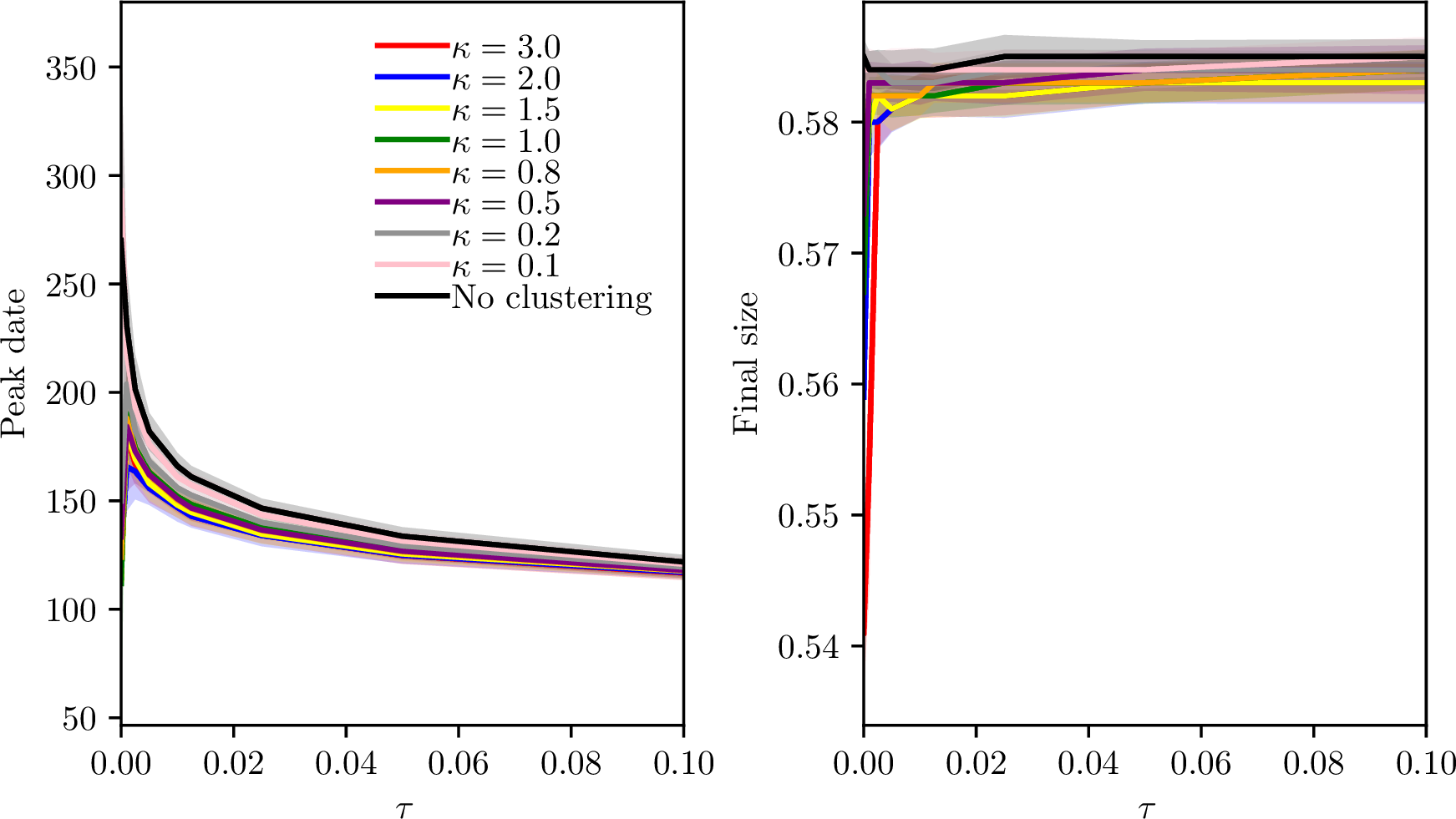

Supplement: S23 Fig — Final size and peak date versus τ for various clustering levels, κ, with corresponding 95% confidence bands, with an exponential function of distance in the gravity law. (TIF) [file pcbi.1006879.s024.tif]

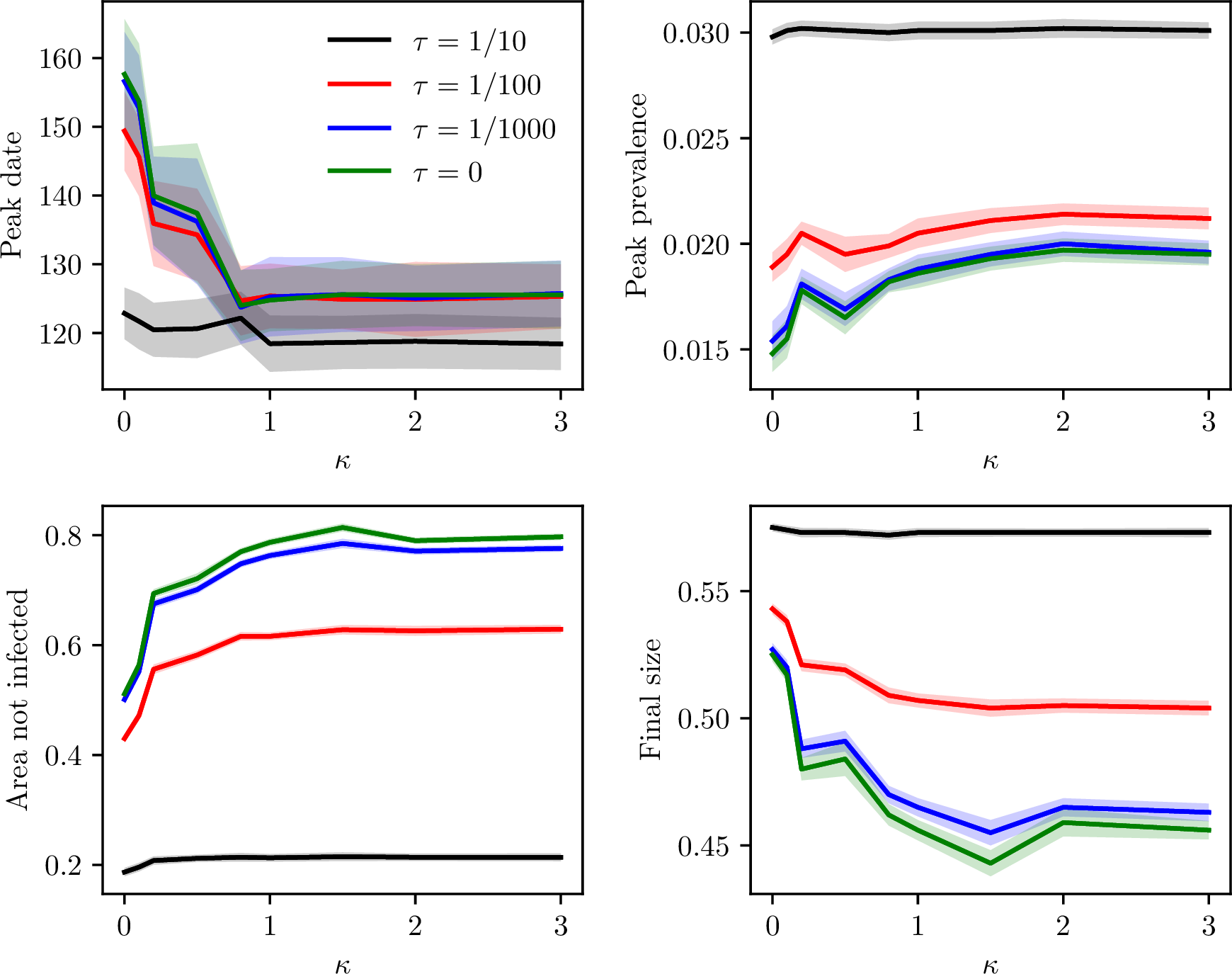

Supplement: S24 Fig — Peak dates for the global mean prevalence curve, peak prevalence, mean area not infected and mean final size as a function of clustering, with 95% confidence bands, when the range parameter of the Matérn covariance function was increased from 5.0 to 10.0. The lines correspond to the baseline scenario, 90% travel restrictions, 99% travel restrictions and 100% travel restrictions. Top left: peak date. Top right: peak prevalence. Bottom left: area not infected. Bottom right: final size. (TIF) [file pcbi.1006879.s025.tif]

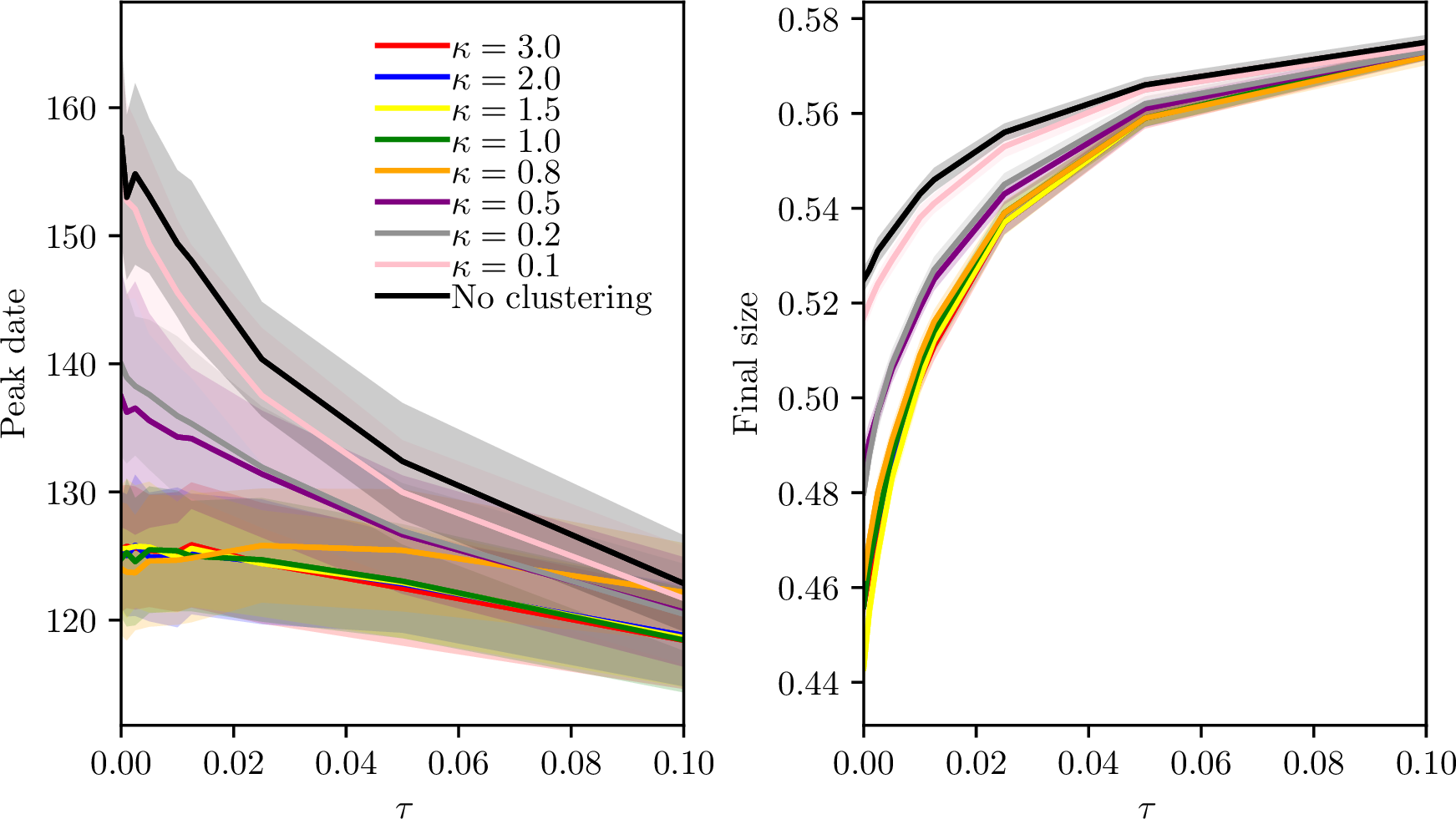

Supplement: S25 Fig — Final size and peak date versus τ for various clustering levels, κ, with corresponding 95% confidence bands, when the range parameter of the Matérn covariance function was increased from 5.0 to 10.0. (TIF) [file pcbi.1006879.s026.tif]

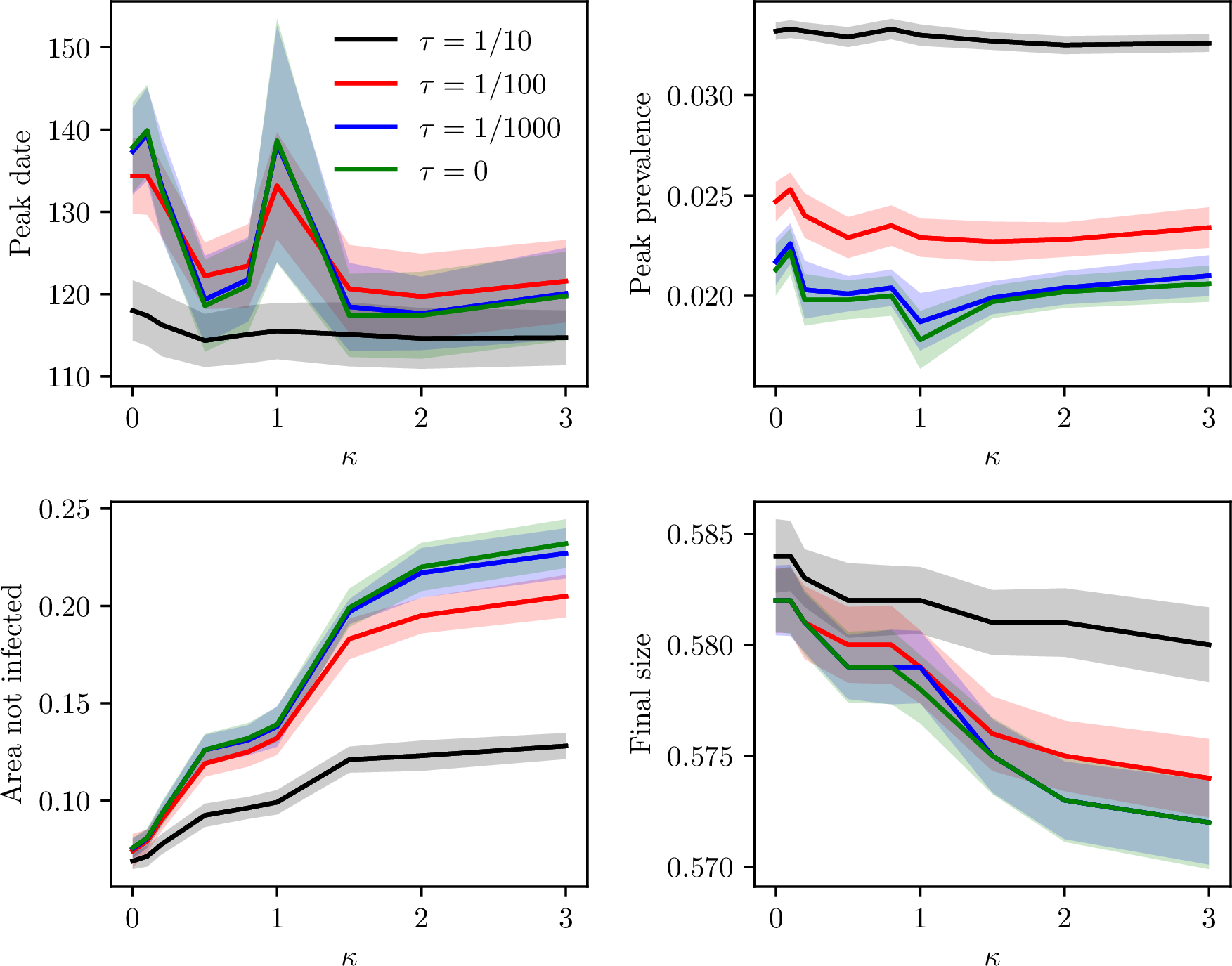

Supplement: S26 Fig — Peak dates for the global mean prevalence curve, peak prevalence, mean area not infected and mean final size as a function of clustering, with 95% confidence bands, when the commuting was implemented by the radiation law. The lines correspond to the baseline scenario, 90% travel restrictions, 99% travel restrictions and 100% travel restrictions. Top left: peak date. Top right: peak prevalence. Bottom left: area not infected. Bottom right: final size. (TIF) [file pcbi.1006879.s027.tif]

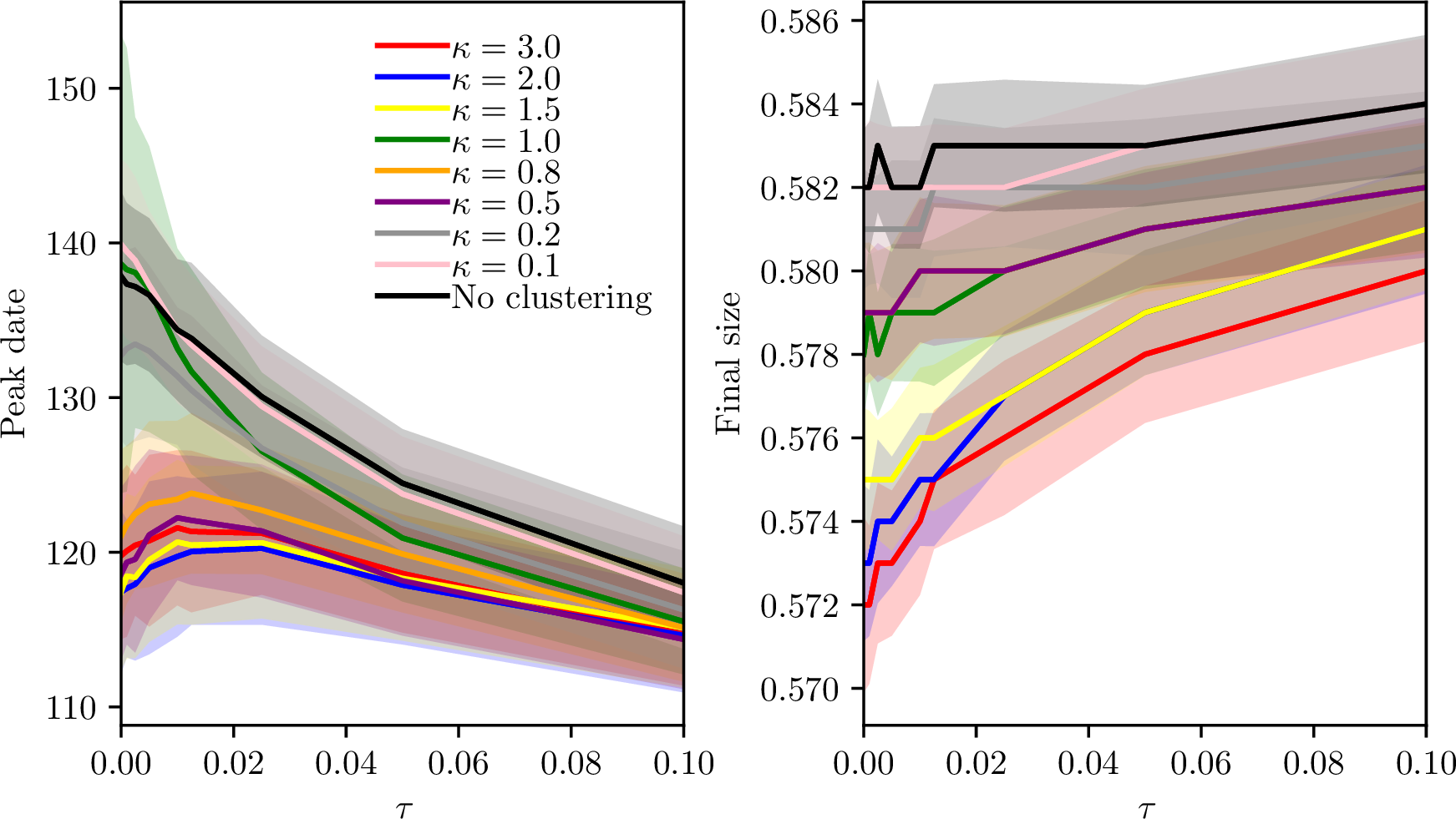

Supplement: S27 Fig — Final size and peak date versus τ for various clustering levels, κ, with corresponding 95% confidence bands, for the results where commuting was implemented by the radiation law. (TIF) [file pcbi.1006879.s028.tif]

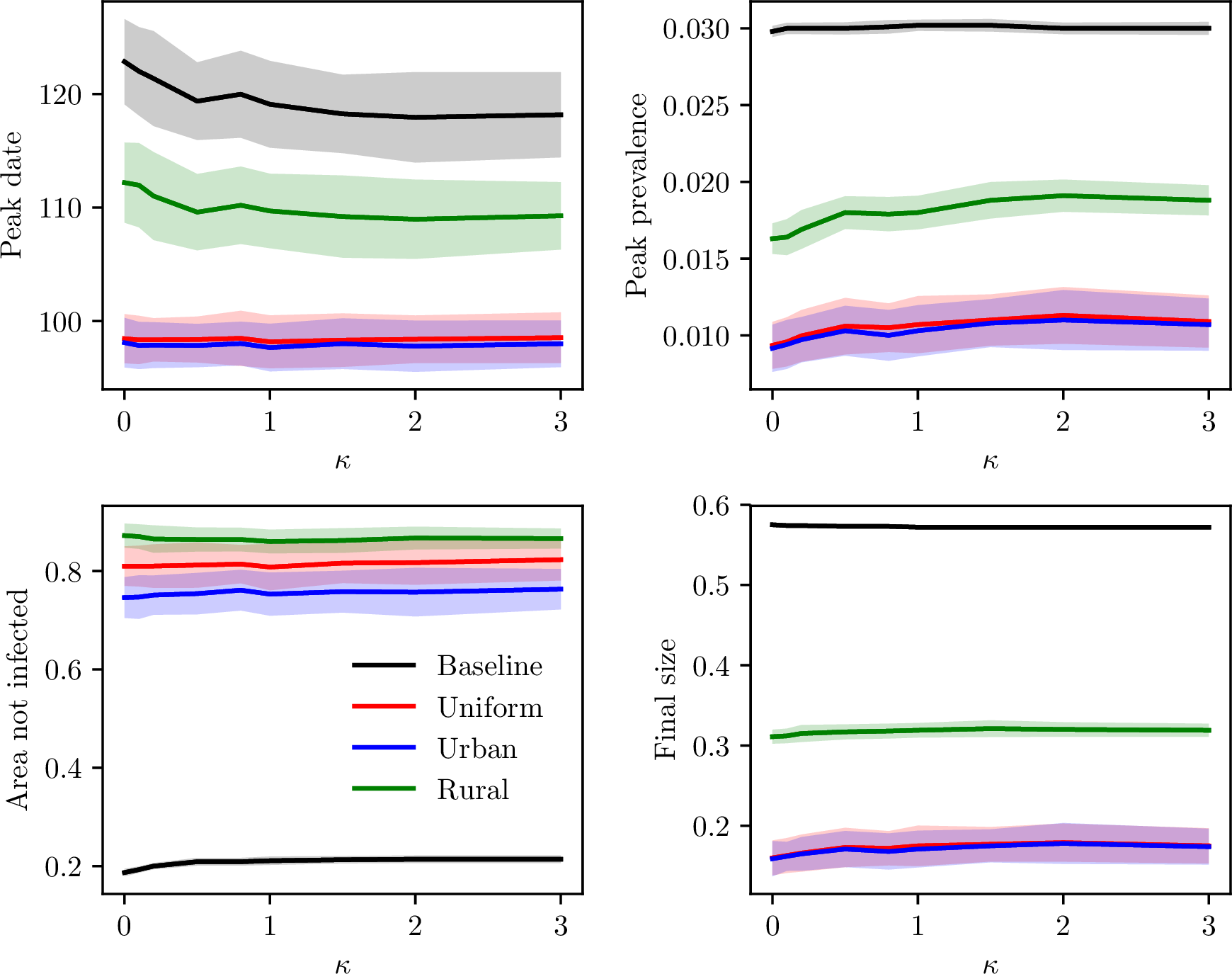

Supplement: S28 Fig — Peak dates for the global mean prevalence curve, peak prevalence, mean area not infected and mean final size as a function of clustering, with 95% confidence bands, when the assumed infectiousness of non-immune vaccinated is reduced by 20%. The lines correspond to the baseline scenario, uniform vaccination, urban vaccination and rural vaccination. Top left: peak date. Top right: peak prevalence. Bottom left: area not infected. Bottom right: final size. (TIF) [file pcbi.1006879.s029.tif]
