# Supplementary material for: A theoretical single-parameter model for urbanisation to study infectious disease spread and interventions
Source: PLoS Comput Biol. 2019 Mar 7;15(3):e1006879. doi: 10.1371/journal.pcbi.1006879 (PMC6424465; doi:10.1371/journal.pcbi.1006879)
Supplement: S1 Table — Estimated power a for final size = τa+ b, for different levels of clustering, for the Q1 (most rural), Q2, Q3 and Q4 (most urban) locations. (PDF) [file pcbi.1006879.s030.pdf]

Estimated a for urban and rural locations.

κ	Q1	Q2	Q3	Q4
No clustering	0.599	0.512	0.640	2.05
0.1	0.573	0.476	0.603	2.00
0.2	0.541	0.401	0.402	1.47
0.5	0.540	0.390	0.379	1.67
0.8	0.543	0.391	0.369	1.68
1.0	0.539	0.385	0.368	1.92
1.5	0.535	0.377	0.307	1.53
2.0	0.535	0.374	0.302	1.37
3.0	0.532	0.371	0.302	1.47

Estimated power a for final size $= \tau^a + b$, for different levels of clustering, for the Q1 (most rural), Q2, Q3 and Q4 (most urban) locations.

S1 Table
