# Supplementary material for: A theoretical single-parameter model for urbanisation to study infectious disease spread and interventions
Source: PLoS Comput Biol. 2019 Mar 7;15(3):e1006879. doi: 10.1371/journal.pcbi.1006879 (PMC6424465; doi:10.1371/journal.pcbi.1006879)
Supplement: S2 Table — Estimated power a for final size = τa+ b, for different levels of clustering. (PDF) [file pcbi.1006879.s031.pdf]

Estimated a for all locations.

$\boldsymbol{\kappa}$	No clustering	0.1	0.2	0.5	0.8	1.0	1.5	2.0	3.0
**a**	1.31	1.27	1.14	1.03	1.02	1.07	0.919	0.862	0.891

Estimated power a for final size $= \tau^a + b$, for different levels of clustering.

S2 Table
