# Supplementary material for: A theoretical single-parameter model for urbanisation to study infectious disease spread and interventions
Source: PLoS Comput Biol. 2019 Mar 7;15(3):e1006879. doi: 10.1371/journal.pcbi.1006879 (PMC6424465; doi:10.1371/journal.pcbi.1006879)
Supplement: S3 Table — Global peak day, global peak prevalence, percentage of area not infected and final sizes in the situation with a delay in the implementation of the travel restrictions. Standard deviations are given in parenthesis. (PDF) [file pcbi.1006879.s032.pdf]

Delayed travel restrictions.

κ	Peak day	Peak prevalence	Area not infected	Final size
No clustering	132.7 (3.5)	0.0172 (0.0004)	0.498 (0.00490)	0.527 (0.00176)
0.1	130.8 (3.5)	0.0181 (0.0005)	0.524 (0.00446)	0.525 (0.00144)
0.2	125.8 (3.2)	0.0183 (0.0004)	0.580 (0.00403)	0.513 (0.00169)
0.5	118.7 (2.7)	0.0193 (0.0004)	0.661 (0.00426)	0.495 (0.00192)
0.8	120.5 (2.6)	0.0196 (0.0004)	0.667 (0.00423)	0.494 (0.00193)
1.0	121.1 (2.8)	0.0200 (0.0004)	0.668 (0.00402)	0.498 (0.00166)
1.5	116.8 (2.5)	0.0207 (0.0004)	0.739 (0.00405)	0.474 (0.00188)
2.0	115.1 (2.3)	0.0206 (0.0003)	0.753 (0.00431)	0.466 (0.00237)
3.0	116.7 (2.1)	0.0206 (0.0004)	0.758 (0.00371)	0.467 (0.00195)

Global peak day, global peak prevalence, percentage of area not infected and final sizes in the situation with a delay in the implementation of the travel restrictions. Standard deviations are given in parenthesis.

S3 Table
